# The effect of dark states on the intersystem crossing and thermally activated delayed fluorescence of naphthalimide-phenothiazine dyads

**DOI:** 10.3762/bjoc.19.79

**Published:** 2023-07-19

**Authors:** Liyuan Cao, Xi Liu, Xue Zhang, Jianzhang Zhao, Fabiao Yu, Yan Wan

**Affiliations:** 1 State Key Laboratory of Fine Chemicals, Frontier Science Center for Smart Materials, School of Chemical Engineering, Dalian University of Technology, 2 Ling Gong Road, Dalian, 116024, P. R. China; 2 College of Chemistry, Beijing Normal University, Beijing 100875, P. R. China; 3 Key Laboratory of Hainan Trauma and Disaster Rescue, The First Affiliated Hospital of Hainan Medical University, Hainan Medical University, Haikou 571199, P. R. China

**Keywords:** charge-transfer, electron donor, intersystem crossing, TADF, triplet state

## Abstract

A series of 1,8-naphthalimide (NI)-phenothiazine (PTZ) electron donor–acceptor dyads were prepared to study the thermally activated delayed fluorescence (TADF) properties of the dyads, from a point of view of detection of the various transient species. The photophysical properties of the dyads were tuned by changing the electron-donating and the electron-withdrawing capability of the PTZ and NI moieties, respectively, by oxidation of the PTZ unit, or by using different aryl substituents attached to the NI unit. This tuning effect was manifested in the UV–vis absorption and fluorescence emission spectra, e.g., in the change of the charge transfer absorption bands. TADF was observed for the dyads containing the native PTZ unit, and the prompt and delayed fluorescence lifetimes changed with different aryl substituents on the imide part. In polar solvents, no TADF was observed. For the dyads with the PTZ unit oxidized, no TADF was observed as well. Femtosecond transient absorption spectra showed that the charge separation takes ca. 0.6 ps, and admixtures of locally excited (^3^LE) state and charge separated (^1^CS/^3^CS) states formed (in *n*-hexane). The subsequent charge recombination from the ^1^CS state takes ca. 7.92 ns. Upon oxidation of the PTZ unit, the beginning of charge separation is at 178 fs and formation of ^3^LE state takes 4.53 ns. Nanosecond transient absorption (ns-TA) spectra showed that both ^3^CS and ^3^LE states were observed for the dyads showing TADF, whereas only ^3^LE or ^3^CS states were observed for the systems lacking TADF. This is a rare but unambiguous experimental evidence that the spin–vibronic coupling of ^3^CS/^3^LE states is crucial for TADF. Without the mediating effect of the ^3^LE state, no TADF is resulted, even if the long-lived ^3^CS state is populated (lifetime τ_CS_ ≈ 140 ns). This experimental result confirms the ^3^CS → ^1^CS reverse intersystem crossing (rISC) is slow, without coupling with an approximate ^3^LE state. These studies are useful for an in-depth understanding of the photophysical mechanisms of the TADF emitters, as well as for molecular structure design of new electron donor–acceptor TADF emitters.

## Introduction

Thermally activated delayed fluorescence (TADF) compounds are promising emitters to be used in organic light-emitting diodes (OLED) [1–12]. These emitters have the advantage of low cost and high harvesting efficiency of both the singlet and triplet excitons and thus a high quantum efficiency for the electroluminescence [13]. The TADF emission process involves the reverse intersystem crossing (rISC) from the triplet (T_1_) state to the emissive singlet (S_1_) state. A typical molecular structure motif for this kind of emitters is an electron donor–acceptor dyad, and the molecular geometry is usually orthogonal, i.e., the planes of the π-conjugation systems of the electron donor and acceptor are perpendicular to each other. As a result, the highest occupied molecular orbital (HOMO) and lowest unoccupied molecular orbital (LUMO) are spatially separated. This molecular geometry is beneficial to reduce the energy gap of the S_1_/T_1_ states, making the rISC possible. Because of the orthogonal molecular orbitals, the electron exchange energy (*J*) of the two unpaired electrons is small, which leads to a small energy gap (2*J*) of the S_1_/T_1_ states. Moreover, the orthogonal geometry is beneficial for the intersystem crossing (ISC) of the dyads, i.e., via the spin–orbit charge transfer ISC (SOCT-ISC) [14–18]. It can be considered as a generalization of the El-Sayed’s rule for ISC. This SOCT-ISC may also enhance the rISC in OLED devices, in which the electron–hole recombination produces mainly the triplet state (the theoretical probability is 75%, by following the spin statistic rule) [1].

Compared to the application studies, the investigation of the photophysical mechanism of TADF emitters is far from mature [19–21]. Initially a two-state model was used for understanding of the photophysical processes of the electron donor–acceptor TADF emitters [9,22–26]. Soon it was realized this over simplified model is nonsufficient. For instance, the singlet/triplet charge-separated states ^1^CS and ^3^CS of these emitters are believed to undergo nonefficient slow interconversion, thus the poor rISC will not lead to efficient TADF. Later, a three-state model was proposed, i.e., the ^1^CS, ^3^CS, and a locally excited (^3^LE) state should be involved in the TADF process. The rISC is facilitated by the ^3^LE state, which shares a similar energy with the ^3^CS state, e.g., the so called spin–vibronic coupling effect, and as a result, the rISC is fast and efficient, which can result in significant TADF [13,27–31]. It was also proposed that vibration facilitates the ISC in the TADF emitters [32–33]. However, the most popular optical spectroscopic methods previously used in this area, such as the very often used transient photoluminescence spectral measurement or the luminescence lifetime measurement, are unable to detect the dark states of the TADF emitters, i.e., the ^3^LE and ^3^CS states. Femtosecond transient absorption spectroscopy (fs-TA) was rarely used for the study of the photophysics of the TADF emitters [12,34–37], since it suffers from the limitation of the time window of detection (up to a few ns). On the other hand, nanosecond transient absorption spectroscopy [38–44] and, more recently, time-resolved electron paramagnetic resonance (TREPR) spectroscopy [33,39,44–46] were also applied to study TADF mechanisms, but the examples are limited.

Therefore, much room is left for studies of the photophysical mechanism of the TADF emitters, especially to find experimental evidence of the spin–vibronic coupling effect, i.e., the involvement of the dark states, such as ^3^LE and ^3^CS states, in the TADF process of the electron donor–acceptor emitters.

In order to address the above challenge, herein we prepared a series of electron donor–acceptor dyads, based on naphthalimide (NI) as electron acceptor and phenothiazine (PTZ) as electron donor. The two units are connected by a C–N single bond, and they adopt orthogonal geometry due to conformational restriction. In order to tune the energy of the excited states, and to probe the effect of the energy ordering of the transient species involved in the TADF photophysical process, the electron-donating strength of the PTZ moiety is lowered by oxidation of the sulfur atom to the corresponding sulfoxide. Conversely, the electron-accepting capability of the NI unit is varied by introducing different aryl substituents, which contain both electron-donating and -withdrawing moieties. Although we reported **NI-PTZ** analogous dyads recently, they were not studied by fs-TA spectroscopy and the molecular structures are different from the current dyads [39,47]. The photophysical processes of the dyads were studied by steady state UV–vis absorption spectroscopy, transient photoluminescence spectroscopy, nanosecond/femtosecond transient absorption spectroscopy, electrochemistry, as well as DFT/TDDFT computations. We observed experimental evidence for the spin–vibronic coupling effect in the TADF photophysical processes of the electron donor–acceptor emitters.

## Results and Discussion

### Molecular design and structure confirmation

**Scheme 1:**
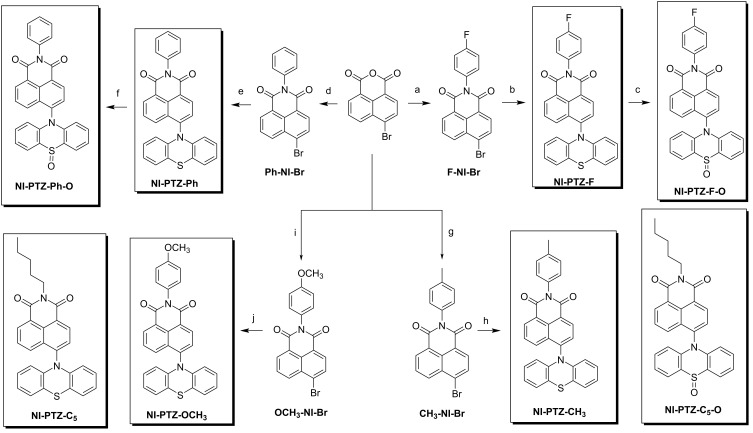
Synthesis of the compounds. Conditions: (a) 4-fluoroaniline, acetic acid, N_2_, reflux, 7 h, yield: 72%; (b) phenothiazine, sodium *tert*-butoxide, dry toluene, tri-*tert*-butylphosphine tetrafluoroborate, Pd(OAc)_2_, 120 °C, 8 h, yield: 15%; (c) H_2_O_2_ (30%), CH_3_COOH, 40 °C, 1 h, yield: 76%; (d) aniline, acetic acid, N_2_, reflux, 7 h; yield: 89%; (e) similar to step (b), yield: 52%; (f) similar to step (c), yield: 82%; (g) *p*-toluidine, acetic acid, N_2_, reflux, 7 h, yield: 83%; (h) similar to step (b), yield: 80%; (i) *p*-anisidine, acetic acid, N_2_, reflux, 7 h, yield: 75%; (j) similar to step (b), yield: 22%.

PTZ is a commonly used electron donor with strong electron-donating ability (*E*_OX_ = +0.18 V, vs Fc/Fc^+^) [48], while the NI chromophore is an electron acceptor (*E*_RED_ = −1.72 V, vs Fc/Fc^+^) [49]. By directly connecting the NI and PTZ unit through a C–N single bond, the π-conjugation plane of the NI and PTZ units adopts a perpendicular geometry which is beneficial for SOCT-ICS (Scheme 1). Previously we observed TADF with an analogue of **NI-PTZ-C_5_** (the difference of the molecular structures is the alkyl chain, we called that analogue **NI-PTZ-N** here) [39]. In order to study the effect of tuning the energy and the ordering of the excited states on the photophysical properties of the dyad, especially the ISC and TADF properties, we introduced electron-donating and -withdrawing aryl substituents on the NI unit (**NI-PTZ-F**, **NI-PTZ-Ph**, **NI-PTZ-CH_3_**, **NI-PTZ-OCH_3_**), and the electron-donating ability of the PTZ unit was modified by oxidation of the sulfur atom to the sulfoxide (**NI-PTZ-F-O**, **NI-PTZ-Ph-O**, **NI-PTZ-C_5_-O**). The advantage of the oxidation approach is that almost only the energy of the ^1^CS and ^3^CS states is changed, while other factors are kept intact to a large extent [46,50]. For instance, for the LE state, the electron exchange energy (*J*) for the unpaired electrons of the CS states (the energy gap of the ^1^CS/^3^CS state is 2*J*), the energy gap of the ^1^LE/^3^LE states of the NI moiety are not changed by the unique molecular structure modification method [51]. This approach is useful for studying molecules showing complicated, entangled photophysical processes upon photoexcitation, for instance the electron donor–acceptor type of TADF emitters [44,46]. Recently, we reported **NI-PTZ** analogous dyads, however, their molecular structures are different from the current dyads, and the photophysical properties were tuned by variation of the distance between the NI and the PTZ units, or by oxidation of the PTZ unit [47]. In the current dyads, we used different strategies.

A commonly used electron donor for the construction of electron donor–acceptor TADF molecules is carbazole. However, since our aim was to study the TADF photophysics with model TADF emitters, the use of phenothiazine (PTZ) instead of carbazole presents a few advantages. First, upon connection of the electron acceptor (i.e., the NI unit) to the N atom of phenothiazine, a more restricted geometry is achieved than using carbazole. This is beneficial for the orthogonal geometry and thus TADF is ensured. Second, as compared with carbazole, the use of phenothiazine in the construction of TADF emitters offers an additional dimension to tune the photophysical properties, i.e., the phenothiazine unit can be oxidized to the sulfoxide, thus tuning the energy states ordering between the ^1^CS, ^3^CS, and ^3^LE states. Moreover, the advantage of this molecular structure design is the ability to change only one factor (such as the CS state energy), while keeping other factors intact to a large extent (e.g., the LE state energy). This is an important advantage for the study of complicated photophysical mechanisms involved in TADF processes.

The syntheses of the dyads are based on the known derivatization chemistry of NI and PTZ chromophores, which are coupled through a Buchwald–Hartwig coupling reaction (another **NI-PTZ** paper) [39]. The oxidation of the PTZ unit was readily performed by treatment with H_2_O_2_ as oxidant (Scheme 1). All compounds were obtained with satisfactory yields and the molecular structures were fully characterized by ^1^H NMR, ^13^C NMR, and HRMS spectra (Experimental section).

### UV–vis absorption and fluorescence emission spectra

**Figure 1:**
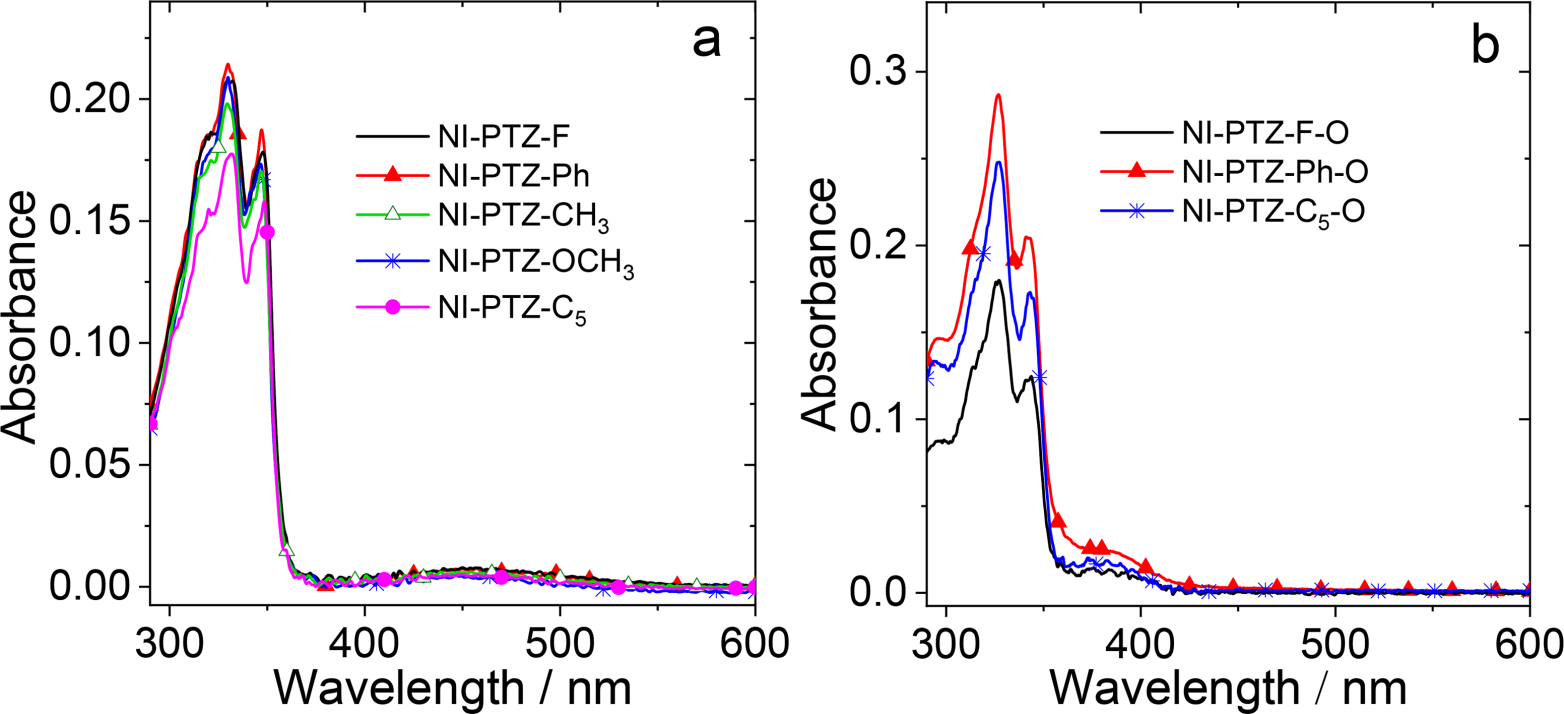
UV–vis absorption spectra of (a) **NI-PTZ-F**, **NI-PTZ-Ph**, **NI-PTZ-CH_3_**, **NI-PTZ-OCH_3_**, and **NI-PTZ-C_5_** and (b) **NI-PTZ-F-O**, **NI-PTZ-Ph-O**, and **NI-PTZ-C_5_-O** in *n*-hexane (HEX), *c* = 1.0 × 10^−5^ M, 20 °C.

The UV–vis absorption spectra of the compounds were studied (Figure 1 and Figure S29 in Supporting Information File 1). For the compounds without an oxidized PTZ unit, there are characteristic absorption bands in the 300–375 nm range, which are attributed to the NI moiety, i.e., originating from the S_0_ → ^1^LE transition (Figure 1a). Moreover, in the range of 375–570 nm, there is a weak, broad absorption band centered at 460 nm, similar to the analogue **NI-PTZ-C_5_** [39], which is assigned to the S_0_ → ^1^CS transition [52–54]. In addition, the aromatic ring attached to the NI unit changing it from an electron-pulling group to an electron-pushing group, leads to a blue shift of the absorption peak of the CS and the absorbance decreases (Figure S30 in Supporting Information File 1). For the oxidized molecules, the S_0_ → ^1^CS transition absorption peak is blue shifted to 360–420 nm (Figure 1b). These results indicate that upon oxidation of the PTZ unit, the electronic coupling between the donor and acceptor is reduced [55].

**Figure 2:**
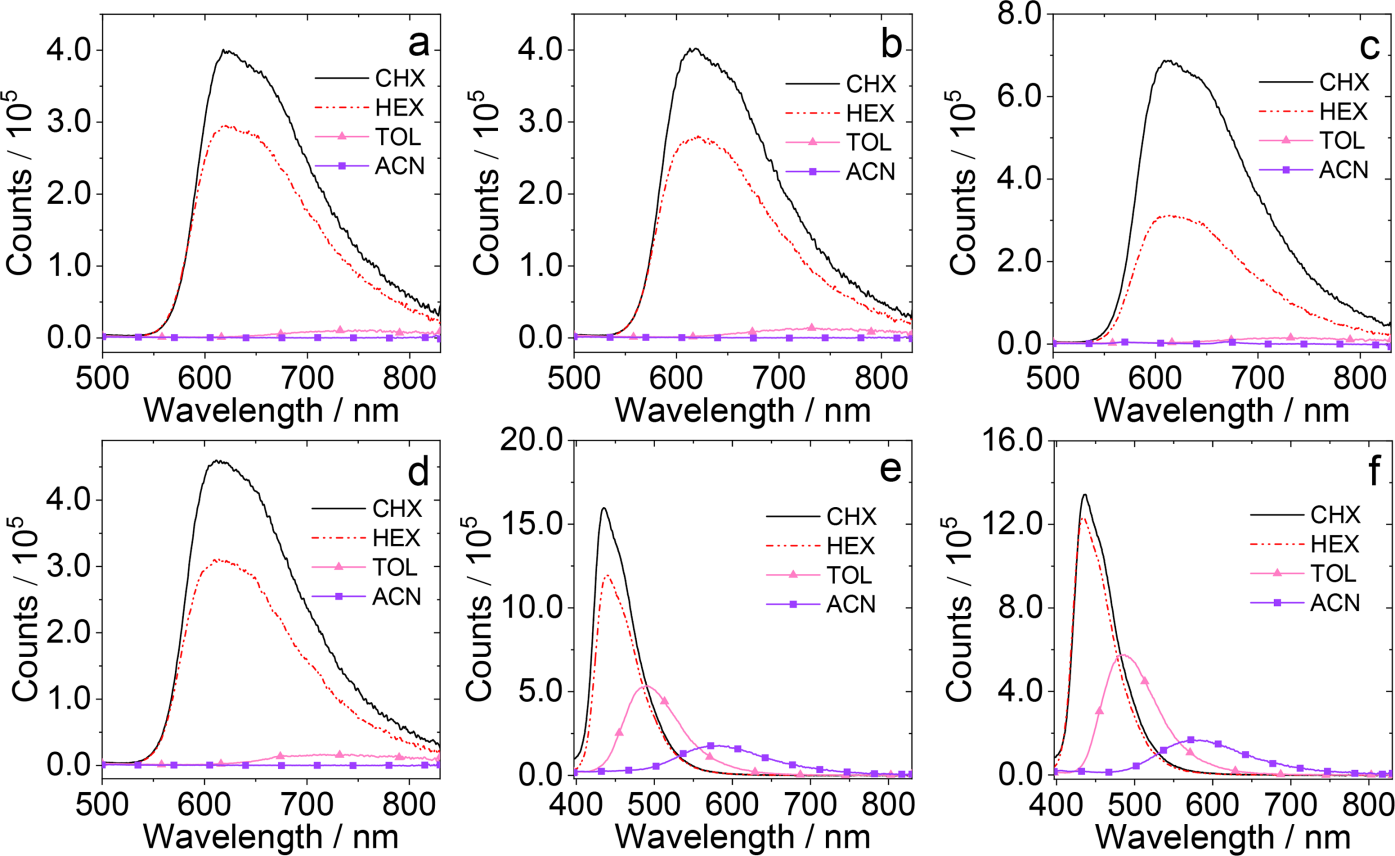
Fluorescence spectra of the dyads. (a) **NI-PTZ-F**, (b) **NI-PTZ-Ph**, (c) **NI-PTZ-CH_3_**, (d) **NI-PTZ-OCH_3_**, (e) **NI-PTZ-F-O**, and (f) **NI-PTZ-Ph-O** in different solvents. The solvents used were: CHX, HEX, toluene (TOL) and acetonitrile (ACN). Optically-matched solutions were used, *A* = 0.107, λ_ex_ = 310 nm, 20 °C.

The fluorescence spectra of the compounds are studied (Figure 2 and Figure S31 in Supporting Information File 1). For the compounds with native PTZ unit, like **NI-PTZ-F**, there are broad, structureless fluorescence bands centered at ca. 620 nm in cyclohexane (CHX) and HEX, which are attributed to the CS state emission. Moreover, the fluorescence of the compounds is strongly quenched and red-shifted in polar solvents, as compared to that in CHX and HEX. The higher polarity solvents can stabilize the CS state, and the energy of CS state will decrease. As a result, the emission band will be red-shifted and the fluorescence intensity will be greatly reduced. The results infer that the emissive S_1_ state has a substantial CS character [56]. Thus, the assignment of the emissive S_1_ state to a CS state is reasonable.

Upon oxidation of the PTZ moiety, the degree of fluorescence quenching in polar solvents became less significant than that of the compounds with a native PTZ unit (Figure 2e and 2f) [57]. Moreover, it is noted that the CS emission wavelength of **NI-PTZ-F-O** and **NI-PTZ-Ph-O** is different from a 4-diphenylamino-substituted NI [58]. These results indicate that the ^1^CS state of these two dyads involves the oxidized PTZ unit as a whole electron donor, not only the N atom of the PTZ unit [59–60]. Further, the **NI-PTZ-F-O** and **NI-PTZ-Ph-O** compounds adopt an orthogonal geometry, and the N atom in the PTZ unit is not in π-conjugation with NI moiety due to a conformational restriction [55]. Thus, it is not the ordinary intramolecular charge transfer (ICT) state that affects the fluorescence of these two dyads. Indeed, the highly solvent polarity dependent fluorescence emission intensity, and wavelength are different from the 4-amino NI derivatives, which are much less solvent polarity-dependent [59,61].

**Figure 3:**
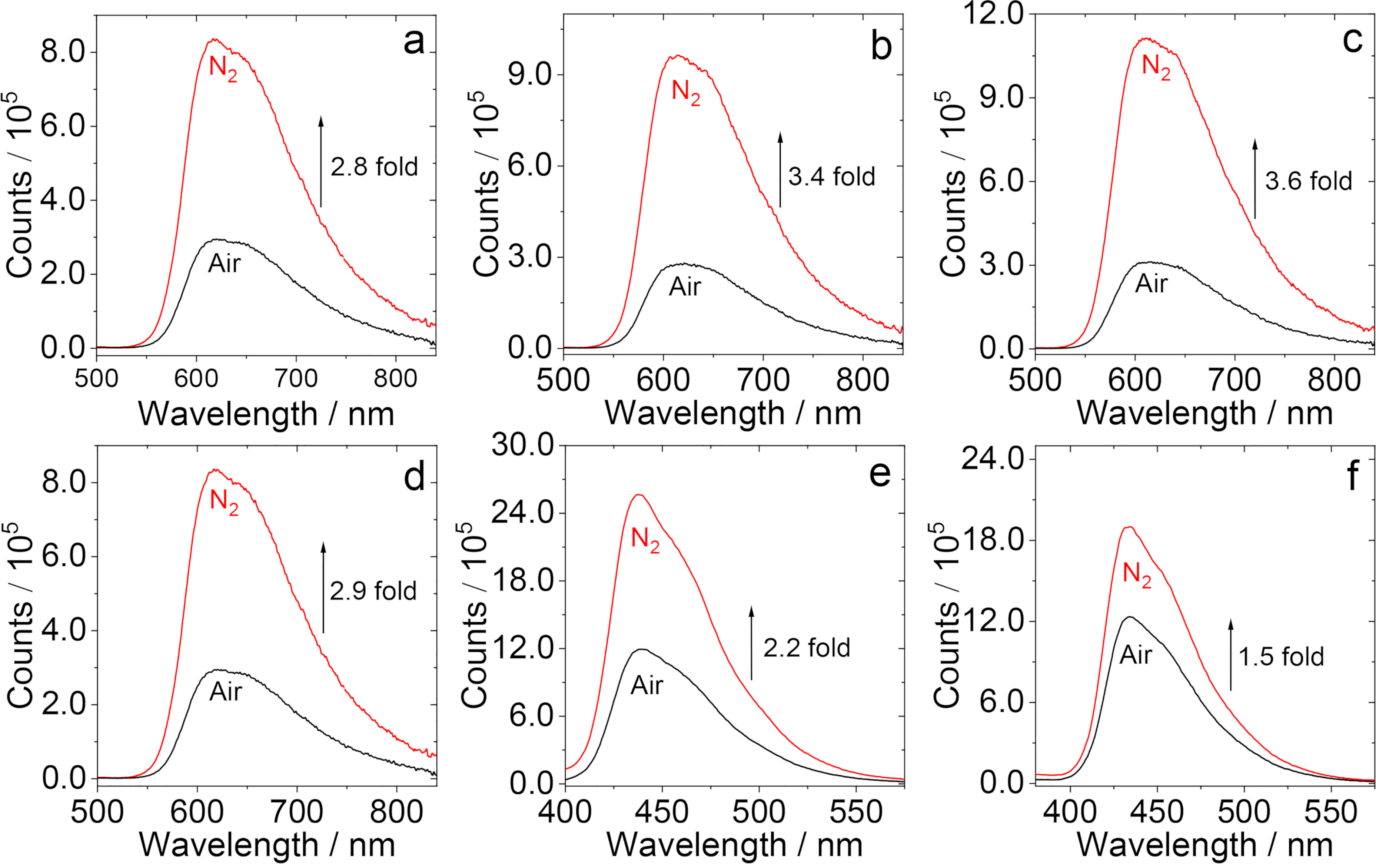
Fluorescence spectra of the dyads. (a) **NI-PTZ-F**, (b) **NI-PTZ-Ph**, (c) **NI-PTZ-CH_3_**, (d) **NI-PTZ-OCH_3_**, (e) **NI-PTZ-F-O**, and (f) **NI-PTZ-Ph-O** in HEX under different atmospheres (N_2_, air). Optically-matched solutions were used, *A* = 0.107, λ_ex_ = 310 nm, 20 °C.

Since a TADF molecule is characterized by a stronger fluorescence under nitrogen atmosphere and a substantial quenching under air or oxygen atmosphere [22], the fluorescence spectra of the dyads containing native PTZ unit under different atmospheres were studied (Figure 3a–d and Figure S32a in Supporting Information File 1). The results show that the fluorescence intensity of the dyads is stronger under a N_2_ atmosphere, but is significantly quenched under air atmosphere. The fluorescence intensity under N_2_ atmosphere is 2–4 times higher than that under an air atmosphere. However, for the dyads with an oxidized PTZ unit (Figure 3e and 3f and Figure S32b in Supporting Information File 1), the fluorescence intensity is less dependent on the atmosphere. Moreover, we suggest that the reduced luminescence of the oxidized molecules in air may be caused by the quenching effect of oxygen to the S_1_ state. According to this experimental phenomenon, we preliminarily speculate that the dyads with a native PTZ unit may have TADF property, but not for the oxidized molecules.

**Figure 4:**
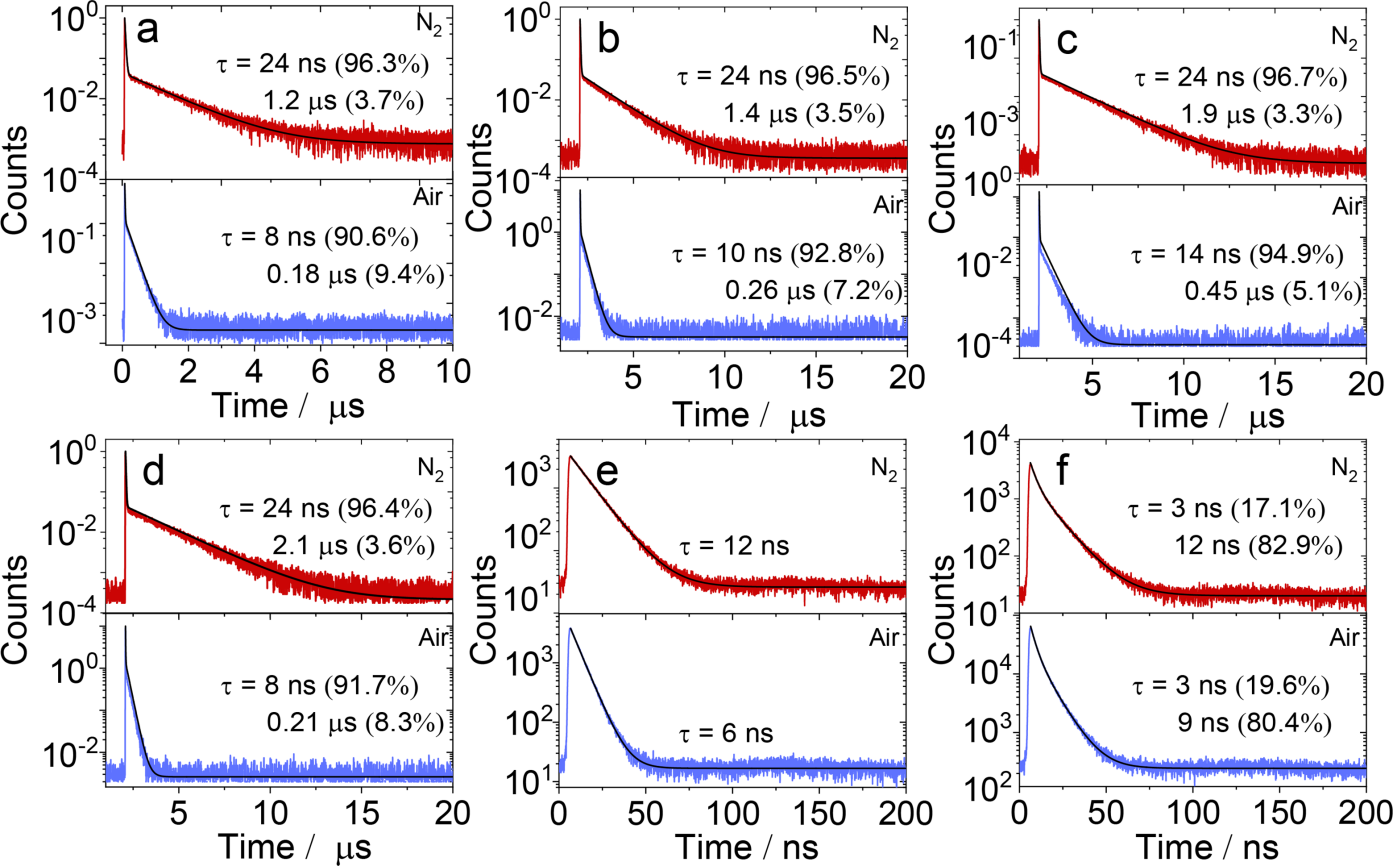
Fluorescence lifetime of (a) **NI-PTZ-F**; (b) **NI-PTZ-Ph**; (c) **NI-PTZ-CH_3_**; (d) **NI-PTZ-OCH_3_** (λ_em_ = 610 nm, *c* = 1.0 × 10^−5^ M) and (e) **NI-PTZ-F-O**; (f) **NI-PTZ-Ph-O** (λ_em_ = 440 nm, *c* = 2.0 × 10^−5^ M) in different atmospheres (N_2_, air). Excited with a picosecond pulsed laser (λ_ex_ = 340 nm), in HEX, 20 °C.

To confirm the above speculation, the fluorescence decay traces of the dyads were studied in detail (Figure 4 and Figure S33 in Supporting Information File 1). The fluorescence decay traces of the dyads containing native PTZ units in HEX show an obvious double exponential feature. For example, the lifetime of **NI-PTZ-F** is 24 ns (96.3%)/1.2 µs (3.7%) under N_2_ atmosphere (Figure 4a). The short-lived component can be attributed to the prompt fluorescence of ^1^CS → S_0_, while the long-lived component is the delayed fluorescence, via the rISC process. In addition, the lifetime of the long-lived component of the compound under air atmosphere is greatly shortened, which also verifies that these compounds have TADF properties. Moreover, with our derivatization approach, the geometry and electron-donating ability of the PTZ unit are unchanged, but the electron-accepting ability of the NI part is changed by introducing different aryl substituents at the nitrogen of the NI unit. We found the magnitude of the long-lived components of the TADF emitters is changed (Figure 4a–d). Interestingly, the dyads with the oxidized PTZ unit do not have long-lived components (Figure 4e and 4f), and their fluorescence lifetimes are basically the same in nitrogen and air atmosphere and the lifetimes are on a nanosecond scale, indicating that the oxidized molecules do not have TADF properties. We reported NI-PTZ analogous dyads recently, however, the delayed fluorescence lifetimes were much longer (2.0–14.4 μs) as compared to the current dyads [47]. TADF emitters with shorter delayed fluorescence lifetimes are suitable for fabrication of OLED devices, to suppress the efficiency roll-off effect.

The photophysical data of all compounds are compiled in Table 1. The fluorescence quantum yields (Φ_F_) of the compounds were measured with an optical integration sphere and were in the range of 2–4% for the dyads with PTZ unoxidized and oxidized. It is interesting to note that the fluorescence quantum yields of the dyads are low, and similar to the native NI (Φ_F_ = 0.5% in HEX), but much lower than for 4-diphenylamino-substituted NI (Φ_F_ = 75% in HEX) [58]. We propose that there is an efficient non-radiative decay channel for the S_1_ state of these dyads, especially for the dyads with the oxidized PTZ unit. ISC is one possible relaxation pathway, because the orthogonal geometry of the dyads is beneficial for SOCT-ISC. Previously we have shown that SOCT-ISC occurs for the analogue of **NI-PTZ-C_5_** [39].

**Table 1:** Photophysical parameters of the compounds.

Compounds	λ_abs_(nm)^a^	ε^b^	λ_em_(nm)^c^	τ_F_(ns)^d^	Φ_F_ (%)^e^	*k*_r_^f^	*k*_nr_^g^

**NI-PTZ-F**	333	2.1	620	8 (90.6%) 180 (9.4%)	3.5	3.9 0.01	1.2 0.06
**NI-PTZ-Ph**	330	2.1	619	10 (92.8%) 260 (7.2%)	3.5	3.2 0.01	1.0 0.04
**NI-PTZ-CH** **_3_**	329	2.0	611	14 (94.9%) 450 (5.1%)	3.5	2.4 0.0	0.7 0.02
**NI-PTZ-OCH** **_3_**	330	2.1	611	8 (91.7%) 210 (8.3%)	4.1	4.7 0.02	1.2 0.05
**NI-PTZ-C** **_5_**	332	1.8	611	9 (94.4%) 240 (5.6%)	2.6	2.7 0.01	1.1 0.04
**NI-PTZ-F-O**	329	2.0	440	6	3.5	5.8	1.6
**NI-PTZ-Ph-O**	330	2.1	434	3 (19.6%) 9 (80.4%)	4.1	2.7 3.7	3.3 1.1
**NI-PTZ-C** **_5_** **-O**	332	1.8	433	6	2.6	4.3	1.6

^a^Maximal UV–vis absorption wavelength in HEX, *c* =1.0 × 10^−5^ M, 20 °C; ^b^Molar absorption coefficient at absorption maxima in HEX, ε: 10^4^ M^−1^ cm^−1^; ^c^emission wavelength in HEX; ^d^fluorescence lifetime under air atmosphere in HEX, λ_ex_ = 340 nm; ^e^fluorescence quantum yields determined in HEX, λ_ex_ = 310 nm; ^f^radiative decay rate constant. *k*_r_ = Φ_F_/τ_F_, in 10^6^ s^−1^; ^g^non-radiative decay rate constant. *k*_r_ = (1–Φ_F_)/τ_F_, in 10^8^ s^−1^.

In order to study the ISC efficiency of the dyads, we measured the singlet oxygen quantum yield (Φ_Δ_) of the dyads in solvents with varying polarity (Table 2). For the dyads containing unoxidized PTZ, the Φ_Δ_ is less than 20% in non-polar solvents, and Φ_Δ_ becomes negligible in polar solvents. It should be noted that given the lowest transient species was not a ^3^LE state, instead a CS state, no formation of ^1^O_2_ should be observed (see below Figure 8 and Figures S36 and S37 in Supporting Information File 1). For the dyads with the oxidized PTZ unit, however, the Φ_Δ_ are much higher, especially in polar solvents. These results indicate that the lowest state of these dyads is the ^3^LE state, and the ISC yields are close to unit (see below Figure 9 and Figure S38 in Supporting Information File 1). However, these results do not necessarily mean that the ISC of the dyads containing a native PTZ unit is poor, it can be that the lowest transient state of these dyads is a CS state. We confirmed this is the case for the dyads of **NI-PTZ-F**, etc.

**Table 2:** Singlet oxygen quantum yields (Φ_Δ_, in %) in different solvents.^a^

Compounds	CHX	HEX	TOL	DCM	ACN

**NI-PTZ-F**	12	18	0	–^b^	–^b^
**NI-PTZ-Ph**	14	18	1	–^b^	–^b^
**NI-PTZ-CH** **_3_**	15	17	0	–^b^	–^b^
**NI-PTZ-OCH** **_3_**	14	23	2	–^b^	–^b^
**NI-PTZ-C** **_5_**	17	19	1	–^b^	–^b^
**NI-PTZ-F-O**	25	30	50	100	100
**NI-PTZ-Ph-O**	23	26	46	100	90
**NI-PTZ-C** **_5_** **-O**	28	36	48	100	96

^a^The *E*_T_ (30) values of the solvents are 30.9 (CHX), 31.0 (HEX), 33.9 (TOL), 40.7 (DCM), and 45.6 (ACN), respectively, in kcal mol^−1^. Singlet oxygen quantum yield (Φ_Δ_) with Ru(bpy)_3_[PF_6_]_2_ as standard (Φ_Δ_ = 0.57 in DCM) in different solvents; λ_ex_ = 340 nm; ^b^not observed.

### Electrochemistry study

**Figure 5:**
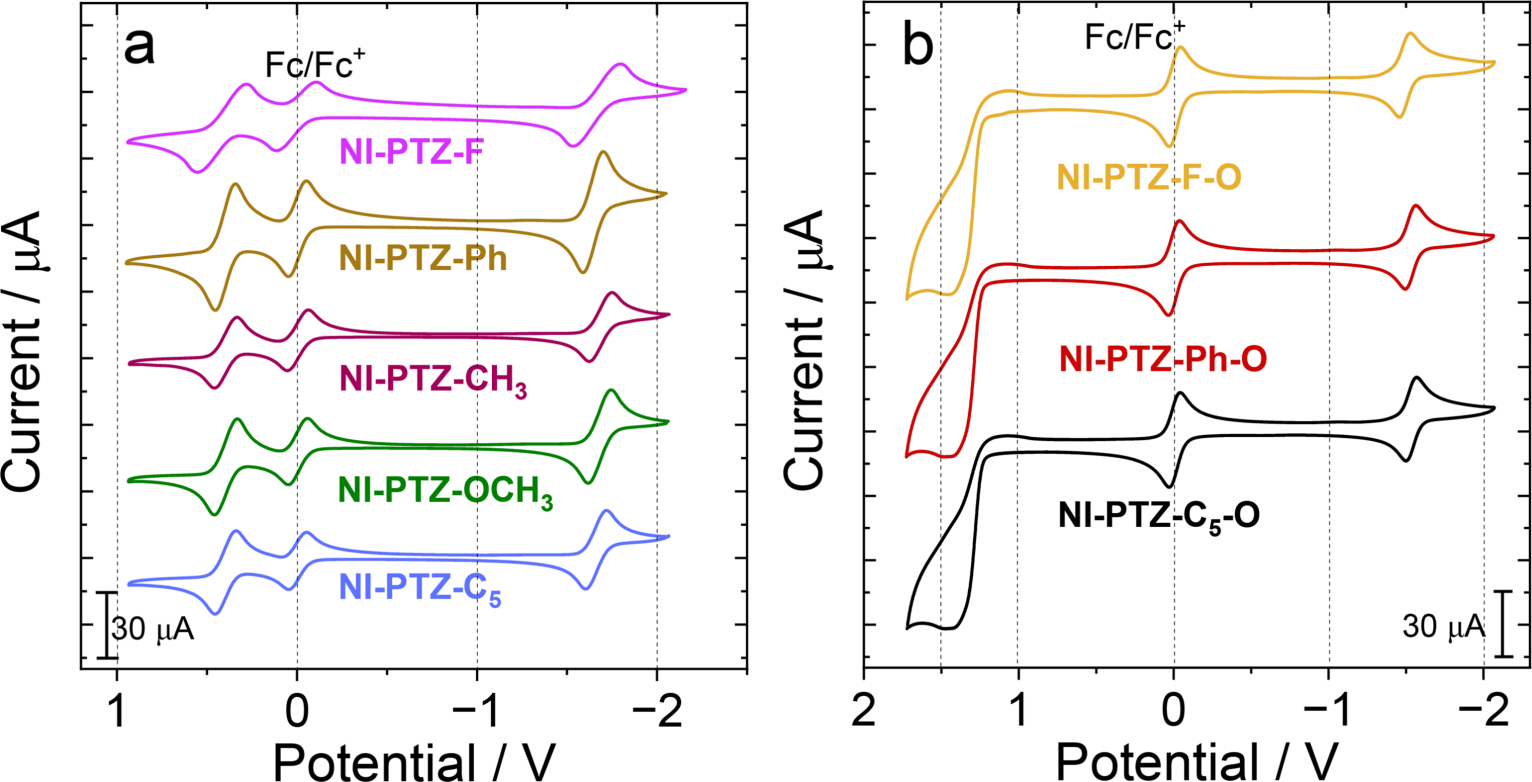
Cyclic voltammograms of the compounds. (a) **NI-PTZ-F**; **NI-PTZ-Ph**; **NI-PTZ-CH_3_**; **NI-PTZ-OCH_3_**; **NI-PTZ-C_5_** in deaerated DCM and (b) **NI-PTZ-F-O**; **NI-PTZ-Ph-O**; **NI-PTZ-C_5_-O** in deaerated ACN. Ferrocene (Fc) was used as internal reference (set as 0 V in the cyclic voltammograms), 0.10 M Bu_4_NPF_6_ as supporting electrolyte, scan rates: 100 mV/s, *c* = 1.0 × 10^−3^ M, 20 °C.

In order to obtain the energy of the CS state, the electrochemistry of these compounds was studied (Figure 5). A reversible oxidation wave at +0.29 V (vs Fc/Fc^+^) was observed for **NI-PTZ-F**, which is attributed to the oxidation of the PTZ part. Moreover, a reversible reduction wave was observed at −1.78 V (vs Fc/Fc^+^) due to the reduction of the NI unit. For all the dyads containing a native PTZ unit, the oxidation potentials are virtually the same. However, the reduction potential changes to some extent, which is consistent with our molecular design to keep the donor part unchanged and modify the NI part by introducing different electron-pushing and withdrawing aryl substituents at the nitrogen atom of the NI unit. For **NI-PTZ-F-O**, an irreversible oxidation wave at +1.38 V (vs Fc/Fc^+^) was observed, which may due to the weak electron-donating ability of the PTZ part after oxidation, and there was still a reversible reduction potential at −1.53 V (vs Fc/Fc^+^). Slightly cathodically shifted reduction waves were observed for other dyads containing an oxidized PTZ unit, i.e., **NI-PTZ-Ph-O** and **NI-PTZ-C_5_-O**.

The redox potentials of the compounds are collected in Table 3. The Gibbs free energy changes (Δ*G*_CS_) for charge separation (CS) as well as the energy of the charge transfer states (*E*_CS_) were calculated using the Rehm–Weller equation (Equations 1–3) and the obtained values are listed in Table 4 [62–64].


[1] $$\Delta G_{\text{CS}}=e\left(E_{\text{OX}}-E_{\text{RED}}\right)-E_{00}+\Delta G_{\text{S}}$$

[2] $$\Delta G_{\text{S}}=\frac{e^{2}}{4\pi \varepsilon_{\text{S}}\varepsilon_{0}R_{\text{CC}}}-\frac{e^{2}}{8\pi \varepsilon_{0}}\left(\frac{1}{R_{\text{D}}}+\frac{1}{R_{\text{A}}}\right)\left(\frac{1}{\varepsilon_{\text{REF}}}-\frac{1}{\varepsilon_{\text{S}}}\right)$$

[3] $$E_{\text{CS}}=e\left[E_{\text{OX}}-E_{\text{RED}}\right]+\Delta G_{\text{S}}$$

**Table 3:** Electrochemical redox potentials of the compounds.^a^

Compounds	*E*_(OX)_/V	*E*_(RED)_/V

**NI-PTZ-F** ^b^	+0.29	−1.78
**NI-PTZ-Ph** ^b^	+0.35	−1.69
**NI-PTZ-CH** **_3_** ^b^	+0.33	−1.75
**NI-PTZ-OCH** **_3_** ^b^	+0.33	−1.74
**NI-PTZ-C** **_5_** ^b^	+0.34	−1.72
**NI-PTZ-F-O** ^c^	+1.38	−1.53
**NI-PTZ-Ph-O** ^c^	+1.38	−1.56
**NI-PTZ-C** **_5_** **-O** ^c^	+1.37	−1.56

^a^Cyclic voltammetry in N_2_-saturated solvents containing 0.10 M Bu_4_NPF_6_, a Pt electrode as the counter electrode, glassy carbon electrode as the working electrode, ferrocene (Fc/Fc^+^) as the internal reference (set as 0 V in the cyclic voltammograms), and Ag/AgNO_3_ couple as the reference electrode; ^b^measured in DCM; ^c^measured in ACN.

**Table 4:** Gibbs free energy changes of the charge separation (Δ*G*_CS_) and energy of charge separation states (*E*_CS_) of the compounds in different solvents.^a^

Compounds	Δ*G*_CS_ (eV)				*E*_CS_ (eV)			

	HEX	TOL	DCM	ACN	HEX	TOL	DCM	ACN

**NI-PTZ-F** ^b^	−0.23	−0.39	−0.83	−0.95	2.38	2.22	1.78	1.66
**NI-PTZ-Ph** ^c^	−0.26	−0.42	−0.86	−0.98	2.35	2.19	1.75	1.63
**NI-PTZ-CH** **_3_** ^d^	−0.29	−0.40	−0.84	−0.96	2.34	2.23	1.79	1.67
**NI-PTZ-OCH** **_3_** ^e^	−0.25	−0.41	−0.85	−0.97	2.37	2.21	1.78	1.66
**NI-PTZ-C** **_5_** ^f^	−0.26	−0.41	−0.86	−0.98	2.37	2.21	1.77	1.65
**NI-PTZ-F-O** ^g^	−0.02	−0.18	−0.63	−0.75	3.22	3.06	2.62	2.49
**NI-PTZ-Ph-O** ^h^	−0.01	−0.17	−0.62	−0.74	3.25	3.06	2.65	2.52
**NI-PTZ-C** **_5_** **-O** ^i^	−0.02	−0.18	−0.63	−0.75	3.24	3.08	2.64	2.51

^a^Cyclic voltammetry in deaerated solutions containing 0.10 M Bu_4_NPF_6_, a Pt electrode as counter electrode, a glassy carbon electrode as working electrode, and Ag/AgNO_3_ couple as the reference electrode; ^b^*E*_00_ = 2.61 eV; ^c^*E*_00_ = 2.61 eV; ^d^*E*_00_ = 2.63 eV; ^e^*E*_00_ = 2.63 eV; ^f^*E*_00_ = 2.62 eV; ^g^*E*_00_ = 3.24 eV; ^h^*E*_00_ = 3.26 eV; ^i^*E*_00_ = 3.26 eV. *E*_00_ (*E*_00_ = 1240/λ) is the singlet state energy of the compounds, λ is the wavelength of the crossing point of normalized UV–vis absorption spectra and fluorescence emission spectra.

It should be noted that the *E*_00_ values used in the analysis is the S_1_ state energy, not the T_1_ energy, because we have shown that the CS is fast in this kind of compact dyads, and the precursor of the CS is the ^1^LE state [58]. From Table 4, it is found that the Δ*G*_CS_ of these compounds are negative in all solvents, indicating that the charge separation process is thermodynamically allowed in these solvents and the CS state energy of the dyads calculated by the redox potentials are slightly higher than the experimental values in HEX (the fluorescence, Figure 2). For the unoxidized molecules, with increasing solvent polarity, the CS state energy decreases sharply. From HEX to ACN, the CS state energy level decreases by ca. 0.7 eV. Note, we did not determine the electron exchange energy (*J*) concerning the unpaired electrons in the CS state (the energy gap of the ^1^CS/^3^CS states is 2*J*). However, since the dyads have orthogonal geometry, the energy gap of the ^1^CS/^3^CS states should be small, on a <0.1 eV scale [39,58]. For the dyads with oxidized PTZ moieties, the CS state energy is higher than that of the unoxidized molecules, and it is also decreased with increasing solvent polarity.

The T_1_ state of NI was determined as 2.25 eV (phosphorescence method) [65], and the T_1_ state of the PTZ unit was determined as 2.45 eV (phosphorescence method) [66]. These energy levels are close to the CS states (Table 4), and thus, in HEX and TOL, the three states ^1^CS, ^3^CS, and ^3^LE share a similar energy, and TADF may be resulted. For the dyads with the oxidized PTZ unit, the *E*_CS_ state of the **NI-PTZ-F-O**, **NI-PTZ-Ph-O**, and **NI-PTZ-C_5_-O** was increased by ca. 0.8 eV. Thus, we do not expect TADF for these three dyads, especially in HEX. In polar solvents, such as ACN, no TADF was observed.

### Thermal stability analysis (thermogravimetry analysis, TGA)

**Figure 6:**
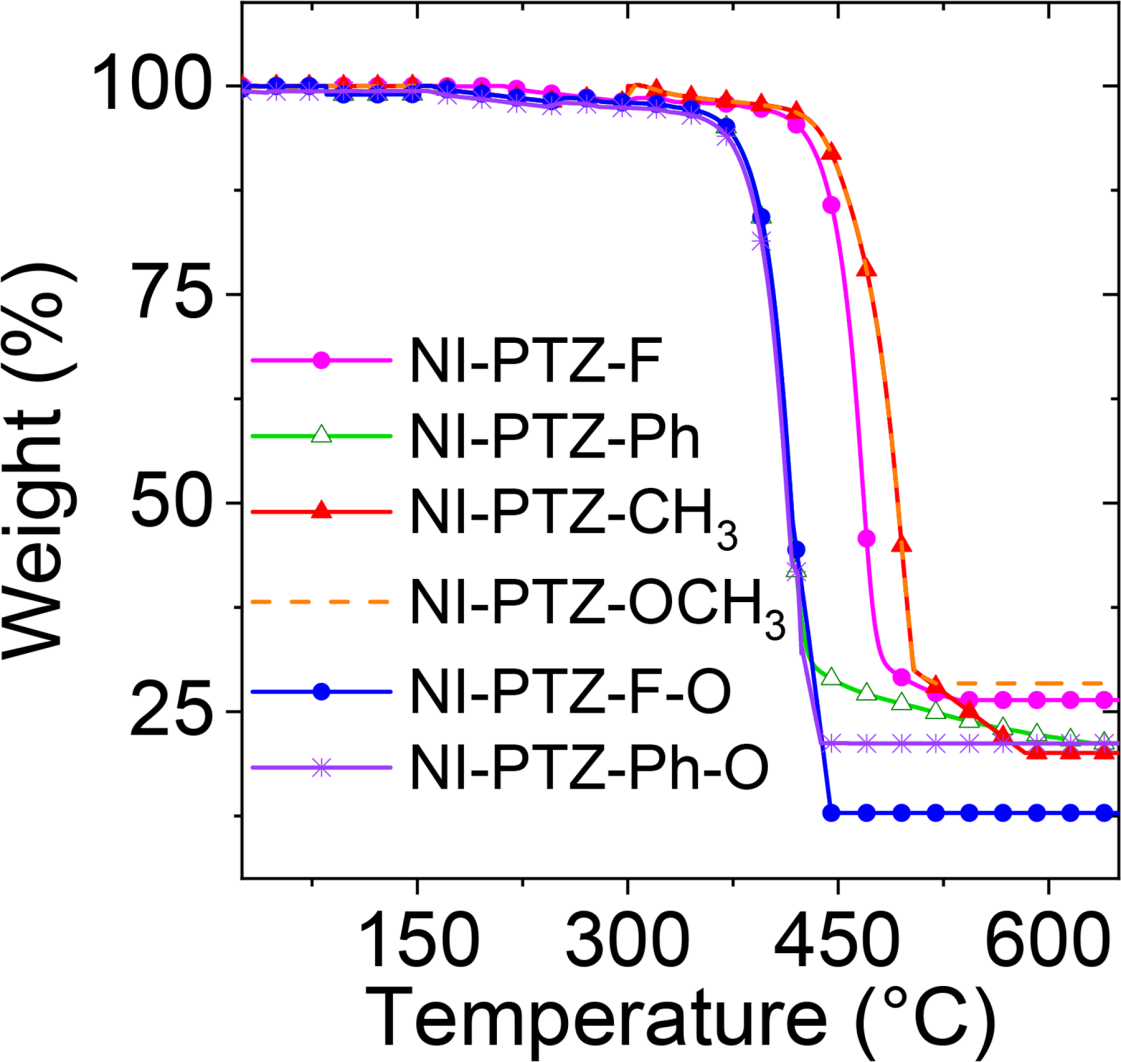
Thermogravimetric analysis curves of **NI-PTZ-F**, **NI-PTZ-Ph**, **NI-PTZ-CH_3_**, **NI-PTZ-OCH_3_**, **NI-PTZ-F-O**, and **NI-PTZ-Ph-O**. Temperature range: 25–800 °C, heating rate: 10 °C/min in N_2_ atmosphere.

The thermal stability of TADF emitters is very important for the application in OLED devices. Therefore, the compounds were investigated by thermogravimetric analysis (TGA) and the results are shown in Figure 6 and Figure S34 in Supporting Information File 1. The thermal decomposition temperature (temperature at 5% thermal weight loss) of each compound were determined as follows: **NI-PTZ-F** (422 °C), **NI-PTZ-Ph** (375 °C), **NI-PTZ-CH_3_** (436 °C), **NI-PTZ-OCH_3_** (432 °C), **NI-PTZ-F-O** (371 °C), **NI-PTZ-Ph-O** (362 °C), **NI-PTZ-C_5_** (436 °C), **NI-PTZ-C_5_-O** (373 °C). The results show that the thermal stability of these compounds is excellent, and the thermal stability of dyads containing unoxidized PTZ units is better than those containing oxidized PTZ units.

### Femtosecond transient absorption (fs-TA) spectra

**Figure 7:**
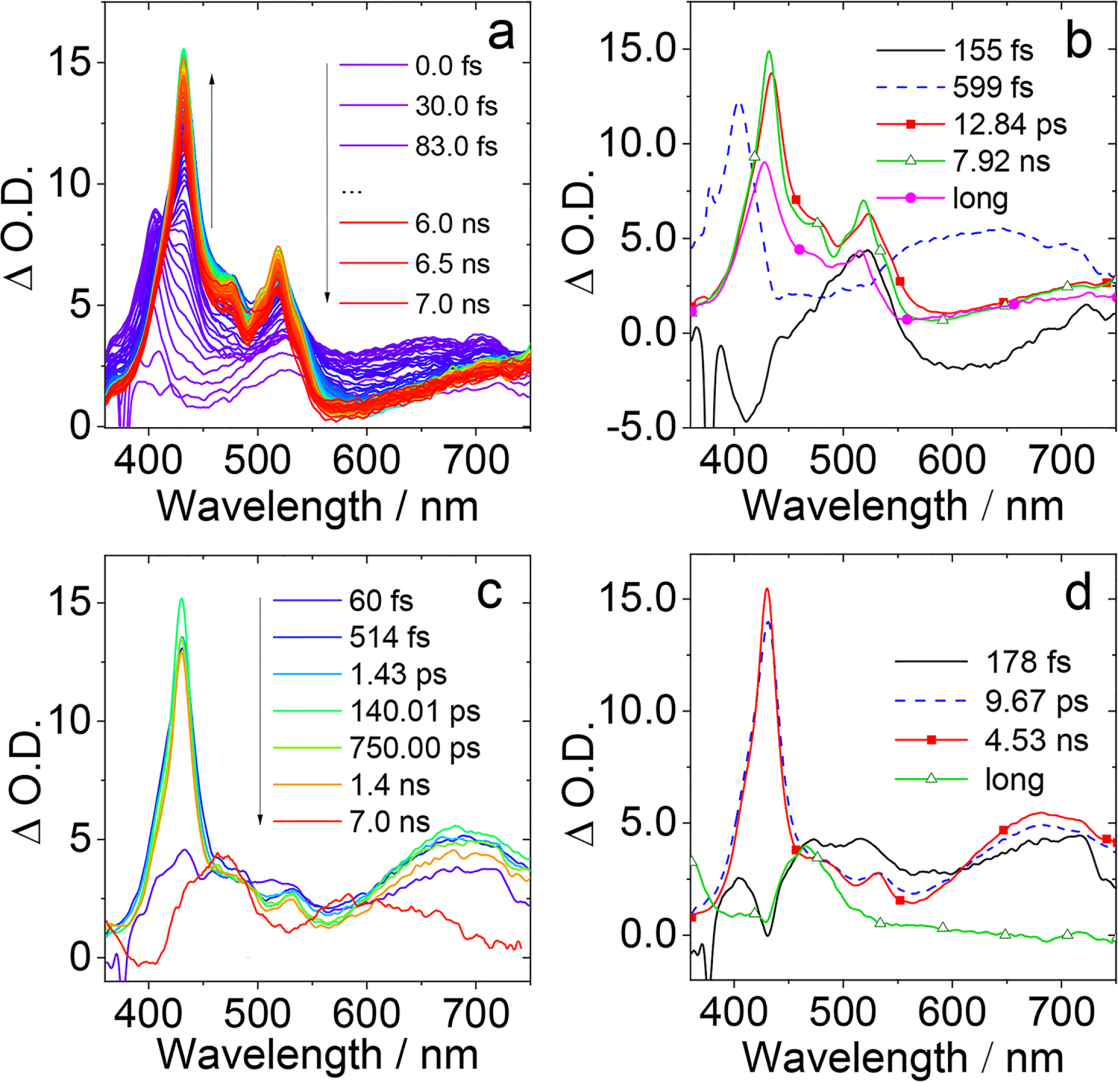
Femtosecond transient absorption spectra of **NI-PTZ-F**. (a) Transient absorption spectra and (b) the EADS obtained with global analysis in HEX. Femtosecond transient absorption spectra of **NI-PTZ-F-O**. (c) Transient absorption spectra and (d) relative EADS obtained with global analysis in ACN. λ_ex_ = 340 nm.

**Figure 8:**
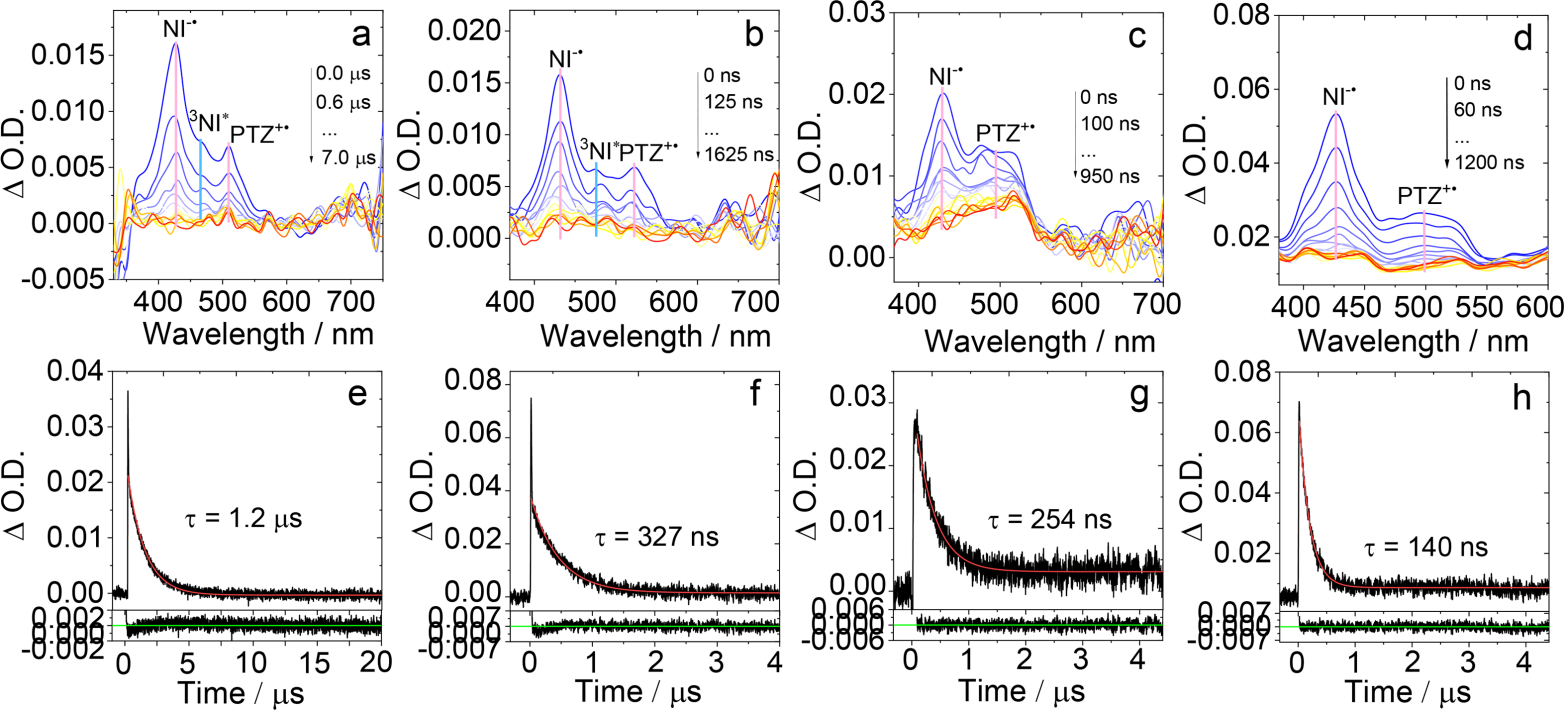
Nanosecond transient absorption spectra of **NI-PTZ-F** in deaerated solvents of (a) HEX (*c* = 2.0 × 10^−5^ M), (b) TOL (*c* = 2.0 × 10^−5^ M), (c) DCM (*c* = 1.0 × 10^−4^ M), and (d) ACN (*c* = 1.0 × 10^−4^ M). The corresponding decay traces are (e) HEX (*c* = 2.0 × 10^−5^ M), (f) TOL (*c* = 2.0 × 10^−5^ M), (g) DCM (*c* = 1.0 × 10^−4^ M), and (h) ACN (*c* = 1.0 × 10^−4^ M) at 430 nm. λ_ex_ = 355 nm, 20 °C.

In order to study the CS, charge recombination (CR), and ISC of the compounds, the femtosecond transient spectra were studied (Figure 7). Taking the **NI-PTZ-F** as an example, the transient absorption spectra measured in HEX and the evolution associated difference spectra (EADS), obtained by global fitting based on sequential kinetic model, are presented (Figure 7a and 7b). The first transient species (black line in Figure 7b) is attributed to a localized singlet excited state of ^1^NI* by comparison with the reported spectra of NI [58]. Within 155 fs, the system evolves to the second spectrum (blue line), for which both a sharp peak centered at 404 nm and a broad band in the range 600–750 nm were observed, which is identified as a relaxation of the ^1^NI* state and the evolution towards a CS state. Then, within about 599 fs, the system evolves to the third spectrum, which shows a sharp absorption band centered at 433 nm and 522 nm, corresponding to the absorption of NI^−•^ and PTZ^+•^, thus being a CS state. Then, within 12.84 ps, another spectrum is developed, which has still the characteristics of the CS state, however, the intensity of the two bands increases and both peaks blue-shift by about 3 nm, indicating a vibrational relaxation of the CS state and thus, we propose the geometry and solvent relaxation takes ca. 12.84 ps. At the same time, in this process, the triplet features of ^3^LE start to appear (460 nm). Since the relaxed CS state and CR evolve within 7.92 ns, this is in agreement with the TADF luminescence studies (ca. 8 ns, Figure 4a), thus the decay of the ^1^CS state in aerated solution takes ca. 7.92 ns. Then, the system evolves to the final spectrum (magenta), which is characterized by the features of ^3^LE and CS states, and it is in good agreement with the ns-TA results (see below Figure 8a). Moreover, we propose that the precursor of the ^3^CS state is the ^3^LE state. While in ACN (Figure S35a and S35b in Supporting Information File 1), the fluorescence lifetime is not observed, we speculate that the lifetime of ^1^CS state is too short, and finally within 8.08 ps, a ^3^CS state has been formed, with some characteristic absorption peaks of NI^−•^ (430 nm) and PTZ^+•^ (502 nm). Although we reported **NI-PTZ** analogous dyads recently, the fs-TA spectroscopy was not used to study their photophysical processes [47].

Femtosecond transient spectra of **NI-PTZ-F-O** were also studied (Figure 7c and 7d). The solubility of **NI-PTZ-F-O** in HEX is poor, which leads to a poor transient spectral signal (Figure S35c and S35d in Supporting Information File 1) and we did not obtain the EADS, CS, CR, and other time constants exactly. In ACN, the first transient species (black line in Figure 7d) can be recognized as a localized singlet excited state of ^1^NI* by comparison with the spectra reported for the parent NI [58]. Within about 178 fs, the blue line, which has two sharp peaks at 430 nm and 530 nm and a wide peak at 600–750 nm, indicates the beginning of ^1^CS state formation. Then, within 9.67 ps, there is a vibrational relaxation of the charge separated (CS) state (red line in Figure 7d). Later in about 4.53 ns, the triplet features of ^3^LE appear with a characteristic absorption peak of ^3^NI* at 463 nm and this process can be ascribed to SOCT-ISC (green line in Figure 7d). This postulation is supported by the nanosecond transient spectra (see below).

### Nanosecond transient absorption (ns-TA) spectra

In order to study the lowest-lying transient species of the dyads formed upon photoexcitation, the ns-TA spectra of **NI-PTZ-F** in HEX were studied (Figure 8a). Upon pulsed laser excitation, a positive absorption band centered at ca. 430 nm was observed, which is inconsistent with the characteristic triplet state absorption of the NI unit (460 nm) [39], while this positive absorption band is closer to that of NI^−•^, which was determined at ca. 430 nm [58]. Therefore, this absorption band is assigned to the NI radical anion. The excited state absorption (ESA) band centered at 460 nm is attributed to the ^3^NI state. Moreover, another positive absorption band centered at ca. 510 nm is attributed to absorption of PTZ^+•^ [39]. Therefore, we propose that the lowest T_1_ state of **NI-PTZ-F** is an admixture of ^3^CS and ^3^LE states. This is in agreement with the fs-TA spectral studies (Figure 7a and 7b). Similar ns-TA spectra were observed for other dyads without an oxidized PTZ unit (Figure S36 in Supporting Information File 1).

In order to study the solvent-dependency of the dyads containing native PTZ units, the ns-TA spectra of **NI-PTZ-F** in HEX, TOL, DCM, and ACN as solvents were studied (Figure 8). With increasing of the solvent polarity, the CS state energy will decrease, yet the ^3^LE state energy will be intact to a large extent [58]. The results showed that in HEX, both ^3^LE and CS states were observed. Herein, we assign the CS state to ^3^CS, not ^1^CS, based on lifetimes. Note, even if the ^1^CS state (emission state) is populated via rISC, it is unlikely to be detected in ns-TA spectra due to its transient character (the lifetime of this state is short, its concentration is too low to be detected by ns-TA spectroscopy) [67–68]. The monoexponential decay kinetics indicate that the ^3^LE and ^3^CS state are in good equilibrium, i.e., the spin–vibronic coupling, which is critical for the TADF [27–28,31,33,46].

In polar solvents, however, the ^3^NI state signal in the ns-TA spectra diminished, but the NI^−•^ and PTZ^+•^ signals were persistent (Figure 8b, 8c, and 8d). Moreover, the transient species lifetimes decreased to 140 ns in ACN. In polar solvents, the CS state energy becomes lower, therefore, only the ^3^CS state was observed. In this case, we assume the spin–vibronic coupling becomes weaker, accordingly, no TADF is resulted, even when the ^3^CS state is populated. Similar results were found for other unoxidized compounds (Figure S37 in Supporting Information File 1). A theoretical model predicts that without the spin–vibronic coupling, the rISC is nonefficient and the TADF will be inhibited [27–28,46]. Therefore, our results represent a solid experimental evidence for the spin–vibronic coupling model of the TADF.

**Figure 9:**
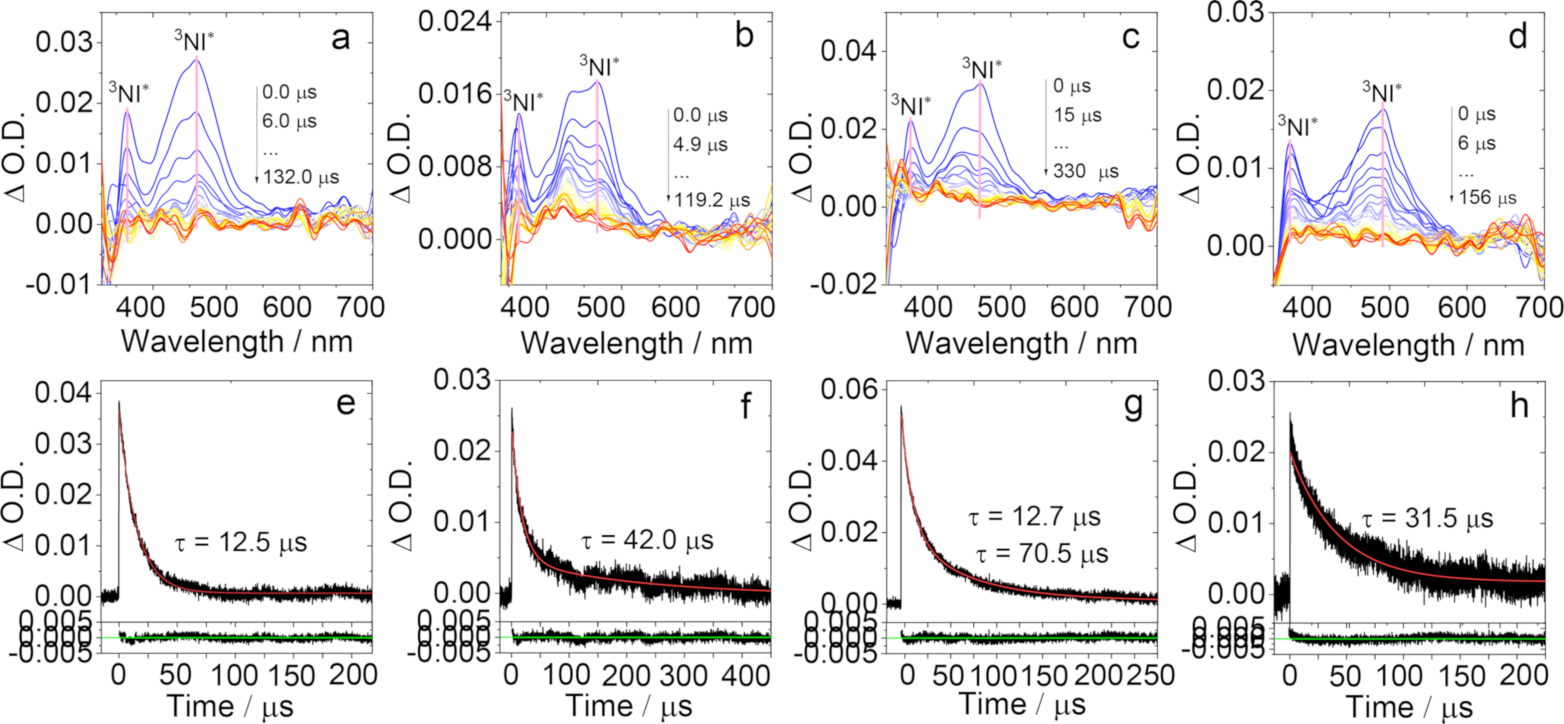
Nanosecond transient absorption spectra of (a) **NI-PTZ-F-O** (*c* = 4.0 × 10^−5^ M), (b) **NI-PTZ-Ph-O** (*c* = 4.0 × 10^−5^ M), (c) **NI-PTZ-C_5_-O** (*c* = 4.0 × 10^−5^ M), and (d) **F-NI-Br** (*c* = 2.0 × 10^−5^ M). The corresponding decay traces are (e) **NI-PTZ-F-O** (*c* = 4.0 × 10^−5^ M), (f) **NI-PTZ-Ph-O** (*c* = 4.0 × 10^−5^ M), (g) **NI-PTZ-C_5_-O** (*c* = 4.0 × 10^−5^ M), and (h) **F-NI-Br** (*c* = 2.0 × 10^−5^ M) in deaerated ACN at 460 nm. λ_ex_ = 355 nm, 20 °C.

The ns-TA spectra of oxidized molecules were also studied (Figure S38 in Supporting Information File 1) in HEX. For **NI-PTZ-F-O**, the ESA bands centered at 360 nm and 460 nm were observed, which is attributed to the ^3^NI state. Moreover, the ESA bands are similar to those observed for **F-NI-Br** and **NI-Br** [39]. Therefore, we propose that the low T_1_ state of **NI-PTZ-F-O** is a ^3^LE state. Similar results were found for other oxidized molecules. With the increase of the solvent polarity, the energy of the CS state decreases. Therefore, the ns-TA spectra of the compounds were studied (Figure 9) in ACN. We found that the transient absorption feature of the dyads with an oxidized PTZ unit does not change, i.e., a ^3^LE state was observed even in ACN. This is supported by the ns-TA spectra of the reference compounds in ACN. Experimentally, no TADF was observed for these dyads. These results confirmed the critical role of the spin–vibronic coupling, i.e., the ^3^LE mediate ^3^CS → ^1^CS rISC in TADF [27]. The photochemical properties of each compound in different solvents are summarized in Table 5.

**Table 5:** Summary of photochemical properties of the compounds.

Compounds	TADF property		The last-observed transient species (fs-TA)		The lowest-lying transient species (ns-TA)	
	
	HEX	ACN	HEX	ACN	HEX	ACN

**NI-PTZ-F**	yes	–^a^	^3^CS, ^3^LE	^3^CS	^3^CS, ^3^LE	^3^CS
**NI-PTZ-Ph**	yes	–^a^	–^b^	–^b^	^3^CS, ^3^LE	^3^CS
**NI-PTZ-CH** **_3_**	yes	–^a^	–^b^	–^b^	^3^CS, ^3^LE	^3^CS
**NI-PTZ-OCH** **_3_**	yes	–^a^	–^b^	–^b^	^3^CS, ^3^LE	^3^CS
**NI-PTZ-C** **_5_**	yes	–^a^	–^b^	–^b^	^3^CS, ^3^LE	^3^CS
**NI-PTZ-F-O**	no	no	–^a^	^3^LE	^3^LE	^3^LE
**NI-PTZ-Ph-O**	no	no	–^b^	–^b^	^3^LE	^3^LE
**NI-PTZ-C** **_5_** **-O**	no	no	–^b^	–^b^	^3^LE	^3^LE

^a^Not observed; ^b^not measured.

### Computational investigations

**Figure 10:**
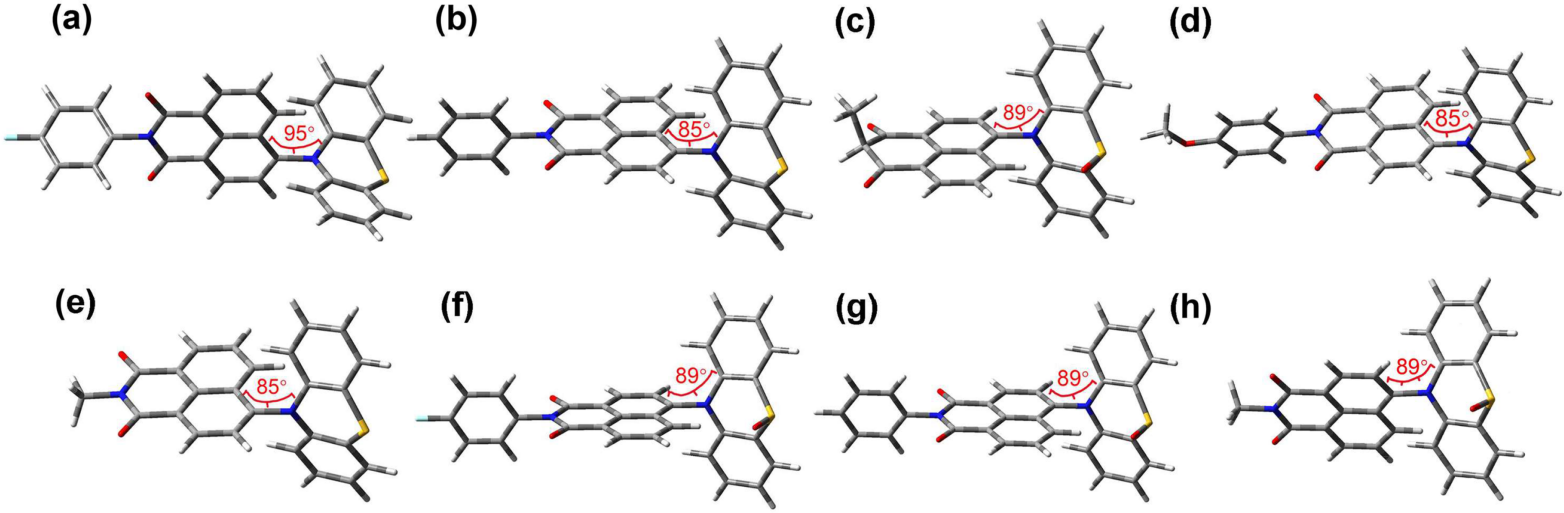
Optimized ground state geometry of (a) **NI-PTZ-F**, (b) **NI-PTZ-Ph**, (c) **NI-PTZ-CH_3_**, (d) **NI-PTZ-OCH_3_**, (e) **NI-PTZ-C_5_**, (f) **NI-PTZ-F-O**, (g) **NI-PTZ-Ph-O**, and (h) **NI-PTZ-C_5_-O**. Calculated at the B3LYP/6-31G(d) level of theory using Gaussian 09.

The optimized ground state geometry of the dyads at the DFT-B3LYP/6-31G (d) level of theory determined with Gaussian 09 shows that the dihedral angles between the PTZ and NI moieties are close to 90°, which is beneficial for SOCT-ISC (Figure 10). Thus, the ^1^LE → ^1^CS → ^3^LE → ^3^CS is facilitated, as well as the rISC processes, i.e., ^3^CS→ ^3^LE → ^1^CS, thus the TADF becomes possible. Moreover, we found that the PTZ unit in the dyads adopts a puckered geometry (i.e., nonplanar geometry). This is known for the PTZ moiety [69–70]. We also optimized the geometry of the dyads at the S_1_ and T_1_ states (Tables S1 and S2 in Supporting Information File 1). In gas phase or nonpolar solvents, the dihedral angles between the NI and the PTZ moiety are in the range of 73–90°, close to that of the S_0_ state. However, the PTZ unit adopts a planar geometry, especially for the S_1_ and T_1_ states optimized in polar solvents. It is known that the PTZ^+•^ adopts a planar geometry [69–70]. These results indicate that the S_1_ and T_1_ states of the dyads containing a native PTZ unit are in the CS state. For the dyads containing an oxidized PTZ moiety, however, the PTZ moiety always adopts a puckered geometry, indicating the S_1_ and T_1_ states of these dyads are LE states, not CS states. These results infer that the T_1_ state of the dyads with the oxidized PTZ is a ^3^LE state, not a ^3^CS state, even in polar solvents, such as ACN. These results are supported by the ns-TA spectral observations.

**Figure 11:**
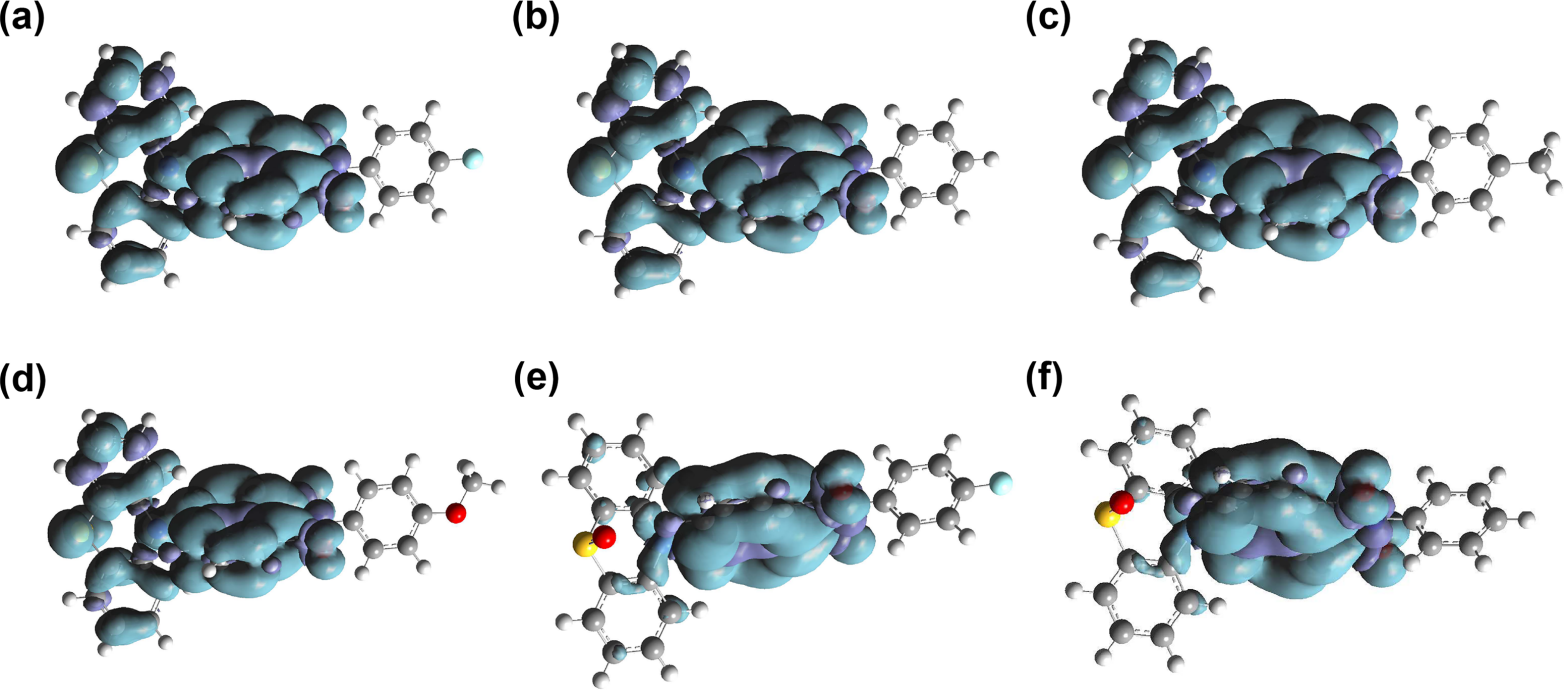
Spin density surfaces of the dyads in the T_1_ state (gas phase) of (a) **NI-PTZ-F**, (b) **NI-PTZ-Ph**, (c) **NI-PTZ-CH_3_**, (d) **NI-PTZ-OCH_3_**, (e) **NI-PTZ-F-O**, and (f) **NI-PTZ-Ph-O**. Calculated at the B3LYP/6-31G(d) level of theory using Gaussian 09. Isovalues = 0.02.

The spin density surface of the T_1_ state of the dyads were also computed (Figure 11 and Table S3 in Supporting Information File 1). For the dyads containing a native PTZ moiety, the T_1_ state density is delocalized on the NI and PTZ moieties. Since these two moieties have different T_1_ state energies (2.25 and 2.45 eV, respectively), the T_1_ state should be a ^3^CS state, not an equilibrium of ^3^PTZ/^3^NI state. In polar solvents (e.g., ACN), literally the same results were observed. These results are in agreement with the ns-TA spectral studies (Figure 8 and Figures S36 and S37 in Supporting Information File 1). For the dyads containing oxidized PTZ moieties, however, the T_1_ state is always confined on the NI moiety. These results infer that the T_1_ state of the dyads with the oxidized PTZ is a ^3^LE state, not a ^3^CS state, even in polar solvents, such as ACN. These results are fully supported by the ns-TA spectral observations (Figure 9 and Figure S38 in Supporting Information File 1).

**Figure 12:**
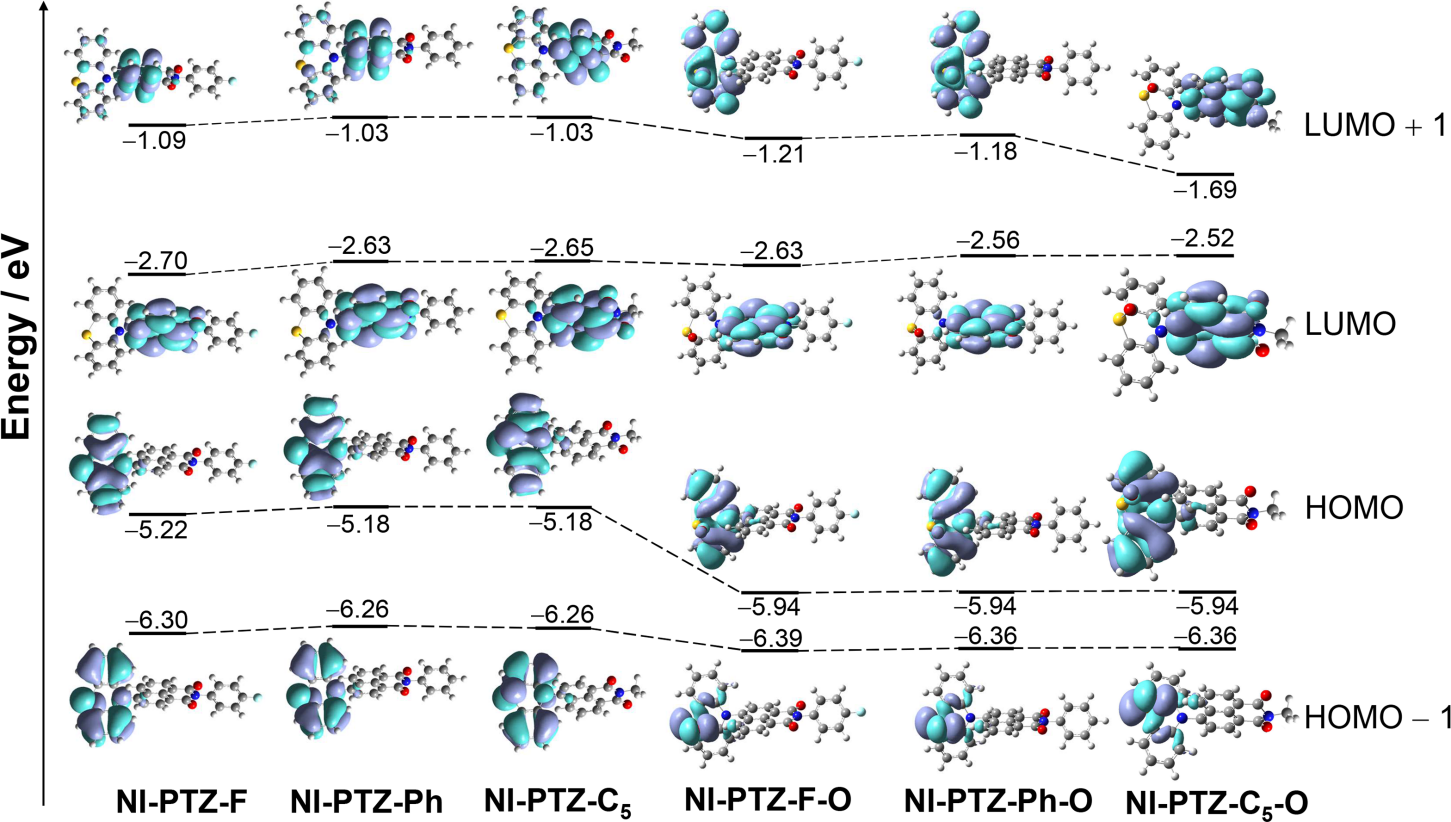
Selected frontier molecular orbitals of **NI-PTZ-F**, **NI-PTZ-Ph**, **NI-PTZ-C_5_**, **NI-PTZ-F-O**, **NI-PTZ-Ph-O**, and **NI-PTZ-C_5_-O** calculated by DFT at the B3LYP/6-31G(d) level of theory using Gaussian 09, based on the optimized ground state geometries, respectively. Isovalues = 0.02.

The frontier molecular orbitals (MOs) were studied (Figure 12 and Figure S39 in Supporting Information File 1). For all the dyads, the HOMOs are confined on the PTZ or the oxidized PTZ moieties, whereas the LUMOs are localized on the NI moieties. Interestingly, for the dyads containing a native PTZ moiety, the HOMO energy is slightly varied by up to 0.04 eV, which is attributed to the electron-withdrawal or donating feature of the aryl substituent attached to the nitrogen atom of the NI moieties. Interestingly, although there is no distribution of LUMO on the aryl moiety, the LUMO energy of the dyads are varied by up to 0.12 eV. These subtle variations of the molecular orbital energy impose effects on the photophysical property of the dyads, such as the delayed fluorescence lifetime of the TADF emissions. Of note, upon oxidation of the PTZ moiety in the dyads, the HOMO energy decreases substantially by up to 0.8 eV, whereas the LUMO energy changed by less than 0.2 eV. Thus, the HOMO/LUMO energy gap increases upon oxidation of the PTZ moiety. This is supported by the blue-shifted fluorescence of the dyads with the oxidized PTZ moieties as compared to the dyads containing the native PTZ moiety (Figure 2). Since the NI moiety is intact, the ^3^CS state becomes much higher than the ^3^LE state and we postulate the TADF will vanish for these dyads. This conclusion is fully supported by the experimental observations. These studies show that our strategy of tuning the energy ordering of the ^1^CS, ^3^LE, and ^3^CS states by changing the electron-donating and withdrawal-capability of the PTZ and NI moieties is successful.

**Scheme 2:**
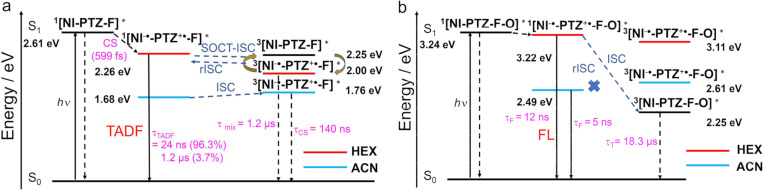
Simplified Jablonski diagram of (a) **NI-PTZ-F** and (b) **NI-PTZ-F-O**. The ^1^LE state (^1^[NI–PTZ–F–O]^*^) energy is derived from the spectroscopic data (the intersection of normalized UV–vis absorption and fluorescence spectra). The triplet state (^3^[NI^−•^–PTZ^+•^–F–O]^*^) energy is computed by the TDDFT method, which was performed at the B3LYP/6-31G(d) level of theory using Gaussian 09W. The ^1^CS state (^1^[NI^−•^–PTZ^+•^–F–O]^*^) energy is obtained from electrochemical data in Table 4 (^1^CS/^3^CS: 2*J* < 0.2 eV).

The photophysical processes of the dyads are summarized in Scheme 2. For **NI-PTZ-F**, the ^1^CS, ^3^CS, and ^3^LE states share similar energies. Thus, TADF was observed. The efficient spin–vibronic coupling of the ^3^CS/^3^LE states was supported by ns-TA spectral studies, in which both the ^3^CS and ^3^LE states were observed, and the two transient species decay with the same kinetics. In polar solvents, however, the CS state energy decreases sharply, yet, the ^3^LE state energy does not change, thus, although a long-lived ^3^CS state was found in ACN (τ_CS_ = 140 ns), no TADF was observed. This is a solid experimental evidence that spin–vibronic coupling is essential for TADF, and the ^3^CS → ^1^CS rISC is slow, without the coupling with the intermediate ^3^LE state. The conventionally used transient luminescence spectral method is unable to supply such in-depth mechanistic insights into the TADF mechanism. For the dyads with the oxidized PTZ units, however, the CS state energy is increased by up to 0.8 eV, yet the ^3^LE state energy does not change. Thus the spin–vibronic coupling between the ^3^LE and ^3^CS is weak and no TADF was observed for this dyad only a long-lived ^3^LE state was observed in the ns-TA spectra. These studies are useful for studying the complex photophysical processes involved in the TADF of electron donor–acceptor dyads, and for molecular structure design of new electron donor–acceptor TADF emitters.

## Conclusion

In summary, we prepared a series of naphthalimide (NI)–phenothiazine (PTZ) electron donor–acceptor dyads, to make an in-depth study of the photophysical mechanism of the thermally activated delayed fluorescence (TADF) of the electron donor–acceptor emitters. In order to tune the photophysical properties, we changed the electron-donating and the electron-withdrawal capability of the PTZ and NI moieties, respectively. From the UV–vis absorption and fluorescence emission spectra, we observed changes of S_0_ → ^1^CS absorption bands, which showed the tuning effect. For the dyads containing a native PTZ unit, TADF was observed, and with changing the aryl substituents at the NI unit, the prompt and delayed fluorescence lifetimes changed as well. In polar solvents, no TADF was observed. Moreover, we did not observe TADF in the dyads with oxidized PTZ units. Femtosecond transient absorption spectra show the charge separation takes ca. 0.6 ps, and the formation of the admixture of ^3^LE and ^1^CS/^3^CS states was observed (in *n*-hexane). The decay of the ^1^CS state and charge recombination (CR) in aerated *n*-hexane takes ca. 7.92 ns. While for the dyads with an oxidized PTZ unit, ^1^CS state formation takes ca. 178 fs and the CR takes 4.53 ns to give the ^3^LE state (in acetonitrile). Nanosecond transient absorption (ns-TA) spectra show that both ^3^CS and ^3^LE states were observed for the scenario where TADF occur, whereas only the ^3^LE state or the ^3^CS state were observed for the systems lacking TADF. This is a rare but unambiguous experimental evidence that the spin–vibronic coupling of ^3^CS/^3^LE states is crucial for TADF. Without the mediating effect of the ^3^LE state, no TADF will be resulted, even if the long-lived ^3^CS state is populated (lifetime τ_CS_ ≈ 140 ns). This experimental result confirms the ^3^CS → ^1^CS reverse intersystem crossing (rISC) is slow, without coupling with an approximate ^3^LE state. Thermogravimetric analysis (TGA) shows that the thermal decomposition temperature of these compounds is higher than 360 °C, and the thermal stability is excellent. From the above studies, TADF properties of such compounds can be studied with conventional spectroscopic methods (the UV–vis absorption and fluorescence emission spectra, etc.), and herein we also demonstrated that transient absorption spectra (femtosecond/nanosecond transient absorption spectra) can be used to study the TADF properties, for instance by monitoring the dark states. In addition, we show that the TADF properties of these dyads can be tuned by changing the electron-donor (**NI-PTZ-F-O**, **NI-PTZ-Ph-O**) or electron-acceptor (**NI-PTZ-F**, **NI-PTZ-Ph**, **NI-PTZ-CH_3_**, **NI-PTZ-OCH_3_**). We found that the energy gaps between the three states (^1^CS, ^3^CS and ^3^LE states), which can be judiciously controlled by molecular design, play an important role in the occurrence of TADF. These studies not only contribute to the in-depth understanding of the photophysical mechanism of TADF emitters, but also provide more molecular systems for the molecular structure design of new electron donor–acceptor TADF emitters.

## Experimental

### General methods

All the chemicals used in synthesis are analytical pure and were used as received without further purification. UV–vis absorption spectra were measured on a UV-2550 Shimadzu spectrophotometer. Fluorescence spectra were recorded with an FS5 spectrofluorometer (Edinburgh instruments Ltd., U.K.). Fluorescence quantum yields (Φ_F_) were measured by an absolute photoluminescence quantum yield spectrometer (Quantaurus-QY Plus C13534-11, Hamamatsu Ltd., Japan). Luminescence lifetimes of compounds were measured with an OB920 luminescence lifetime spectrometer (Edinburgh Instruments Ltd., U.K.). **NI-PTZ-F**, **NI-PTZ-Ph**, **NI-PTZ-CH_3_**, **NI-PTZ-OCH_3_**, and the oxidized molecules **NI-PTZ-F-O** were prepared according to the literature methods [11,39,43,71].

### Synthesis of **NI-PTZ-F**

Compound **NI-PTZ-F** was synthesized by a modified literature method [39]. Under N_2_ atmosphere, **F-NI-Br** (200 mg, 0.542 mmol), phenothiazine (130 mg, 0.650 mmol), Pd(OAc)_2_ (22 mg, 0.098 mmol), and sodium *tert*-butoxide (70 mg, 0.732 mmol) were dissolved in dry toluene (8 mL). Then, tri-*tert*-butylphosphine tetrafluoroborate (19 mg, 0.065 mmol) was added. The mixture was refluxed and stirred for 8 h under N_2_. After cooling, water (20 mL) was added, and the mixture was extracted with ethyl acetate (3 × 30 mL). After separation, the combined organic layer was washed with water and brine (3 × 30 mL), respectively, dried over anhydrous Na_2_SO_4_, and the solvent was evaporated under reduced pressure. The crude product was purified by column chromatography (silica gel, DCM/PE 1:5, v:v). Compound **NI-PTZ-F** was obtained as orange solid. Yield: 40 mg (15%); mp 136.2–137.2 °C; ^1^H NMR (CDCl_3_, 400 MHz) δ 8.87 (d, *J* = 7.63 Hz, 1H), 8.70 (d, *J* = 7.25 Hz, 1H), 8.60 (d, *J* = 8.51 Hz, 1H), 8.00 (d, *J* = 7.63 Hz, 1H), 7.77–7.81 (m, 1H), 7.29–7.35 (m, 4H), 7.11 (d, *J* = 7.51 Hz, 2H), 6.85–6.88 (m, 2H), 6.77–6.80 (m, 2H), 6.08 (d, *J* = 8.13 Hz, 2H); ^13^C NMR (CDCl_3_, 125 MHz) δ 162.9, 158.1, 151.9, 142.6, 138.2, 138.2, 130.8, 130.3, 128.0, 127.6, 124.6, 123.5, 121.1, 117.5, 116.4, 111.8; HRMS–ESI (*m*/*z*): [M + H]^+^ calcd for C_30_H_17_FN_2_O_2_S, 489.0995; found, 489.1072.

### Synthesis of **NI-PTZ-Ph**

**NI-PTZ-Ph** was synthesized by a reported method [11,71] similar to that of **NI-PTZ-F**. The crude product was purified by column chromatography (silica gel, DCM/PE 1:3, v:v). Compound **NI-PTZ-Ph** was obtained as orange solid. Yield: 243 mg (52%); mp 126.2–127.2 °C; ^1^H NMR (CDCl_3_, 400 MHz) δ 8.87 (d, *J* = 7.63 Hz, 1H), 8.70 (d, *J* = 7.13 Hz, 1H), 8.59 (d, *J* = 8.38 Hz, 1H), 7.99 (d, *J* = 7.63 Hz, 1H), 7.76–7.80 (m, 1H), 7.57–7.61 (m, 2H), 7.52 (d, *J* = 7.38 Hz, 1H), 7.35 (d, *J* = 7.25 Hz, 2H), 7.11 (d, *J* = 7.50 Hz, 2H), 6.77–6.87 (m, 4H), 6.09 (d, *J* = 7.76 Hz, 2H); ^13^C NMR (CDCl_3_, 125 MHz) δ 164.1, 163.0, 143.7, 143.4, 138.8, 133.2, 133.0, 131.5, 130.5, 130.3, 130.2, 128.3, 128.0, 124.2, 122.1, 120.6, 115.8; HRMS–ESI (*m*/*z*): [M + H]^+^ calcd for C_30_H_18_N_2_O_2_S, 471.1089; found, 471.1159.

### Synthesis of **NI-PTZ-CH_3_**

**NI-PTZ-CH_3_** was synthesized with a method similar to that of **NI-PTZ-F**. The crude product was purified by column chromatography (silica gel, DCM/PE 1:3, v:v). The product was obtained as orange solid. Yield: 230 mg (80%); mp 100.2–101.5 °C; ^1^H NMR (CDCl_3_, 400 MHz) δ 8.87 (d, *J* = 7.63 Hz, 1H), 8.69 (d, *J* = 8.38 Hz, 1H), 8.58 (d, *J* = 8.50 Hz, 1H), 7.98 (d, *J* = 7.76 Hz, 1H), 7.75–7.79 (m, 1H), 7.38 (d, *J* = 8.01 Hz, 2H), 7.23 (d, *J* = 8.26 Hz, 2H), 7.10 (dd, *J*_1_ = 1.50 Hz, *J*_2_ = 1.50 Hz, 2H), 6.84–6.88 (m, 2H), 6.78–6.80 (m, 2H), 6.08 (dd, *J*_1_ = 1.13 Hz, *J*_2_ = 1.13 Hz, 2H), 2.46 (s, 3H); ^13^C NMR (CDCl_3_, 125 MHz) δ 164.1, 163.7, 143.7, 138.8, 132.4, 132.2, 131.4, 130.5, 130.3, 130.2, 128.3, 127.1, 123.3, 120.6, 115.8, 21.3; HRMS–ESI (*m*/*z*): [M + H]^+^ calcd for C_31_H_20_N_2_O_2_S, 485.1245; found, 485.1325.

### Synthesis of **NI-PTZ-OCH_3_**

**NI-PTZ-OCH_3_** was synthesized by a method similar to that of **NI-PTZ-F**. The crude product was purified by column chromatography (silica gel, DCM/PE 1:5, v:v). The product was obtained as orange solid. Yield: 55 mg (22%); mp 123.2–124.3 °C. ^1^H NMR (CDCl_3_, 400 MHz) δ 8.87 (d, *J* = 7.63 Hz, 1H), 8.70 (d, *J* = 7.25 Hz, 1H), 8.58 (d, *J* = 8.51 Hz, 1H), 7.98 (d, *J* = 7.63 Hz, 1H), 7.75–7.79 (m, 1H), 7.27 (s, 1H), 7.25 (s, 1H), 7.08–7.12 (m, 4H), 6.76–6.87 (m, 4H), 6.08 (d, *J* = 8.13 Hz, 2H), 3.89 (s, 3H); ^13^C NMR (CDCl_3_, 125 MHz) δ 164.0, 163.7, 143.7, 143.4, 132.2, 131.4, 130.4, 129.5, 128.9, 128.6, 128.3, 127.1, 127.1, 123.3, 120.6, 115.8, 56.1; HRMS–ESI (*m*/*z*): [M + H]^+^ calcd for C_31_H_20_N_2_O_3_S, 501.1195; found, 501.1276.

### Synthesis of **NI-PTZ-F-O**

Compound **NI-PTZ-F-O** was synthesized by a modified literature method [43]. Compound **NI-PTZ-F** (36 mg, 0.074 mmol) was dissolved in glacial acetic acid (5 mL), H_2_O_2_ (1.5 mL, 30%, 1.184 mmol) was added dropwise and the mixture was stirred at 40 °C for 1 h. Then, the reaction mixture was poured into water and the pH of the mixture was brought to 7 by the addition of saturated aqueous solution of Na_2_CO_3_. After cooling, water (10 mL) was added, and the mixture was extracted with ethyl acetate (3 × 20 mL). The organic layer was separated and washed with water and brine solution (3 × 20 mL), respectively. The combined organic layers were dried over anhydrous Na_2_SO_4_ and the solvent was evaporated under reduced pressure. The crude product was purified by column chromatography (silica gel, DCM/MeOH 50:1, v:v) and the product was obtained as yellow solid. Yield: 28 mg (76%); mp 100.1–101.0 °C; ^1^H NMR (CDCl_3_, 400 MHz) δ 8.87 (d, *J* = 7.63 Hz, 1H), 8.70 (d, *J* = 7.26 Hz, 1H), 8.59 (d, *J* = 7.26 Hz, 1H), 8.00 (d, *J* = 7.26 Hz, 1H), 7.76–7.80 (m, 1H), 7.30 (d, *J* = 8.26 Hz, 4H), 7.11 (d, *J* = 7.51 Hz, 2H), 6.85–6.88 (m, 2H), 6.78–6.80 (m, 2H), 6.50 (d, *J* = 7.51 Hz, 2H); ^13^C NMR (CDCl_3_, 125 MHz) δ 163.7, 163.4, 140.9, 140.3, 133.2, 133.1, 132.0, 130.6, 130.4, 130.3, 123.8, 123.6, 123.1, 116.8, 116.7, 116.5; HRMS–MALDI (*m*/*z*): [M + H]^+^ calcd for C_30_H_17_FN_2_O_3_S, 505.0944; found, 505.0973.

### Electrochemical studies

The cyclic voltammetry curves were recorded with a CHI610D electrochemical workstation (CHI instruments, Inc., Shanghai, China) in N_2_-purged saturated solutions containing 0.10 M Bu_4_NPF_6_ as a supporting electrolyte, a platinum electrode as counter electrode, a glassy carbon electrode as working electrode, and the Ag/AgNO_3_ (0.1 M in ACN) couple as the reference electrode. Ferrocenium/ferrocene (Fc^+^/Fc) redox couple was used as an internal reference.

### Femtosecond transient absorption spectroscopy

Femtosecond transient absorption spectra (fs-TA) were acquired on a system based on a Ti:sapphire regenerative amplifier (Coherent Astrella). The system produces 40 fs pulses at 800 nm, with a repetition rate of 1 kHz. The instrument resolution is 100 fs. Excitation pulses at 340 nm were produced by a commercial parametric amplifier. The pump beam polarization was set to the magic angle with respect to the probe beam. The probe beam was obtained by focusing a small portion of the fundamental 800 nm beam on a 2 mm CaF_2_ crystal, kept under continuous rotation to avoid damage. The pump-probe delay was introduced by sending the portion of the fundamental beam used for white light generation through a motorized translator. After focusing and overlapping the pump and probe beams at the sample position, the probe beam was directed through a spectrograph and to the detector. The sample was contained in a 1 mm quartz cuvette mounted on a movable stage, in order to avoid photodecomposition. The data were subdivided in two different time intervals and fitted using the Glotaran-Application 1.5.1 and Surface Xplorer software. The number of kinetic components to be used for Global analysis was estimated by performing a preliminary singular values decomposition (SVD) analysis. All data were chirp-corrected before global fitting.

### Nanosecond transient absorption spectroscopy

The nanosecond transient absorption spectra were measured on a LP920 laser flash photolysis spectrometer (Edinburgh Instruments, Ltd., U.K.). The data (kinetic decay traces and the transient difference absorption spectra) were analyzed with the L900 software. All samples were deaerated with N_2_ for ca. 15 min before measurement and excited with a nanosecond pulsed laser (OPO nanosecond pulsed laser). The wavelength is tunable in the range of 410–2500 nm.

### Calculation study

The geometries of the compounds in their ground state were optimized using density functional theory (DFT) with the B3LYP functional and the 6-31G(d) basis set [72]. The excited state geometries of S_1_ and T_1_ were optimized with time-dependent TD-DFT (DFT) using the same functional and basis sets as in the ground state optimizations [73]. The spin density of the compounds were optimized using DFT with the B3LYP functional and 6-31G(d) basis set. The excitation energy of the compounds was calculated by TD-DFT at the B3LYP/6-31G(d) level of theory.

## Supporting Information

File 1 General experimental methods, ^1^H NMR, ^13^C NMR, and HRMS spectra of the compounds as well as theoretical computation and photophysical data.
