# Supplementary material for: The effect of dark states on the intersystem crossing and thermally activated delayed fluorescence of naphthalimide-phenothiazine dyads
Source: Beilstein J Org Chem. 2023 Jul 19;19:1028–46. doi: 10.3762/bjoc.19.79 (PMC10366440; doi:10.3762/bjoc.19.79)
Supplement: File 1 — General experimental methods, 1H NMR, 13C NMR, and HRMS spectra of the compounds as well as theoretical computation and photophysical data. [file Beilstein_J_Org_Chem-19-1028-s001.pdf]

BEILSTEIN JOURNAL OF ORGANIC CHEMISTRY

Supporting Information

for

The effect of dark states on the intersystem crossing and thermally activated delayed fluorescence of naphthalimide-phenothiazine dyads

Liyuan Cao, Xi Liu, Xue Zhang, Jianzhang Zhao, Fabiao Yu and Yan Wan

Beilstein J. Org. Chem. **2023,** *19,* 1028–1046. doi:10.3762/bjoc.19.79

General experimental methods, ^{1}H NMR, ^{13}C NMR, and HRMS spectra of the compounds as well as theoretical computation and photophysical data

License and Terms: This is a supporting information file under the terms of the Creative Commons Attribution License (https://creativecommons.org/licenses/by/4.0). Please note that the reuse, redistribution and reproduction in particular requires that the author(s) and source are credited and that individual graphics may be subject to special legal provisions.
The license is subject to the *Beilstein Journal of Organic Chemistry* terms and conditions: (https://www.beilstein-journals.org/bjoc/terms)

Table of contents

1. General information··································S2

2. Synthesis of the compounds··································S2

3. Molecular structure characterization data··································S5

4. Steady state UV–vis absorption and luminescence spectra··································S20

5. Fluorescence lifetimes··································S22

6. Thermal stability analysis (thermogravimetry analysis, TGA)··································S23

7. Femtosecond transient absorption spectroscopy··································S23

8. Nanosecond transient absorption spectroscopy··································S24

9. Theoretical computation··································S26

10. Coordinates of the optimized geometries of the compounds··································S29

1. General information

All the chemicals used in synthesis are analytically pure and were used as received. Solvents were dried and distilled prior to use. ^{1}H and ^{13}C NMR spectra were recorded on a Bruker Avance spectrometer (400/500 MHz). ^{1}H and ^{13}C chemical shifts are reported in parts per million (ppm) relative to TMS, with the residual solvent peak used as an internal reference. The mass spectra were measured by HRMS (MALDI-TOF, recorded on a Bruker Ultraflextreme mass spectrometer) and HRMS (ESI-TOF, recorded on an Agilent G6224A mass spectrometer). UV–vis absorption spectra were measured on a UV-2550 UV–vis spectrophotometer (Shimadzu Ltd., Japan). Fluorescence spectra were recorded with an FS5 spectrofluorometer (Edinburgh instruments, UK). Luminescence lifetimes of compounds were recorded with an OB920 luminescence lifetime spectrometer (Edinburgh Instruments, U.K.). All these calculations were performed with Gaussian 09W [1]. Natural transition orbital analysis was performed by the Multiwfn program [2].

2. Synthesis of the compounds

2.1. Synthesis of NI-PTZ-C$_5$. NI-PTZ-C$_5$ was synthesized by a method similar to that of **NI-PTZ-F**. The crude product was purified by column chromatography (silica gel, DCM:PE = 1:2, v:v). The product was obtained as orange solid. Yield: 20 mg (46%). M.p.: 110.5–111.6 °C. ^{1}H NMR ($CDCl_3$, 400 MHz) *δ*: 8.82 (d, *J* = 7.76 Hz, 1H), 8.65 (d, *J* = 6.38 Hz, 1H), 8.52 (d, *J* = 9.26 Hz, 1H), 7.94 (d, *J* = 7.63 Hz, 1H), 7.71–7.75 (m, 1H), 7.09 (d, *J* = 7.50 Hz, 2H), 6.74–6.86 (m, 4H), 6.04 (d, *J* = 7.50 Hz, 2H), 4.22 (t, *J* = 15.26 Hz, 2H), 1.74–1.81 (m, 2H), 1.47 (t, *J* = 28.02 Hz, 4H), 0.94 (t, *J* = 14.01 Hz, 3H). ^{13}C NMR ($CDCl_3$, 125 MHz) *δ*: 163.7, 143.8, 143.4, 131.9, 131.6, 130.8, 129.4, 128.8, 128.6, 128.3, 127.4, 126.9, 122.2, 118.8, 115.8, 40.7, 29.2, 27.8, 22.5, 14.0. HRMS (ESI, *m/z*) calcd for $C_{29}H_{24}N_2O_2S$ $[M+H]^+$, 465.1558, found 465.1623.

2.2. Synthesis of NI-PTZ-Ph-O. NI-PTZ-Ph-O was synthesized by a method similar to that of **NI-PTZ-F-O**. The crude product was purified by column chromatography (silica gel, DCM:MeOH = 50:1, v:v). **NI-PTZ-Ph-O** was obtained as yellow solid. Yield: 40 mg

(82%). M.p.: 176.2–177.2 °C. ^{1}H NMR ($CDCl_3$, 400 MHz) *δ*: 8.86 (d, *J* = 7.62 Hz, 1H), 8.70 (d, *J* = 7.62 Hz, 1H), 8.57 (d, *J* = 7.63 Hz, 1H), 7.99 (d, *J* = 7.63 Hz, 1H), 7.77–7.80 (m, 1H), 7.57–7.60 (m, 2H), 7.51–7.54 (m, *J* = 8.26 Hz, 1H), 7.35 (d, *J* = 7.25 Hz, 2H), 7.11 (d, *J* = 7.49 Hz, 2H), 6.77–6.87 (m, 4H), 6.49 (d, *J* = 7.73 Hz, 2H). ^{13}C NMR ($CDCl_3$, 125 MHz) *δ*: 163.6, 163.2, 140.6, 133.4, 132.7, 131.9, 130.6, 129.9, 129.5, 129.2, 123.7, 123.6, 123.0, 116.7. HRMS (MALDI, m/z) calcd for $C_{30}H_{18}N_2O_3S$ $[M+H]^+$, 487.1038, found 487.1054.

2.3. Synthesis of NI-PTZ-C$_5$-O. NI-PTZ-C$_5$-O was synthesized by a method similar to that of **NI-PTZ-Ph-O**. The crude product was purified by column chromatography (silica gel, DCM:MeOH = 50:1, v:v). **NI-PTZ-C$_5$-O** was obtained as yellow solid. Yield: 34 mg (69%). M.p.: 120.1–121.3 °C. ^{1}H NMR ($CDCl_3$, 400 MHz) *δ*: 8.81 (d, *J* = 7.76 Hz, 1H), 8.60 (d, *J* = 7.76 Hz, 1H), 8.52 (d, *J* = 7.75 Hz, 1H), 7.94 (d, *J* = 7.63 Hz, 1H), 7.71–7.75 (m, 1H), 7.07 (d, *J* = 7.50 Hz, 2H), 6.74–6.84 (m, 4H), 6.49 (d, *J* = 7.52 Hz, 2H), 4.22 (t, *J* = 15.26 Hz, 2H), 1.74–1.81 (m, 2H), 1.43 (s, 4H), 0.94 (t, *J* = 14.02 Hz, 3H). ^{13}C NMR ($CDCl_3$, 125 MHz) *δ*: 163.6, 163.2, 140.3, 133.2, 132.5, 131.5, 130.4, 129.7, 129.2, 129.0, 123.7, 123.5, 122.9, 116.9, 40.7, 29.2, 27.8, 22.5, 14.0. HRMS (MALDI, *m/z*) calcd for $C_{29}H_{24}N_2O_3S$ $[M+H]^+$, 481.1508, found 481.1512.

2.4. Synthesis of F-NI-Br. Compound **F-NI-Br** was synthesized in a manner similar to the reported literature [3]. A mixture of 4-bromo-1,8-naphthalene anhydride (500 mg, 1.8 mmol) and 4-fluoroaniline (1.7 mL, 18.1 mmol) was dissolved in acetic acid (21 mL). The solution was refluxed under nitrogen overnight. After cooling to room temperature, the mixture was poured into water (30 mL). The precipitate was filtered and recrystallized to afford compound **F-NI-Br** as yellow crystals. Yield: 480 mg (72%). M.p.: 98.8–99.6 °C. ^{1}H NMR ($CDCl_3$, 400 MHz) *δ*: 8.64–8.72 (m, 2H), 8.46 (d, *J* = 7.75 Hz, 1H), 8.09 (m, *J* = 7.88 Hz, 1H), 7.88–7.92 (m, 1H), 7.56 (d, *J* = 7.75 Hz, 2H), 7.29–7.31 (m, 1H), 7.23 (d, *J* = 8.63 Hz, 1H).

2.5. Synthesis of Ph-NI-Br. Compound **Ph-NI-Br** was synthesized by a method similar to that of **F-NI-Br**. The precipitate was filtered and recrystallized to afford compound **Ph-NI-Br** as yellow crystals. Yield: 565 mg (89%). M.p.: 96.5–97.3 °C. ^{1}H NMR ($CDCl_3$, 400 MHz) *δ*: 8.71 (d, *J* = 7.12 Hz, 1H), 8.65 (d, *J* = 8.51 Hz, 1H), 8.47 (d, *J* = 7.88 Hz, 1H),

8.09 (d, *J* = 7.75 Hz, 1H), 7.88–7.92 (m, 1H), 7.55–7.59 (m, 2H), 7.48–7.52 (m, 1H), 7.32 (d, *J* = 7.38 Hz, 2H).

2.6. Synthesis of CH_3-NI-Br. Compound **CH_3-NI-Br** was synthesized by a method similar to that of **F-NI-Br** The precipitate was filtered and recrystallized to afford compound **CH_3-NI-Br** as yellow crystals. Yield: 520 mg (83%). M.p.: 100.3–100.9 °C. ^1H NMR ($CDCl_3$, 400 MHz) *δ*: 8.71 (d, *J* = 7.00 Hz, 1H), 8.64 (d, *J* = 8.51 Hz, 1H), 8.47 (d, *J* = 7.75 Hz, 1H), 8.08 (d, *J* = 7.75 Hz, 1H), 7.87–7.91 (m, 1H), 7.36 (d, *J* = 8.00 Hz, 2H), 7.20 (d, *J* = 8.13 Hz, 2H), 2.45 (s, 3H).

2.7. Synthesis of OCH_3-NI-Br. Compound **OCH_3-NI-Br** was synthesized by a method similar to that of **F-NI-Br**. The precipitate was filtered and recrystallized to afford compound **OCH_3-NI-Br** as white crystals. Yield: 550 mg (75%). M.p.: 98.9–99.9 °C. ^1H NMR ($CDCl_3$, 400 MHz) *δ*: 8.54 (s, 2H), 8.29 (s, 1H), 7.76 (s, 1H), 7.52 (d, *J* = 4.63 Hz, 2H), 7.30 (s, 1H), 7.19–7.22 (m, 2H), 4.02 (t, *J* = 15.38 Hz, 3H).

3. Molecular structure characterization data

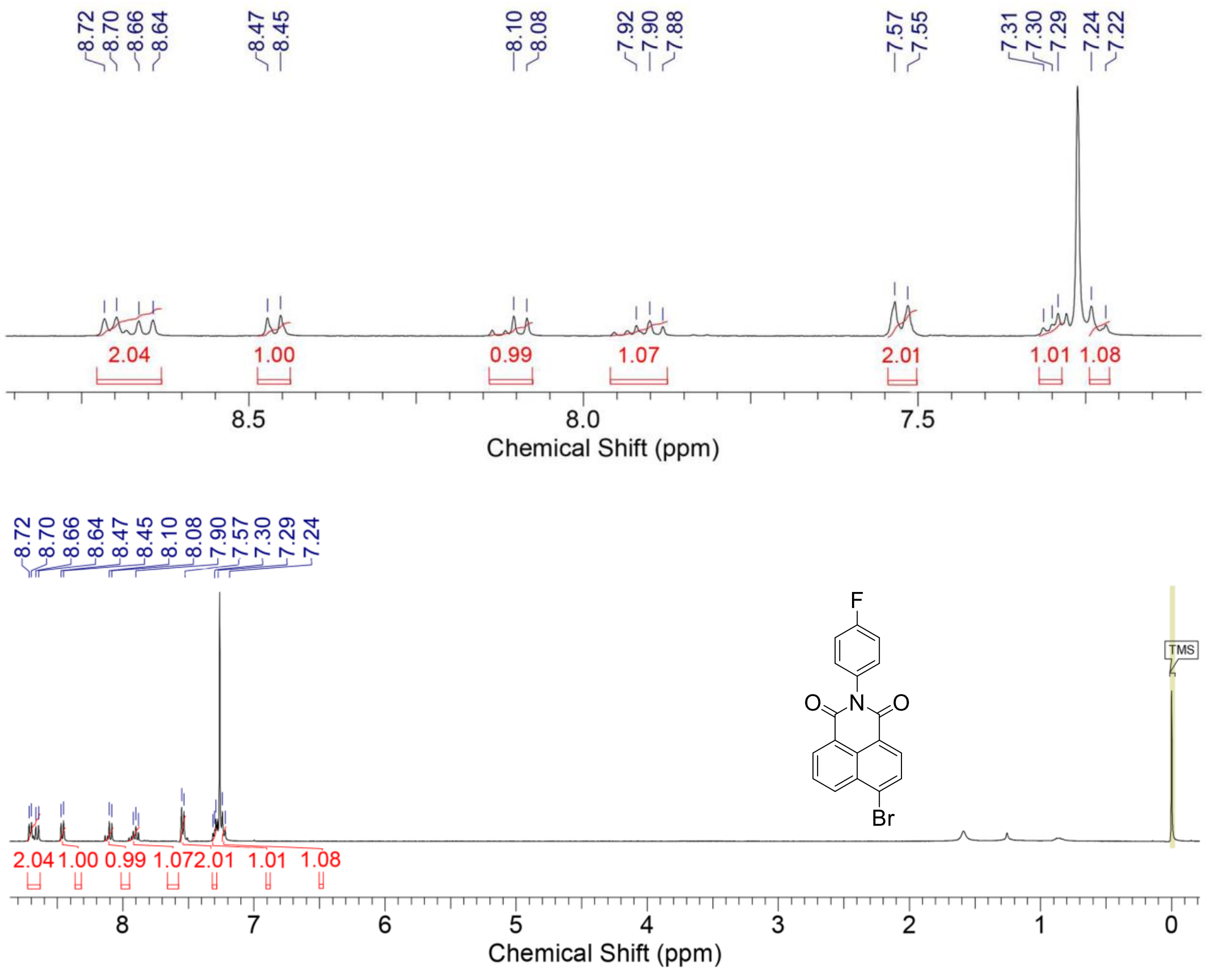

Figure S1. ^{1}H NMR spectrum of compound **F-NI-Br** in $CDCl_3$ (400 MHz), 25 °C.

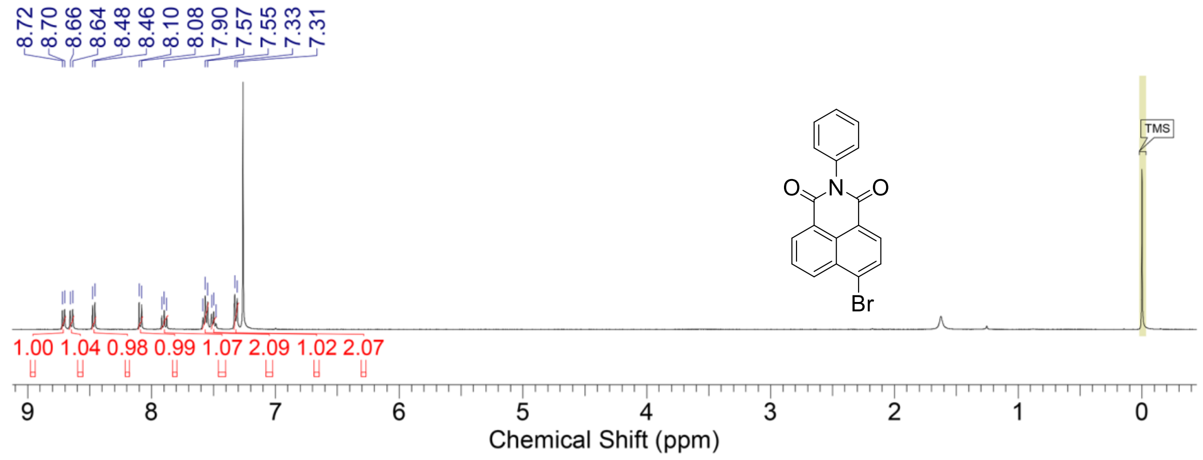

Figure S2. ^{1}H NMR spectrum of compound **Ph-NI-Br** in $CDCl_3$ (400 MHz), 25 °C.

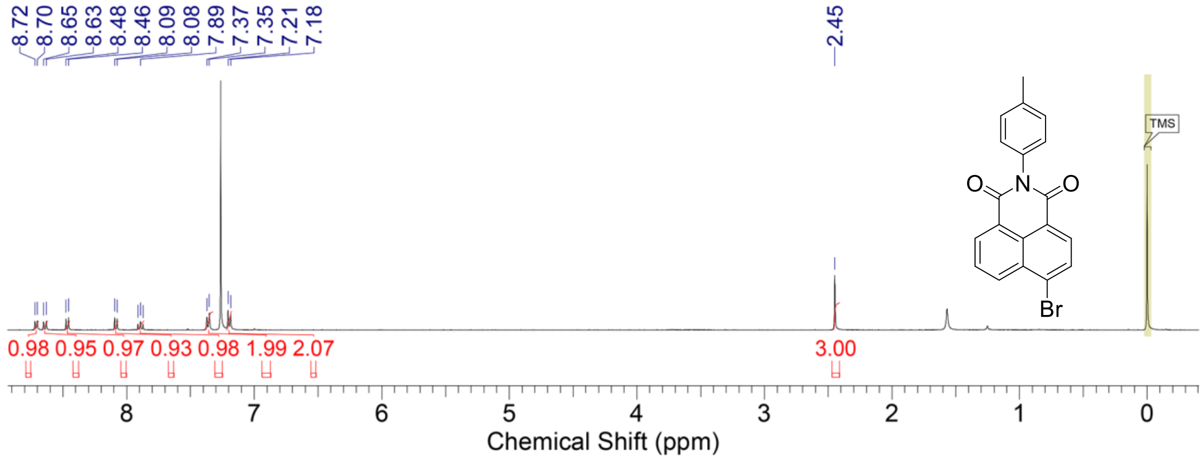

Figure S3. ¹H NMR spectrum of compound **CH_3-NI-Br** in $CDCl_3$ (400 MHz), 25 °C.

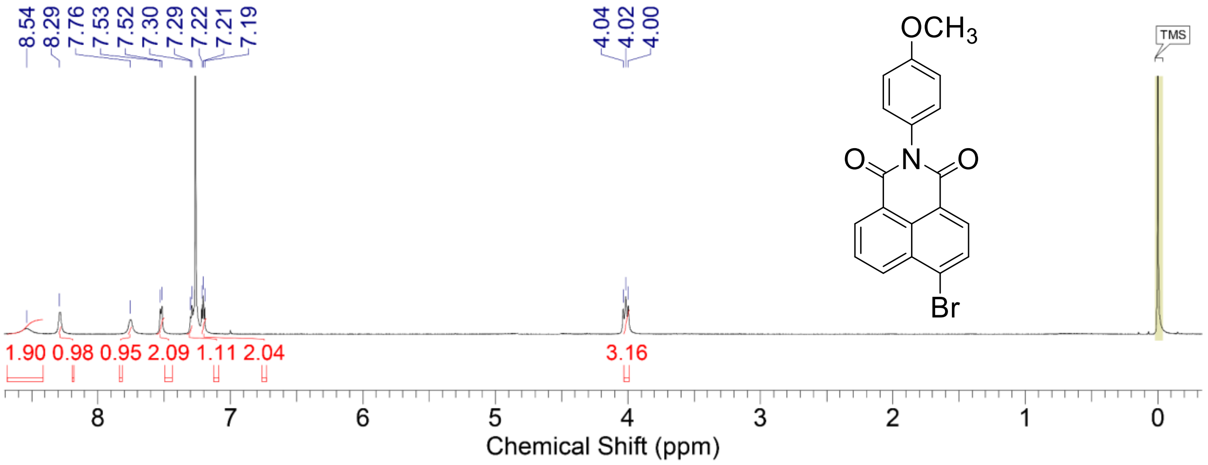

Figure S4. ¹H NMR spectrum of compound **OCH_3-NI-Br** in $CDCl_3$ (400 MHz), 25 °C.

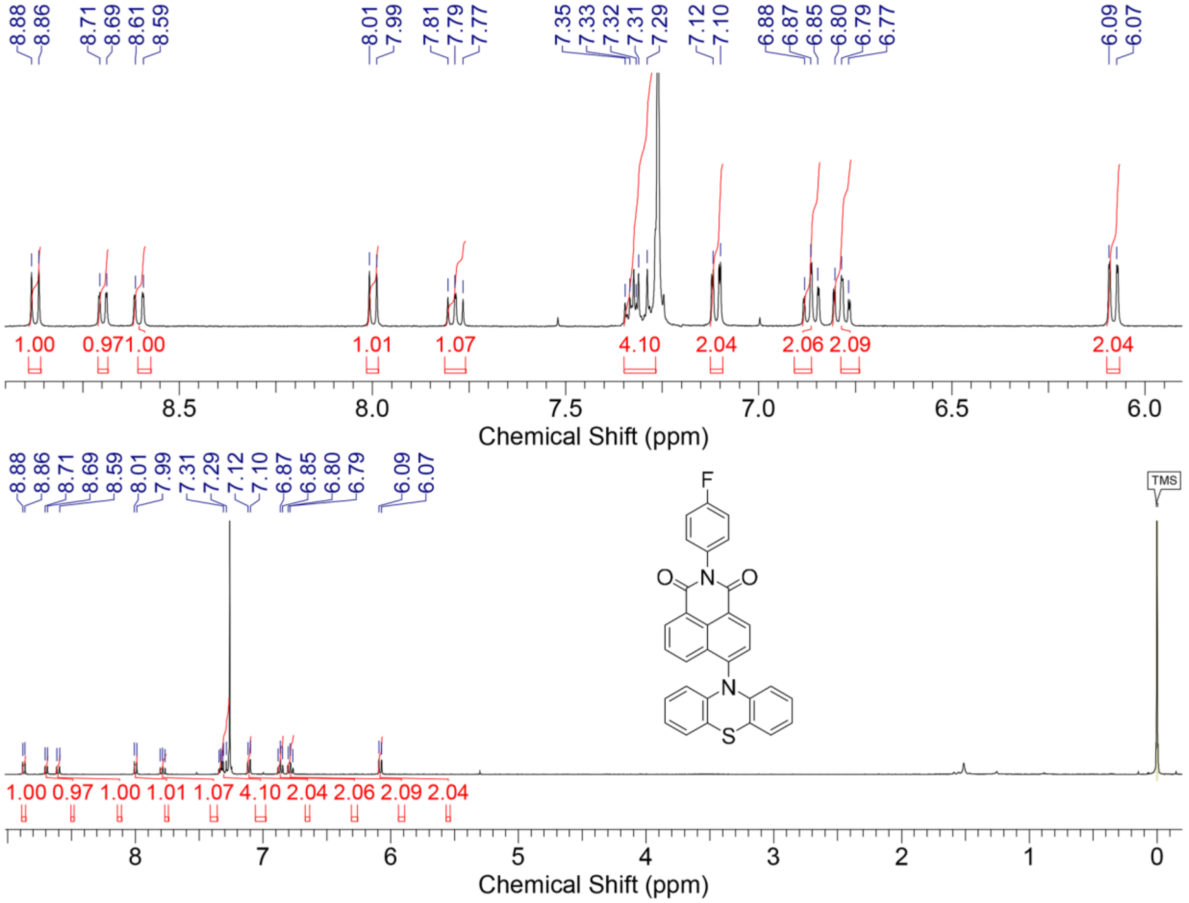

Figure S5. ^{1}H NMR spectrum of compound **NI-PTZ-F** in $CDCl_3$ (400 MHz), 25 °C.

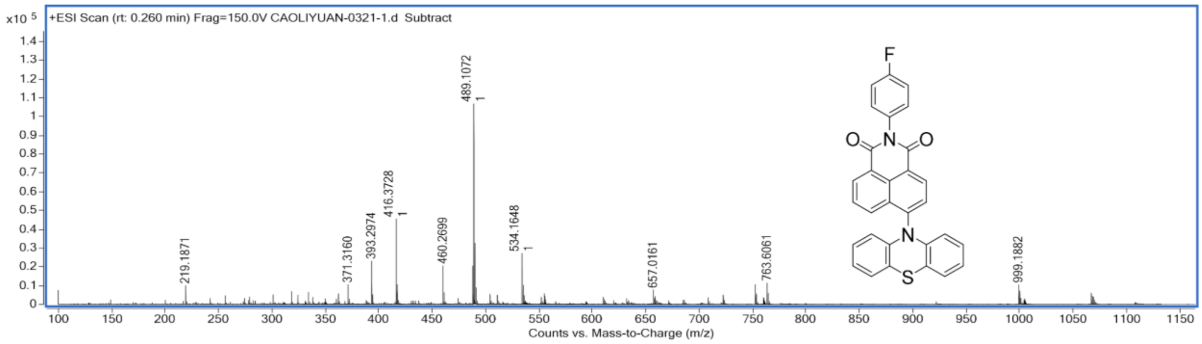

Figure S6. ESI-HRMS spectrum of **NI-PTZ-F**, 25 °C.

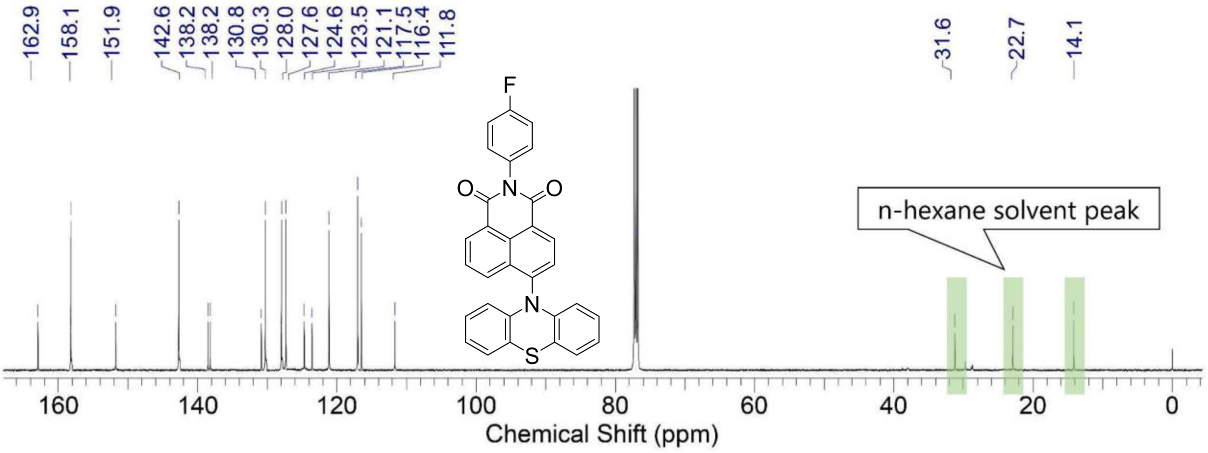

Figure S7. ^{13}C NMR spectrum of **NI-PTZ-F** in $CDCl_3$ (125 MHz), 25 °C.

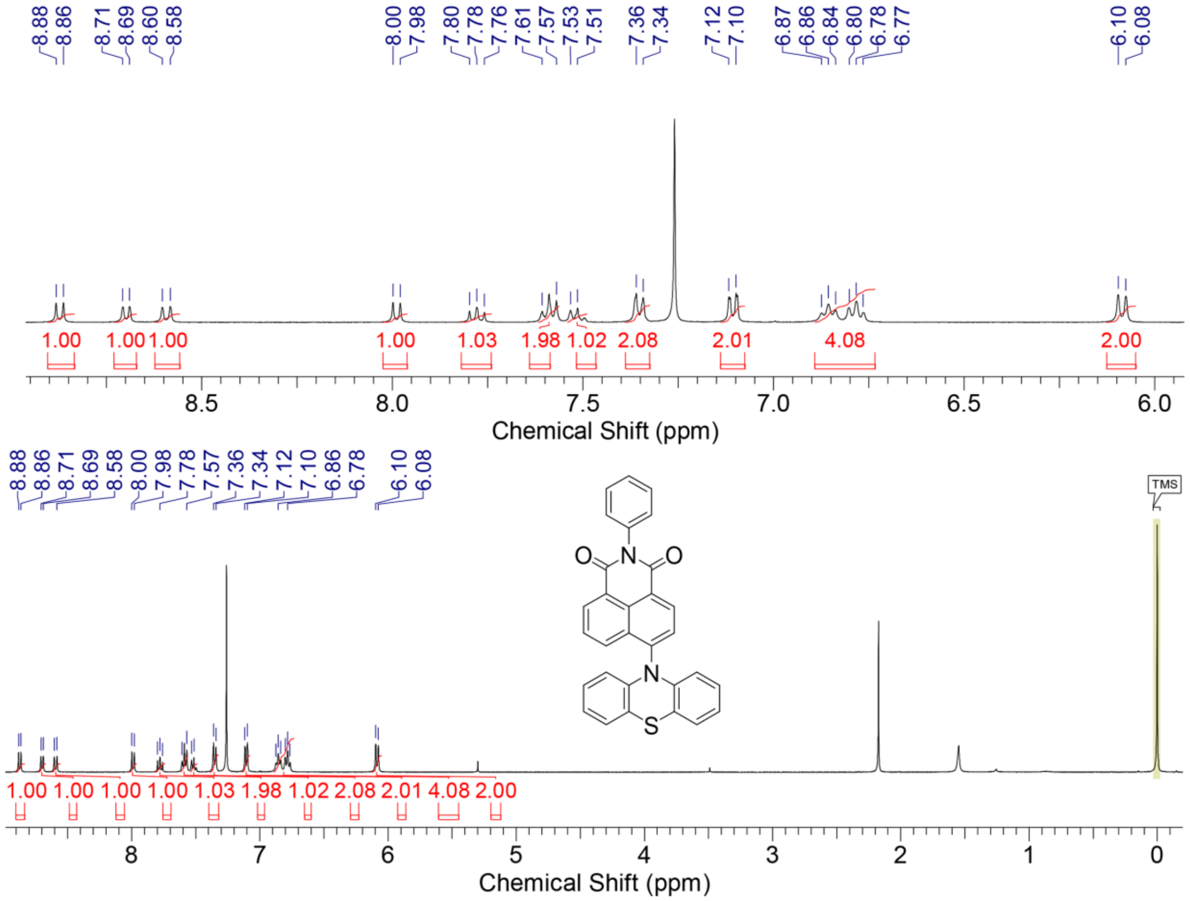

Figure S8. ^{1}H NMR spectrum of **NI-PTZ-Ph** in $CDCl_3$ (400 MHz), 25 °C.

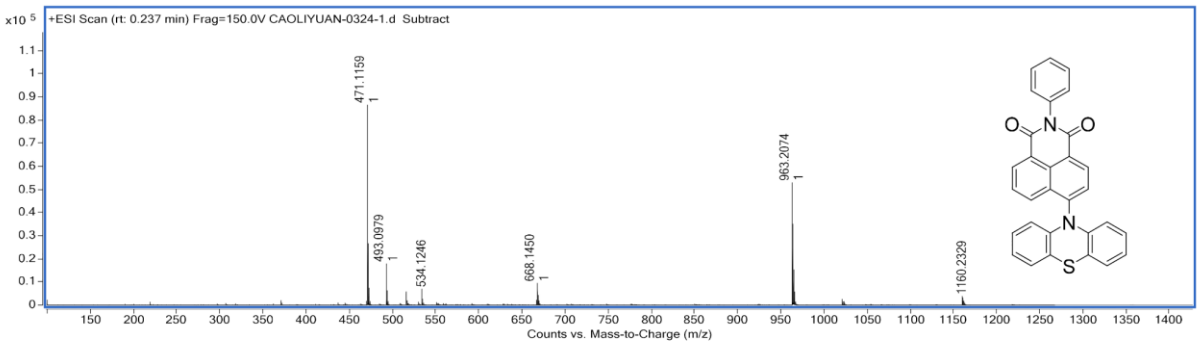

Figure S9. ESI-HRMS spectrum of **NI-PTZ-Ph**, 25 °C.

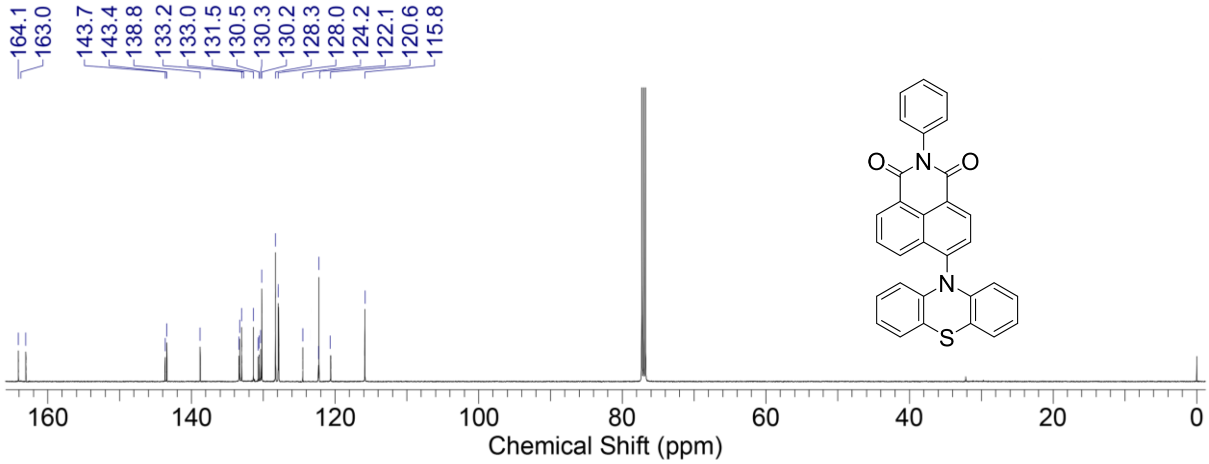

Figure S10. ^{13}C NMR spectrum of **NI-PTZ-Ph** in $CDCl_3$ (125 MHz), 25 °C.

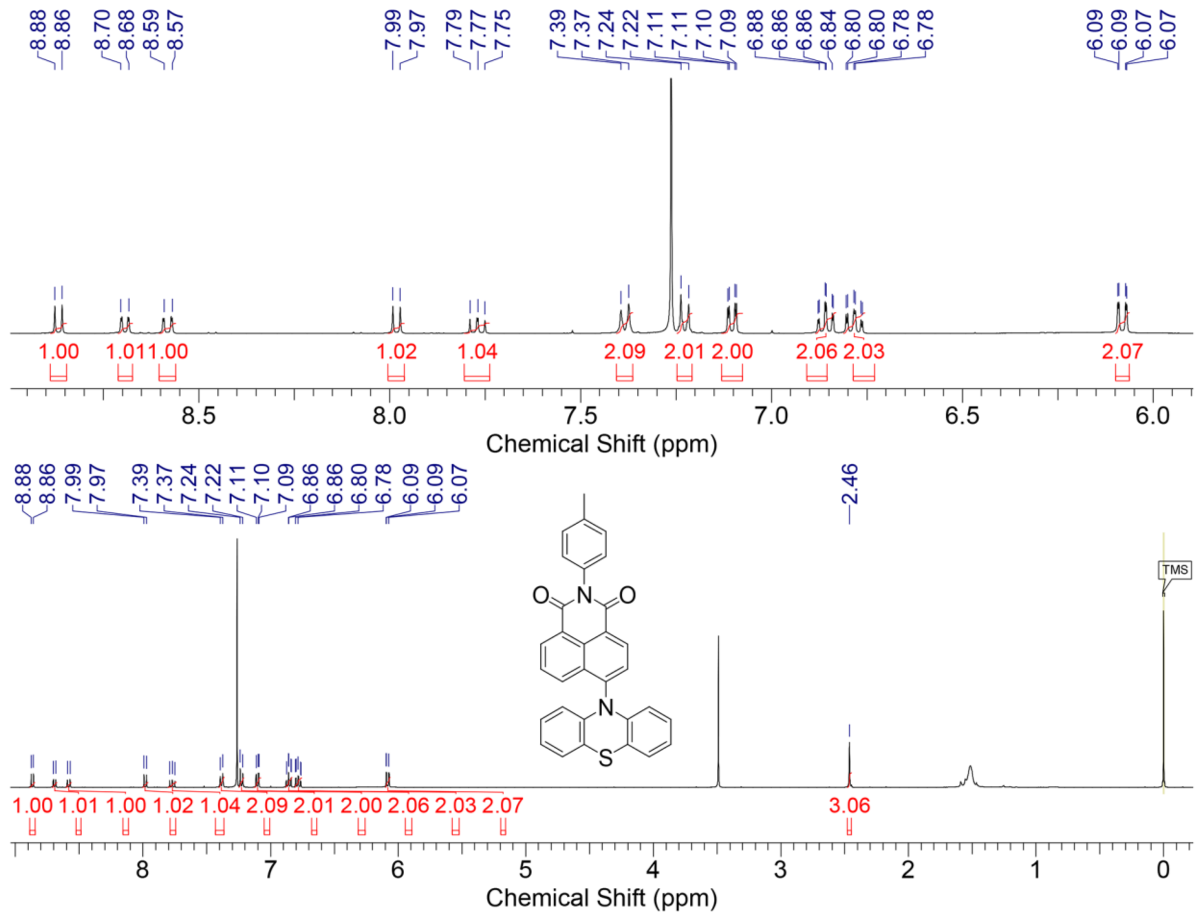

Figure S11. ^{1}H NMR spectrum of **NI-PTZ-CH$_3$** in $CDCl_3$ (400 MHz), 25 °C.

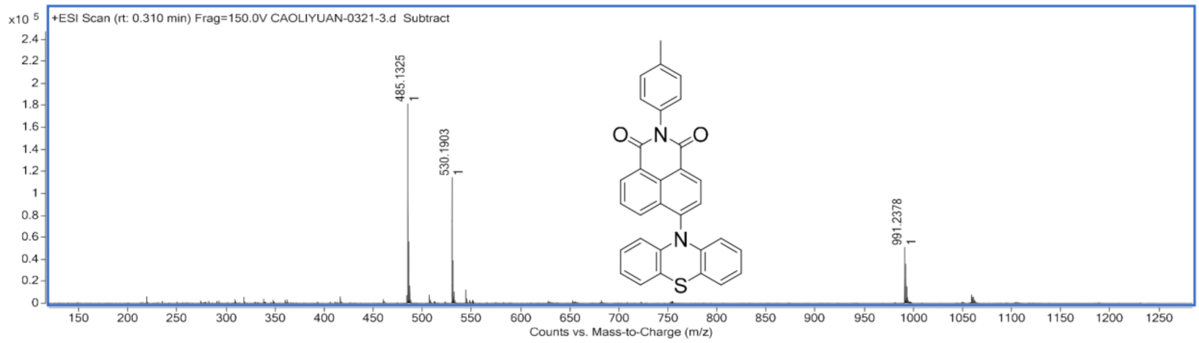

Figure S12. ESI-HRMS spectrum of **NI-PTZ-CH$_3$**, 25 °C.

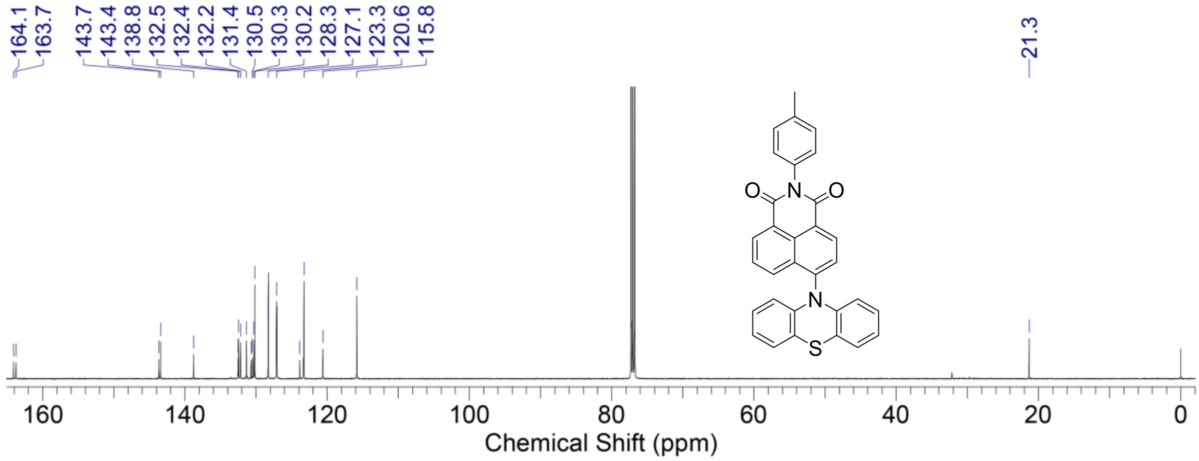

Figure S13. ^{13}C NMR spectrum of **NI-PTZ-CH$_3$** in $CDCl_3$ (125 MHz), 25 °C.

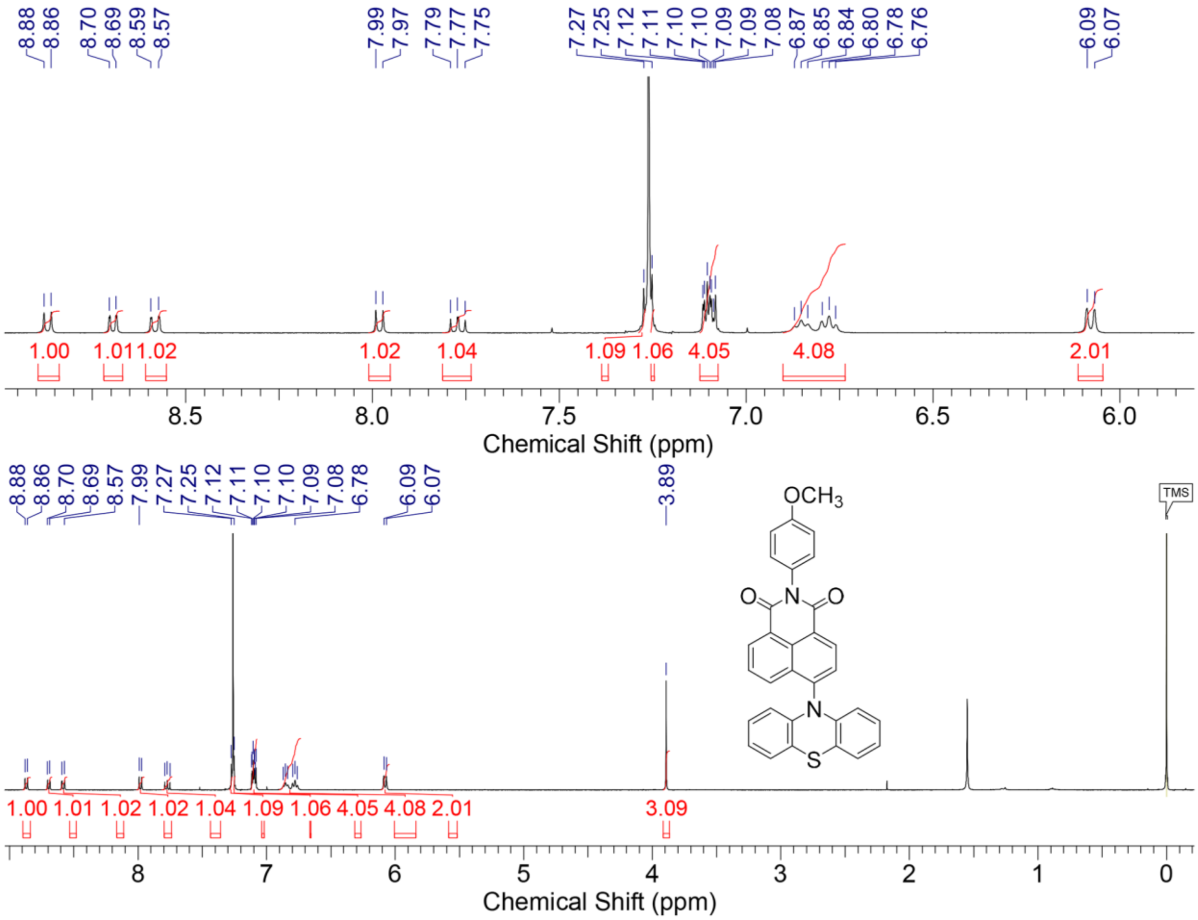

Figure S14. ^{1}H NMR spectrum of **NI-PTZ-OCH$_3$** in $CDCl_3$ (400 MHz), 25 °C.

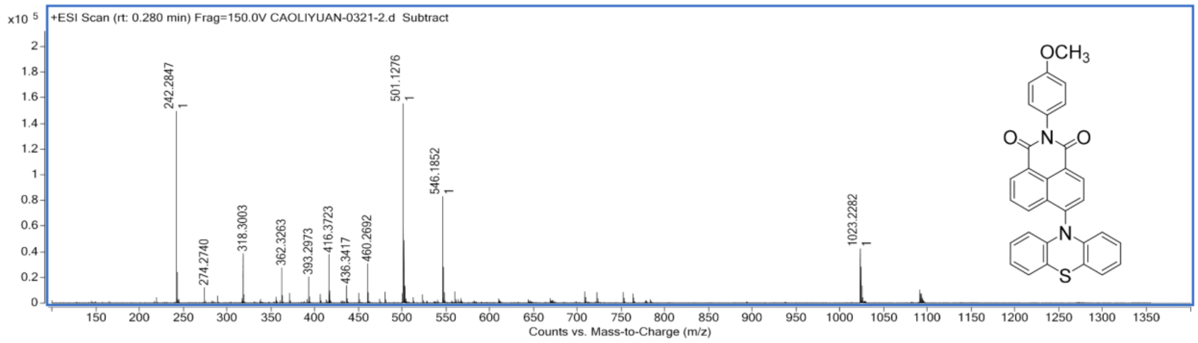

Figure S15. ESI-HRMS spectrum of **NI-PTZ-OCH₃**, 25 °C.

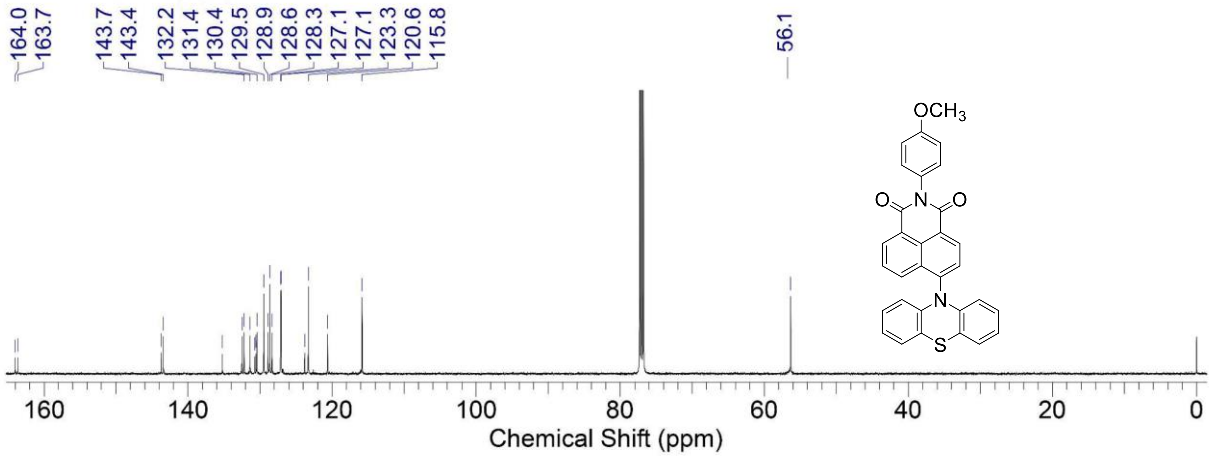

Figure S16. ^{13}C NMR spectrum of **NI-PTZ-OCH₃** in $CDCl_3$ (125 MHz), 25 °C.

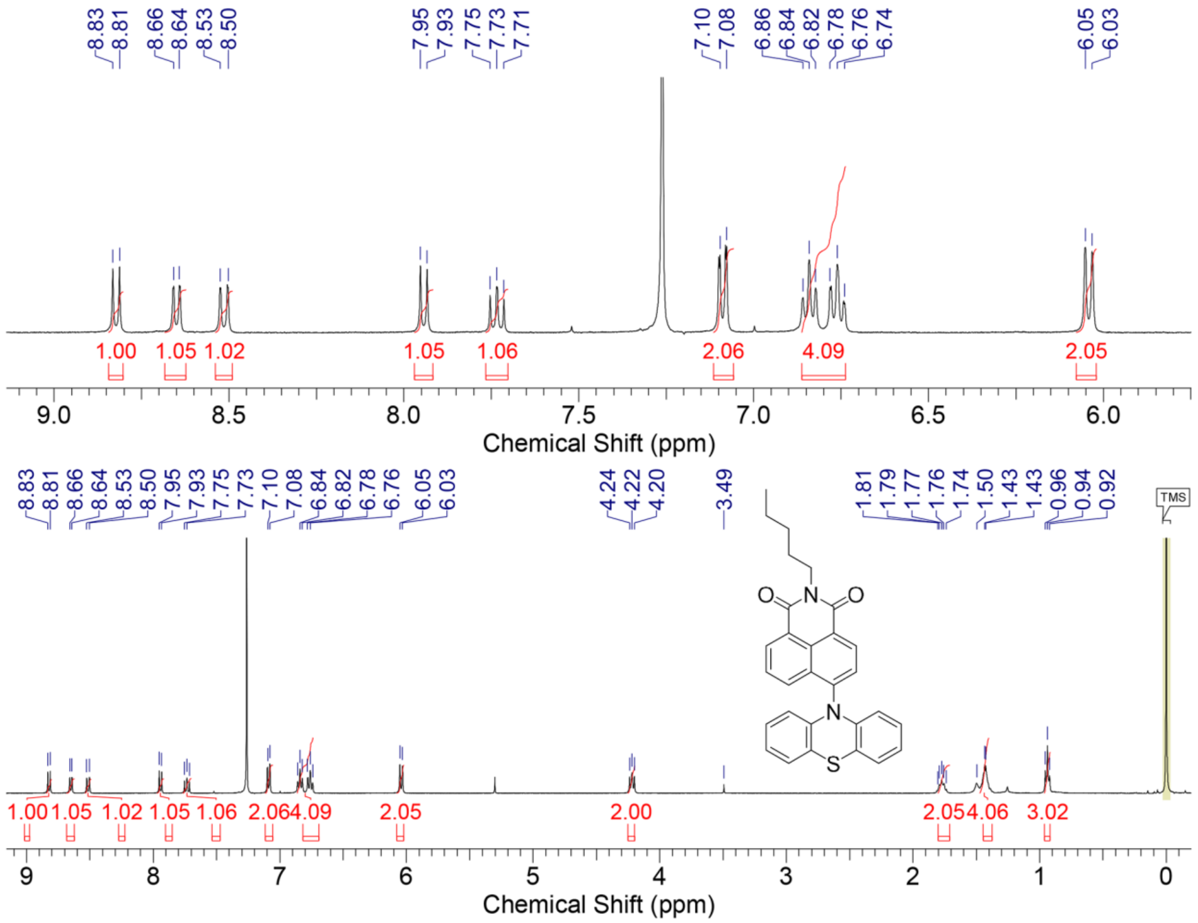

Figure S17. ^{1}H NMR spectrum of **NI-PTZ-C$_5$** in $CDCl_3$ (400 MHz), 25 °C.

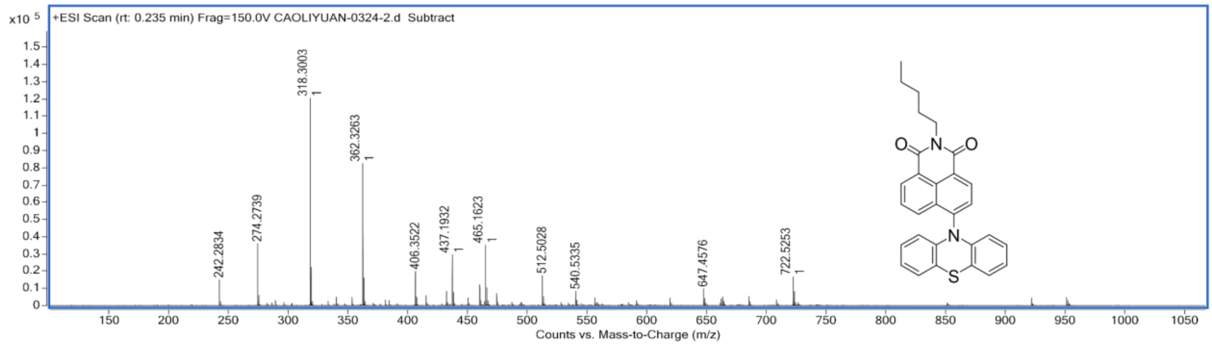

Figure S18. ESI-HRMS spectrum of **NI-PTZ-C$_5$**, 25 °C.

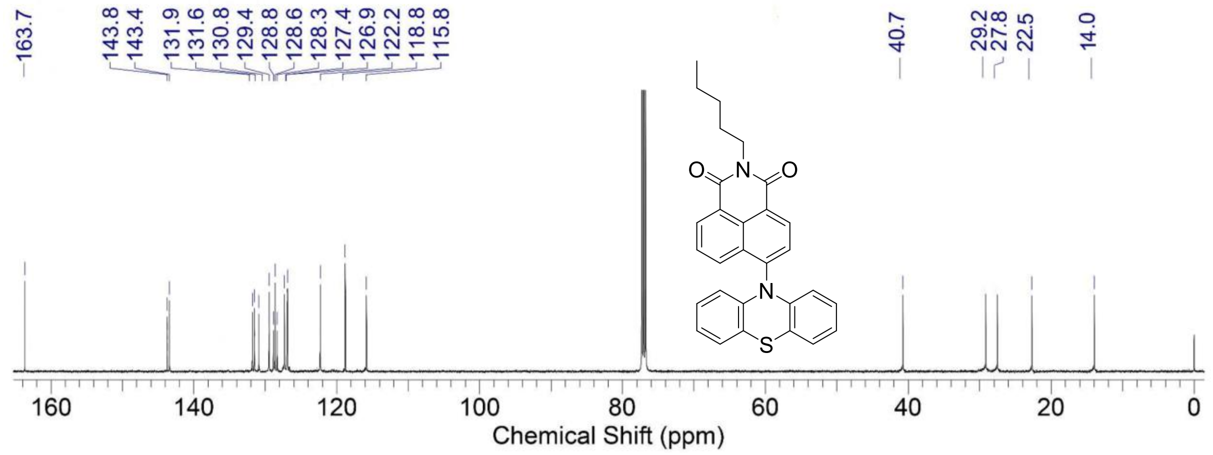

Figure S19. ^{13}C NMR spectrum of **NI-PTZ-C$_5$** in $CDCl_3$ (125 MHz), 25 °C.

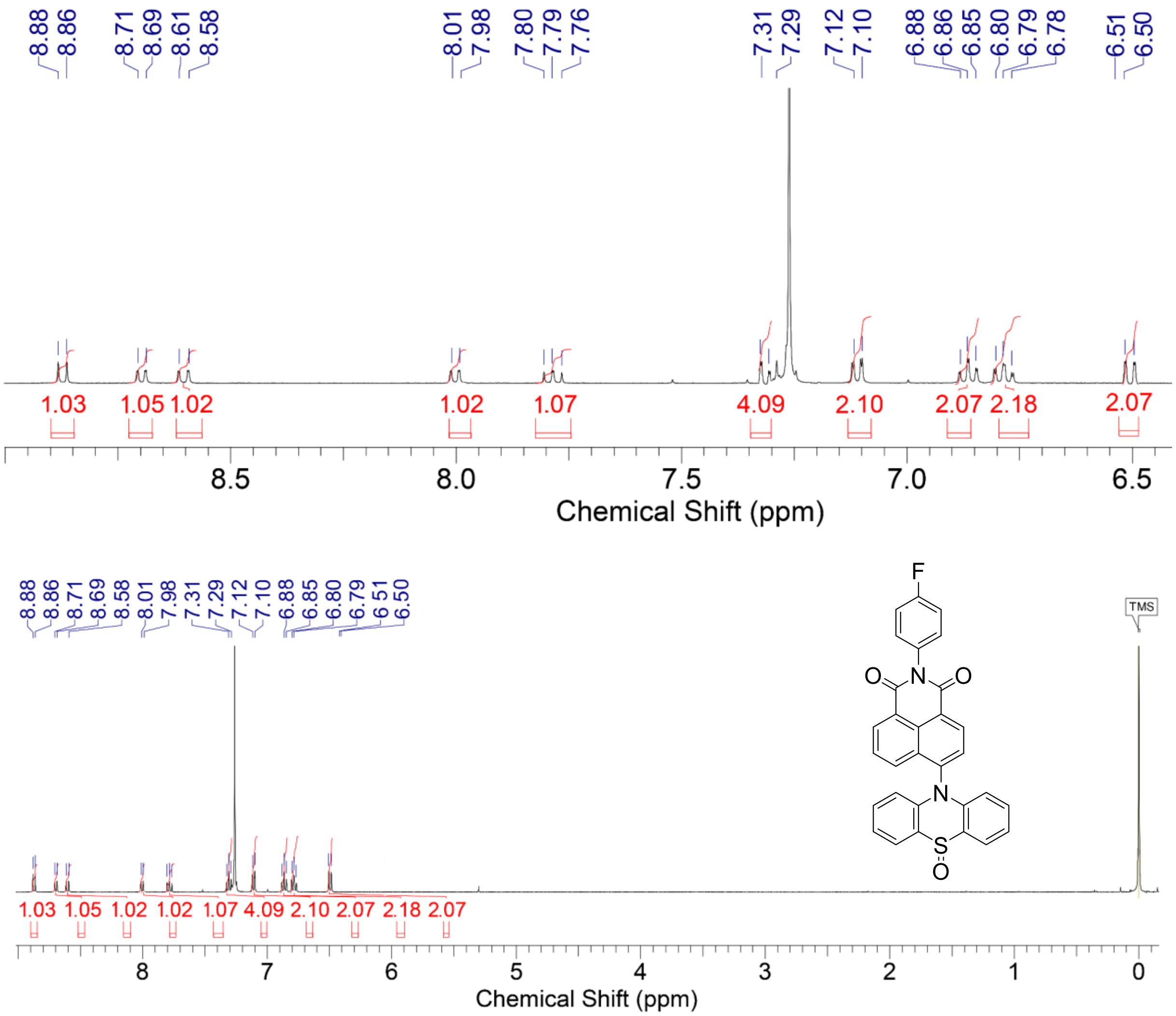

Figure S20. ^{1}H NMR spectrum of **NI-PTZ-F-O** in $CDCl_3$ (400 MHz), 25 °C.

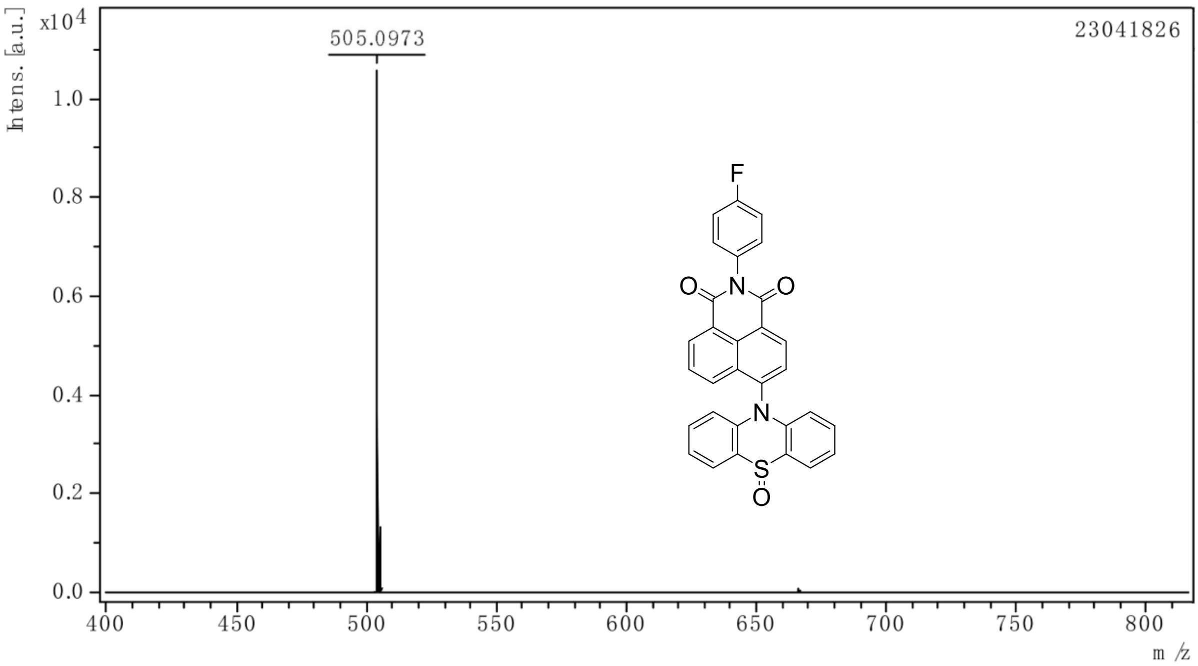

Figure S21. MALDI-HRMS spectrum of **NI-PTZ-F-O**, 25 °C.

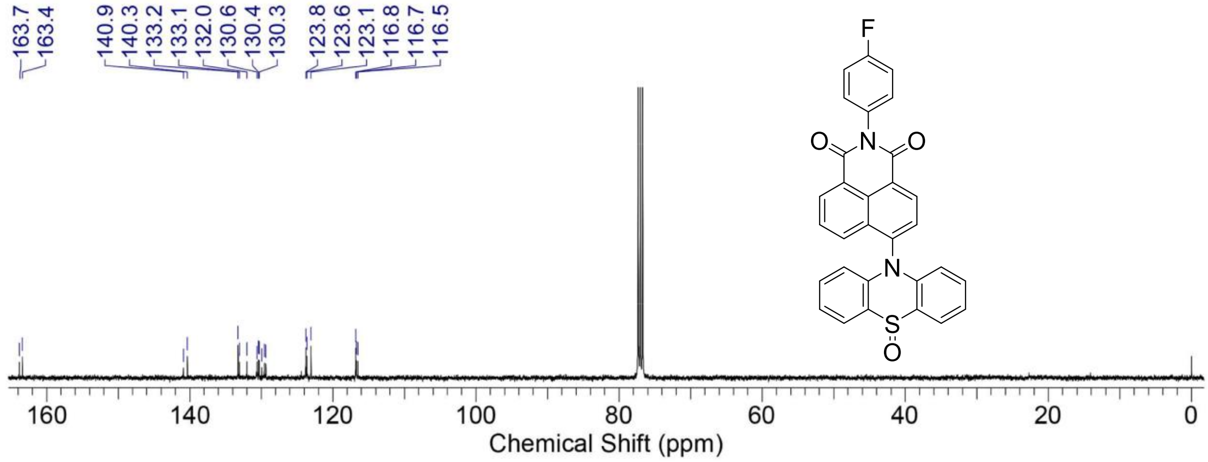

Figure S22. ^{13}C NMR spectrum of **NI-PTZ-F-O** in $CDCl_3$ (125 MHz), 25 °C.

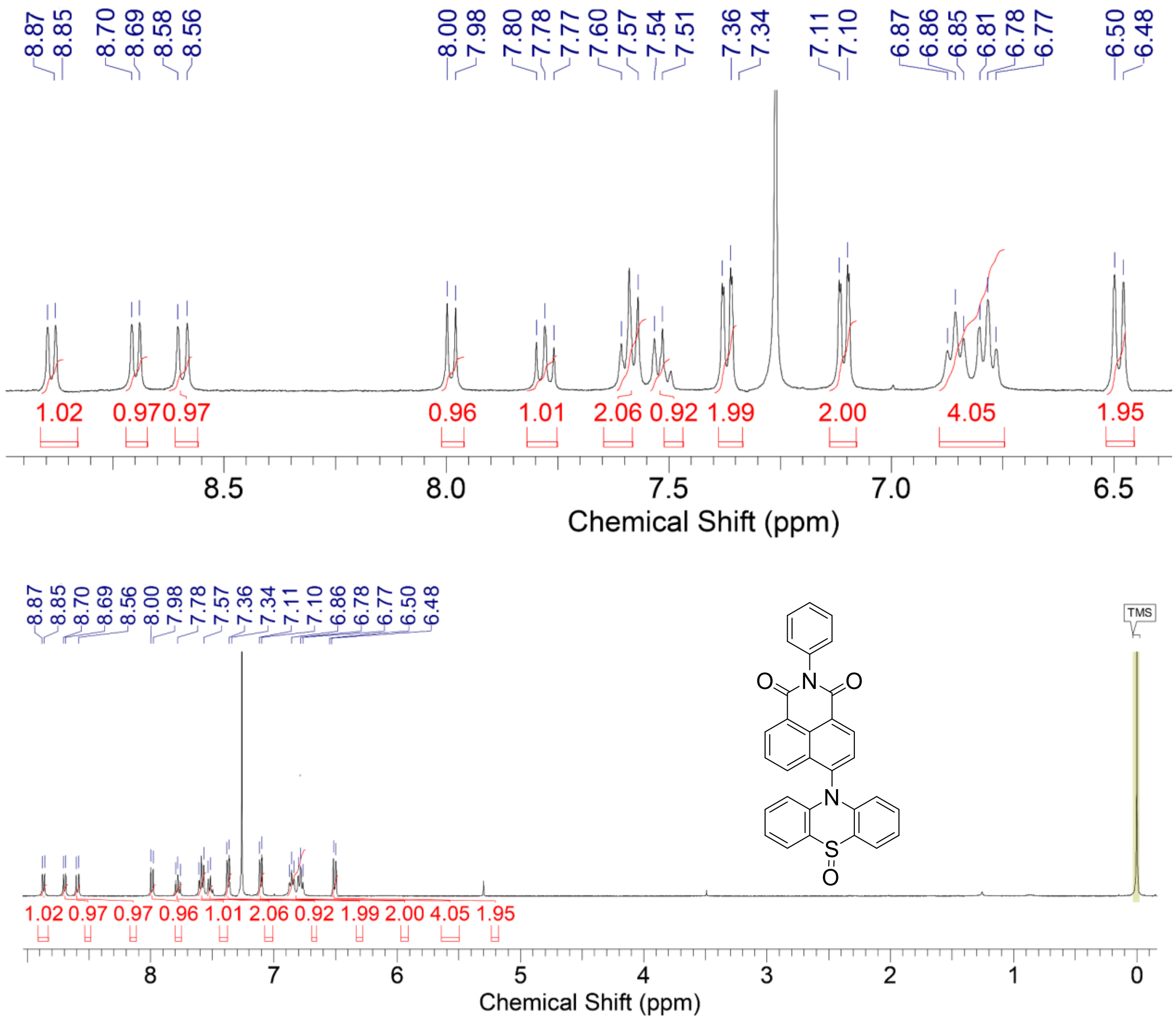

Figure S23. ^{1}H NMR spectrum of **NI-PTZ-Ph-O** in $CDCl_3$ (400 MHz), 25 °C.

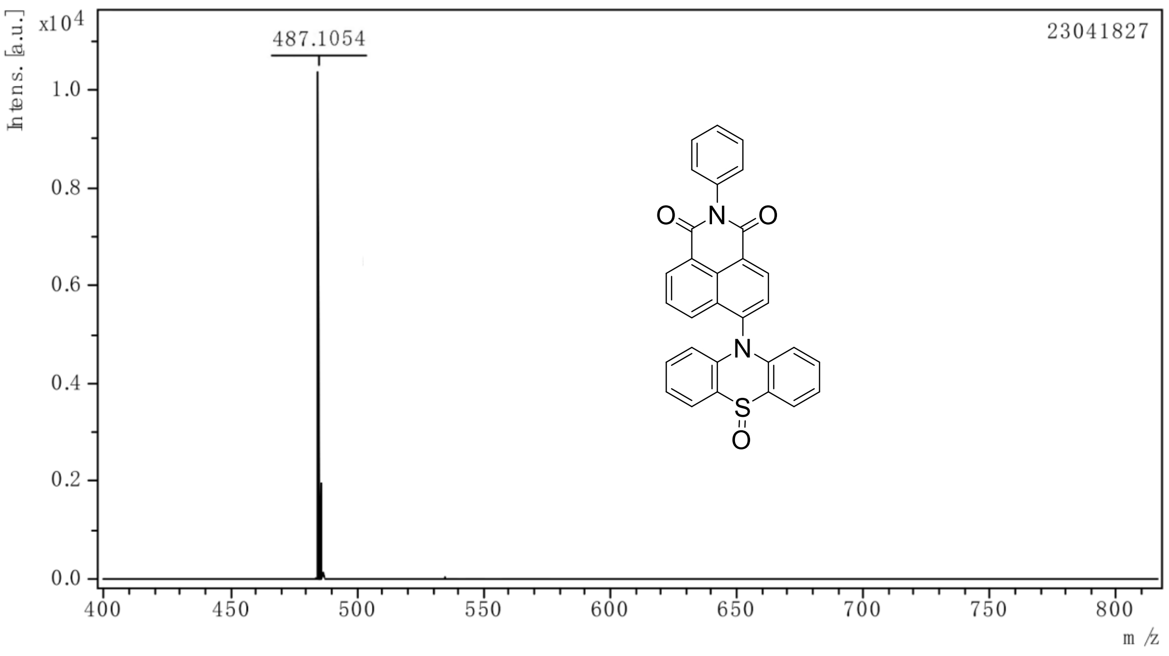

Figure S24. MALDI-HRMS spectrum of **NI-PTZ-Ph-O**, 25 °C.

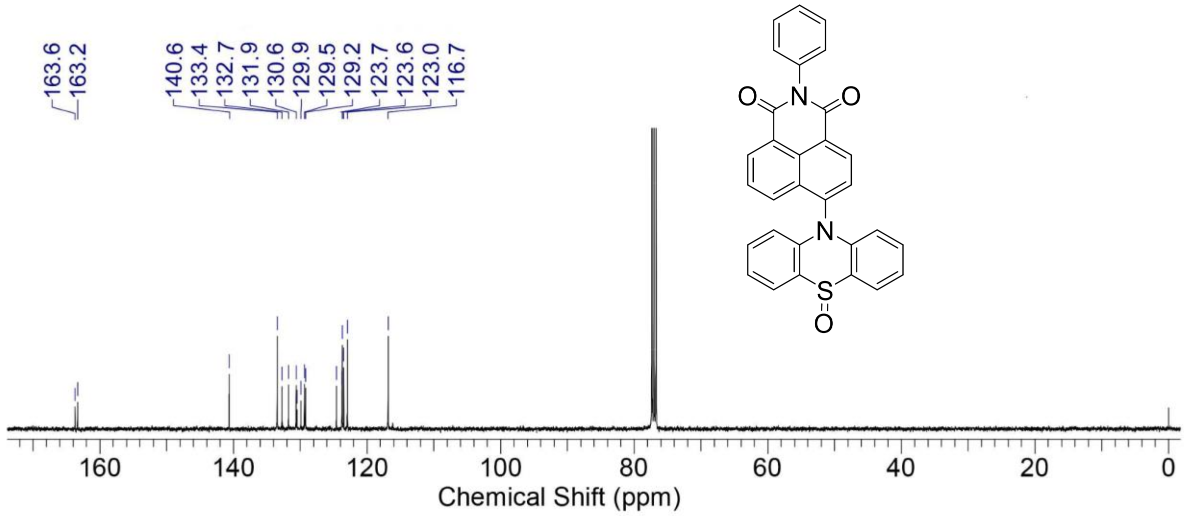

Figure S25. ^{13}C NMR spectrum of **NI-PTZ-Ph-O** in $CDCl_3$ (125 MHz), 25 °C.

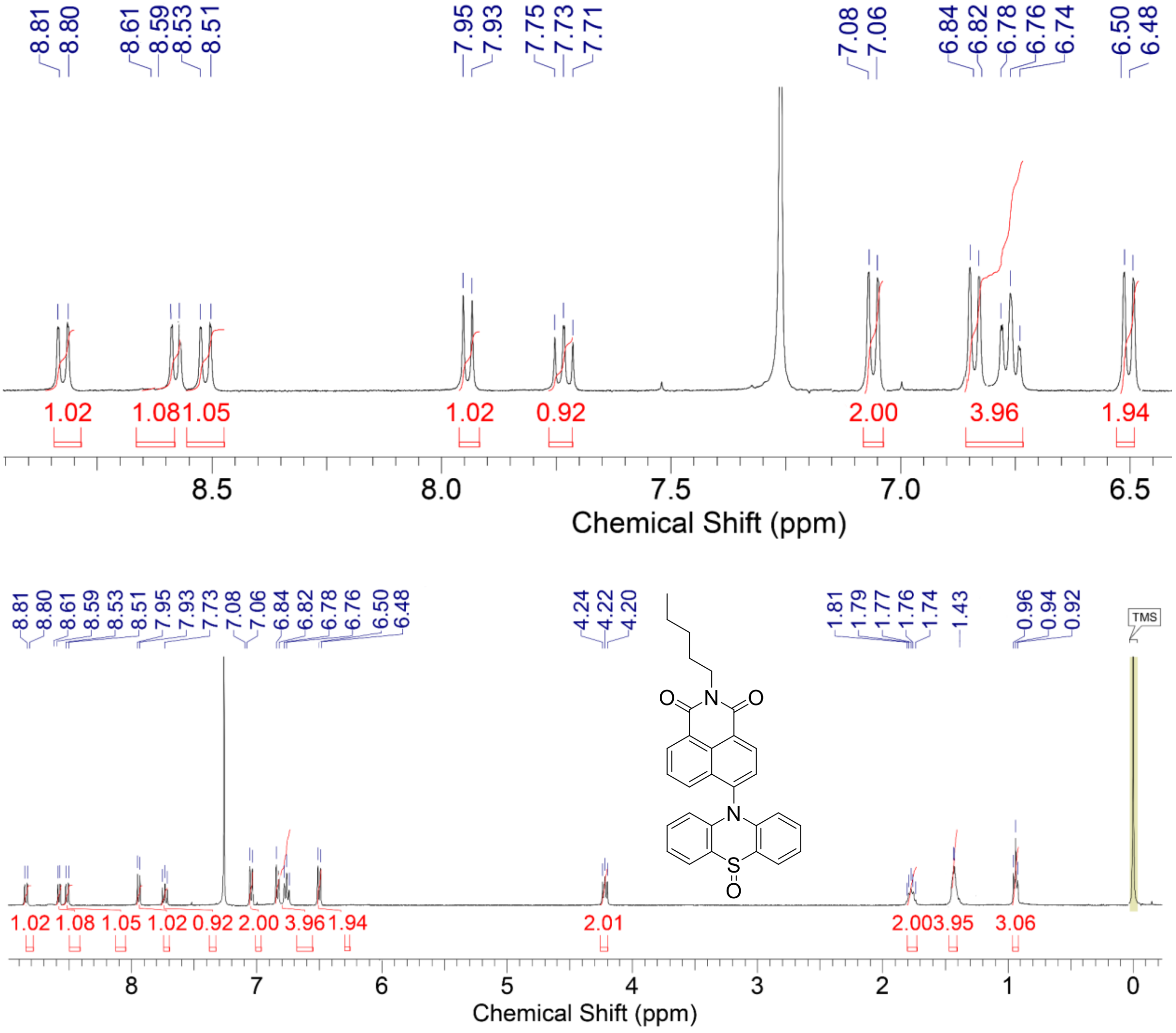

Figure S26. ^{1}H NMR spectrum of **NI-PTZ-C$_5$-O** in $CDCl_3$ (400 MHz), 25 °C.

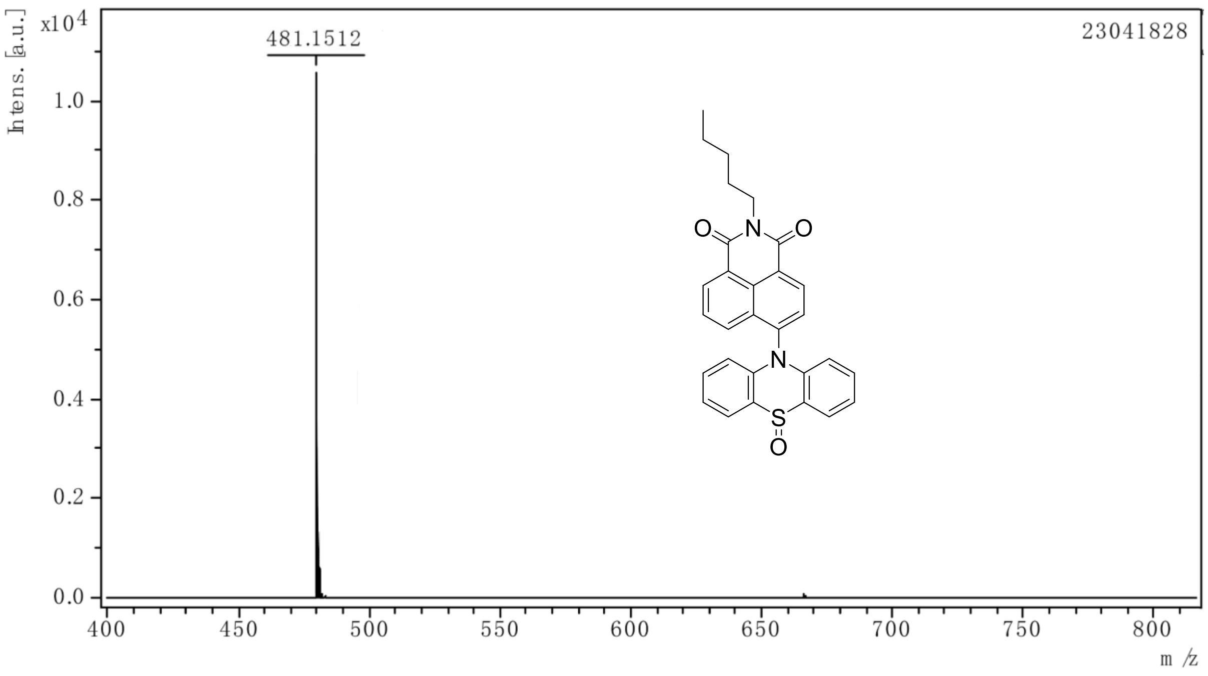

Figure S27. MALDI-HRMS spectrum of **NI-PTZ-C$_5$-O**, 25 °C.

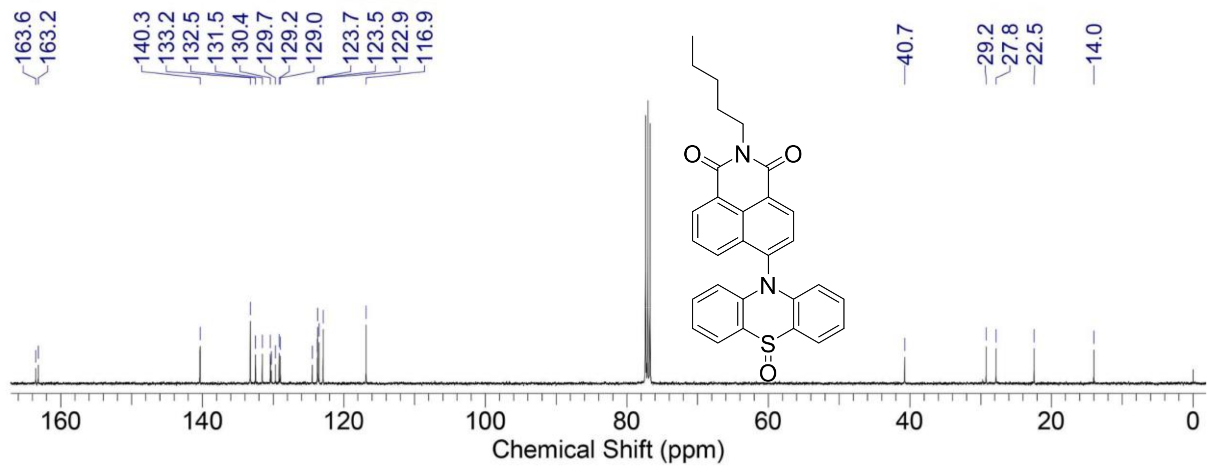

Figure S28. ^{13}C NMR spectrum of **NI-PTZ-C$_5$-O** in $CDCl_3$ (125 MHz), 25 °C.

4. Steady state UV−vis absorption and luminescence spectra

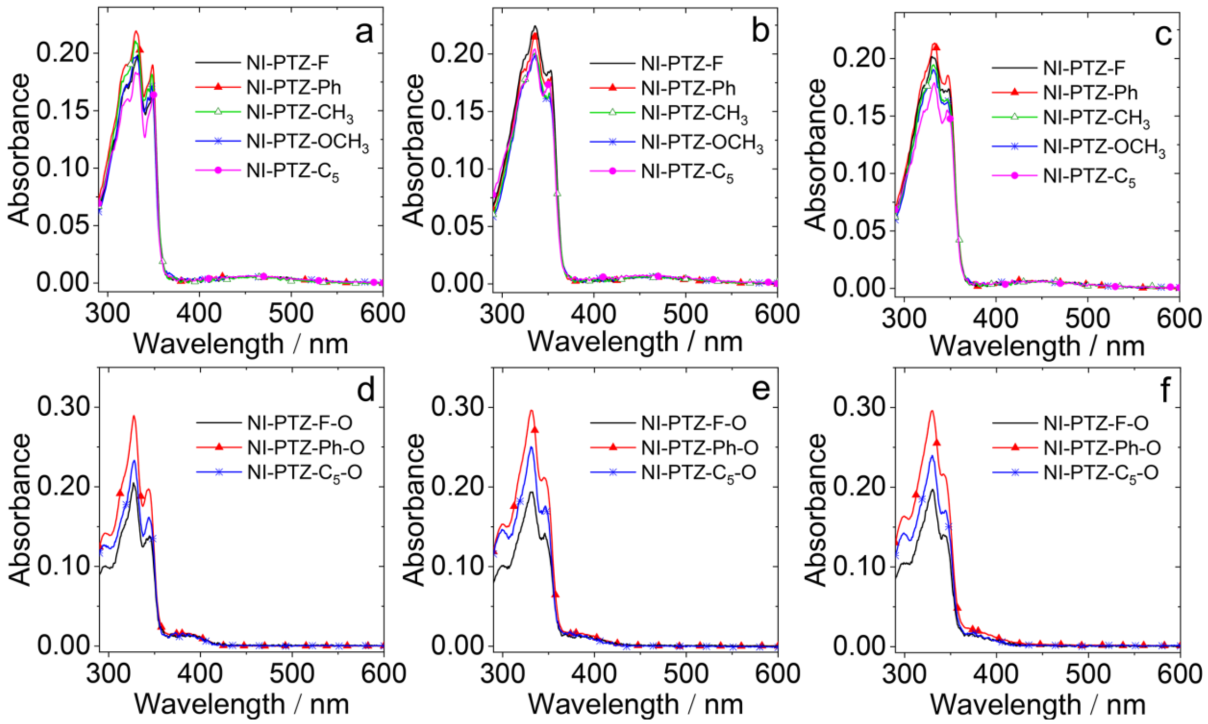

Figure S29. UV–vis absorption spectra of **NI-PTZ-F**; **NI-PTZ-Ph**; **NI-PTZ-CH$_3$**; **NI-PTZ-OCH$_3$** and **NI-PTZ-C$_5$** in (a) cyclohexane (CHX); (b) toluene (TOL); (c) acetonitrile (ACN). **NI-PTZ-F-O**; **NI-PTZ-Ph-O** and **NI-PTZ-C$_5$-O** in (d) CHX; (e) TOL; (f) ACN. c = 1.0×10^{-5} M, 20 °C.

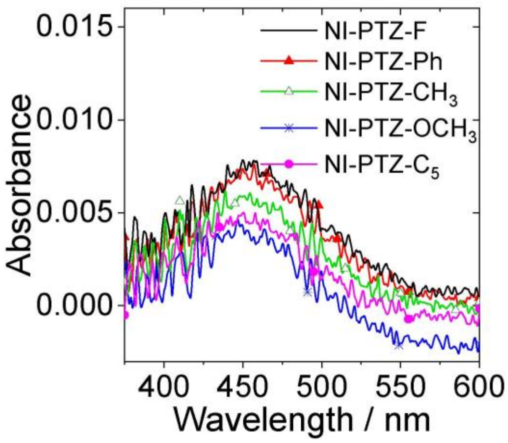

Figure S30. The CT absorption bands locally amplified spectra of **NI-PTZ-F**; **NI-PTZ-Ph**; **NI-PTZ-CH$_3$**; **NI-PTZ-OCH$_3$** and **NI-PTZ-C$_5$** in *n*-hexane (HEX). $c = 1.0 \times 10^{-5}$ M, 20 °C.

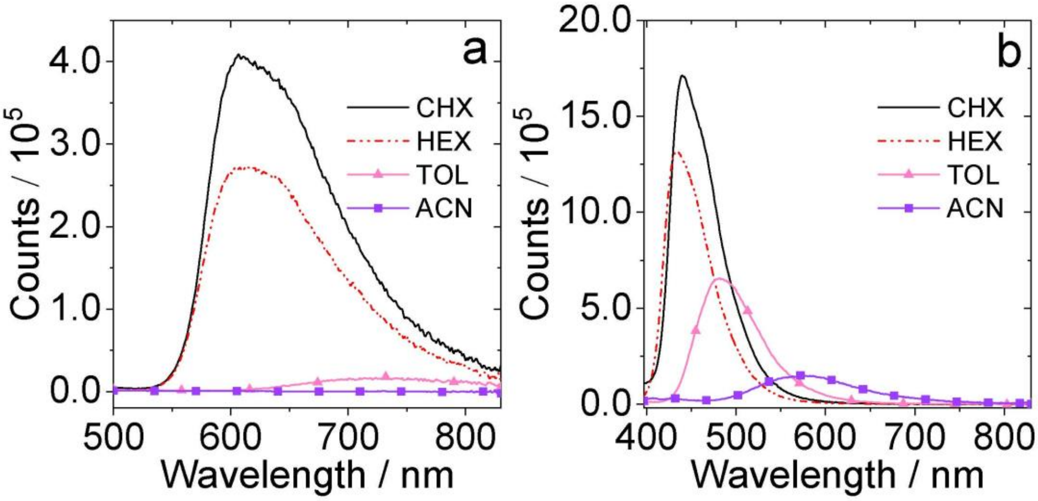

Figure S31. Fluorescence emission spectra of (a) **NI-PTZ-C$_5$** and (b) **NI-PTZ-C$_5$-O** in different solvents. Optically-matched solutions were used, A = 0.107, λ_{ex} = 310 nm, 20 °C.

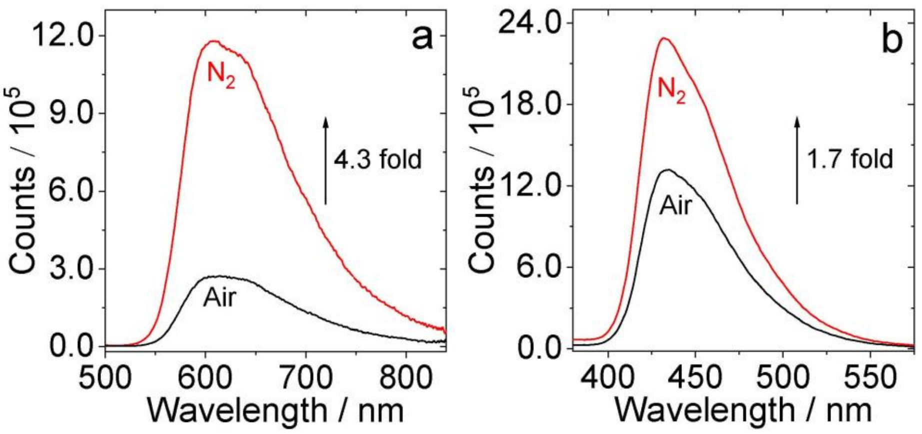

Figure S32. Fluorescence emission spectra of (a) **NI-PTZ-C$_5$**; (b) **NI-PTZ-C$_5$-O** in HEX. Under different atmospheres (N_2, air). Optically-matched solutions were used, A = 0.107, λ_{ex} = 310 nm, 20 °C.

5. Fluorescence lifetimes

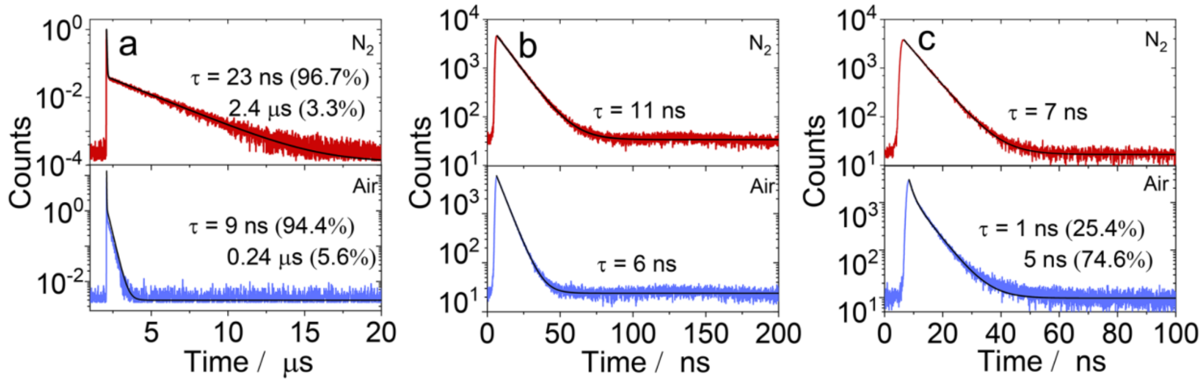

Figure S33. Decay traces of the luminescence of (a) **NI-PTZ-C$_5$** (λ_{em} = 610 nm, c = 1.0 × 10^{-5} M) and (b) **NI-PTZ-C$_5$-O** (λ_{em} = 440 nm, c = 2.0 × 10^{-5} M) in different atmosphere (N_2, Air) in HEX and (c) **NI-PTZ-F-O** (λ_{em} = 440 nm, c = 2.0 × 10^{-5} M) in ACN. Excited with picoseconds pulsed laser for fluorescence band (λ_{ex} = 340 nm), 20 °C.

6. Thermal stability analysis (Thermogravimetry Analysis, TGA)

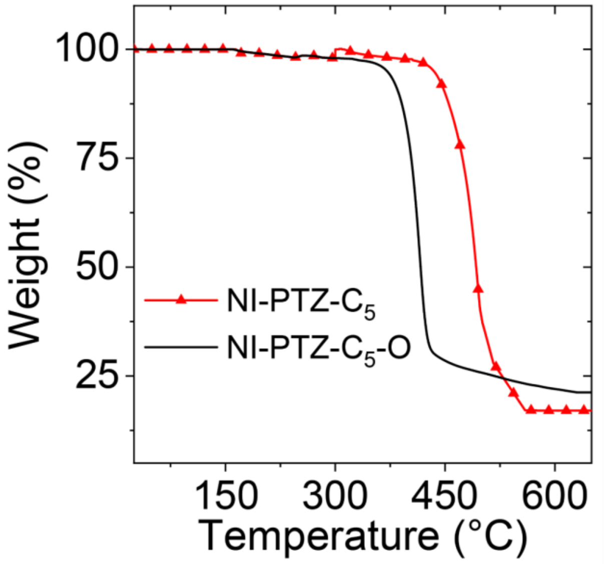

Figure S34. Thermogravimetric analysis curves of **NI-PTZ-C$_5$** and **NI-PTZ-C$_5$-O**. Temperature range: 25–800 °C, heating rate: 10 °C/min in N_2 atmosphere.

7. Femtosecond transient absorption spectroscopy

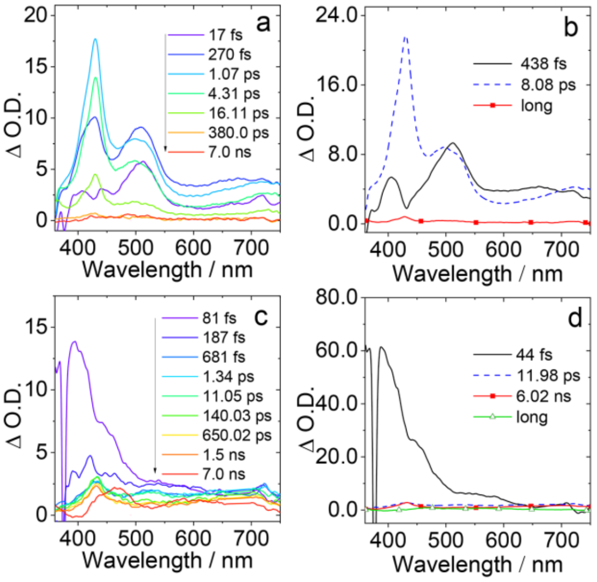

Figure S35. Femtosecond transient absorption spectra of **NI-PTZ-F**. (a) Transient absorption spectra; (b) relative EADS obtained with global analysis in ACN. Femtosecond transient absorption spectra of **NI-PTZ-F-O** (c) Transient absorption spectra; (d) relative EADS obtained with global analysis in HEX. λ_{ex} = 340 nm.

8. Nanosecond transient absorption spectroscopy

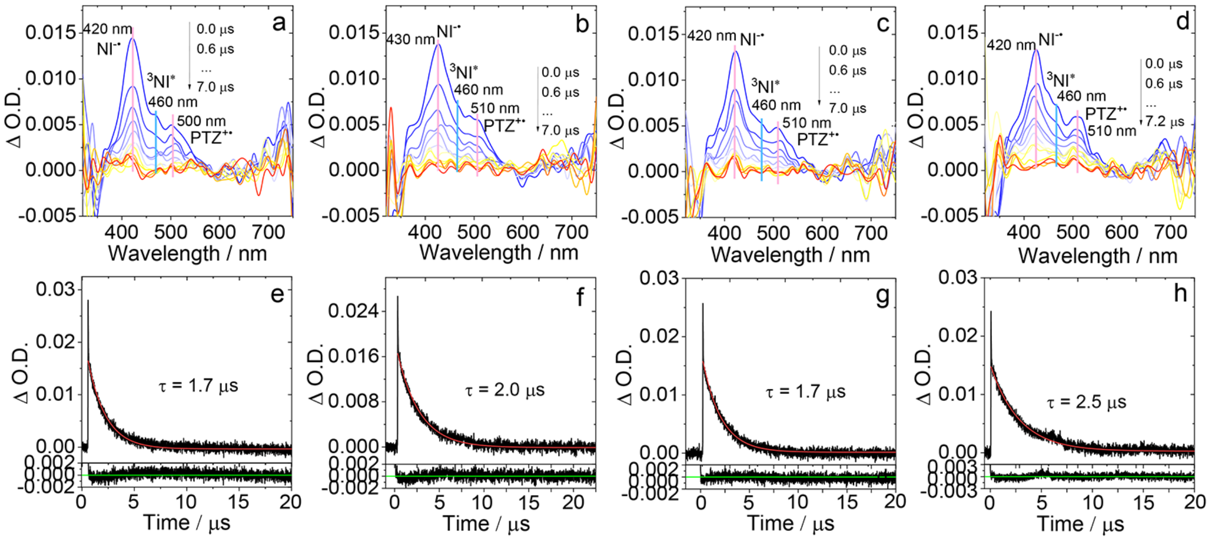

Figure S36. Nanosecond transient absorption spectra of (a)**NI-PTZ-Ph**; (b) **NI-PTZ-CH₃**; (c) **NI-PTZ-OCH₃**; (d) **NI-PTZ-C₅** and decay trace of (e)**NI-PTZ-Ph**; (f) **NI-PTZ-CH₃**; (g) **NI-PTZ-OCH₃**; (h) **NI-PTZ-C₅** in deaerated HEX at 430 nm. c = 2.0 × 10^{-5} M, λ_{ex} = 355 nm.

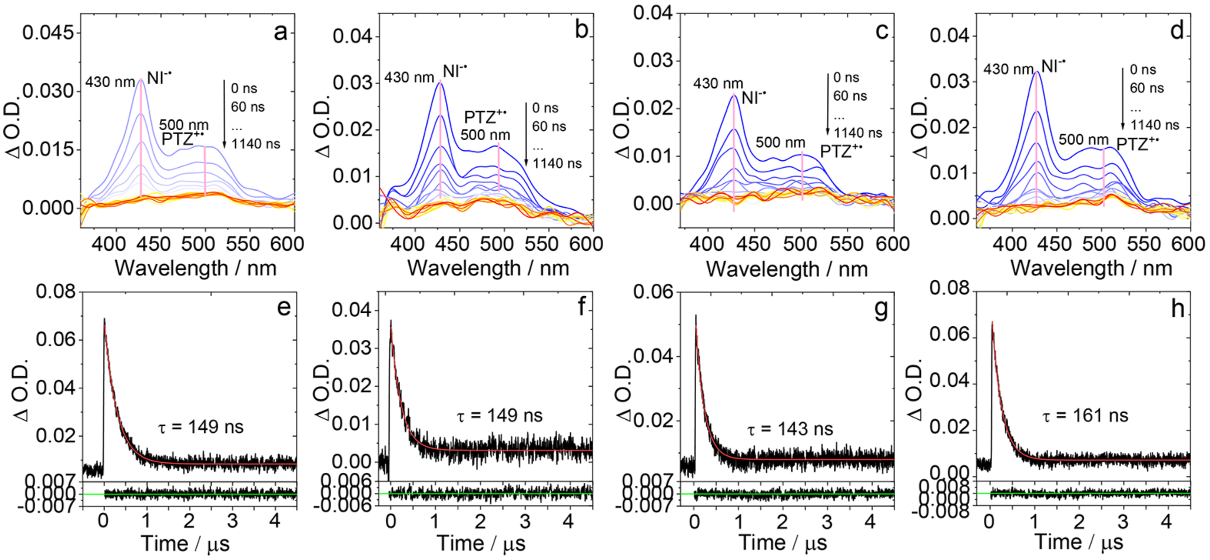

Figure S37. Nanosecond transient absorption (ns-TA) spectra of (a) **NI-PTZ-Ph**; (b) **NI-PTZ-CH₃**; (c) **NI-PTZ-OCH₃**; (d)**NI-PTZ-C₅** and Decay trace of (e) **NI-PTZ-Ph**; (f) **NI-PTZ-CH₃**; (g) **NI-PTZ-OCH₃**; (h)**NI-PTZ-C₅** in deaerated ACN at 430 nm. c = 1.0 × 10^{-4} M, λ_{ex} = 355 nm.

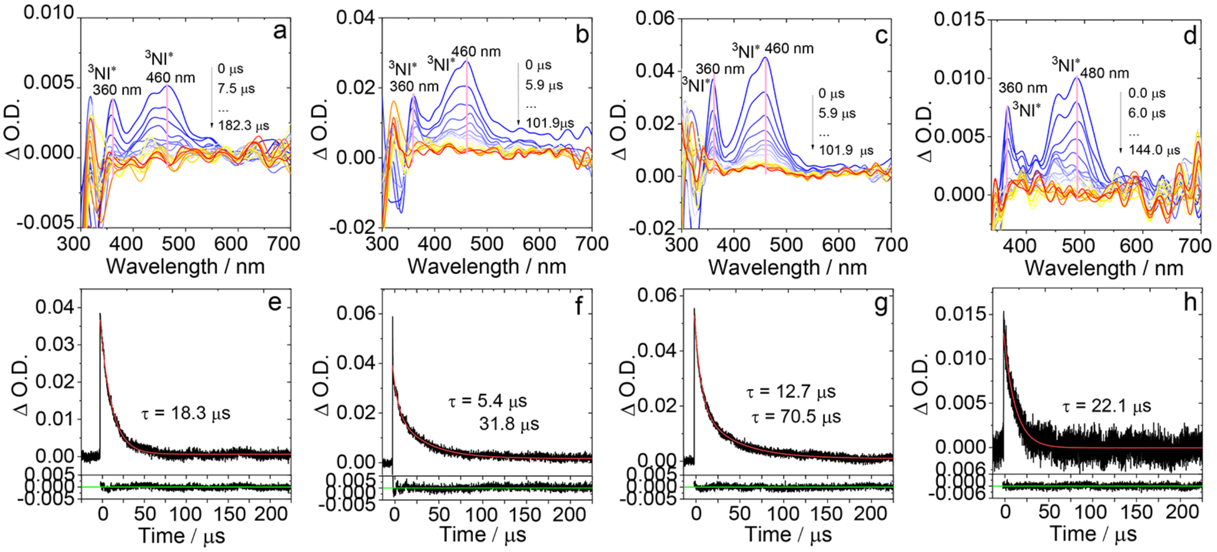

Figure S38. Nanosecond transient absorption spectra of (a) **NI-PTZ-F-O** ($c = 4.0 \times 10^{-5}$ M); (b) **NI-PTZ-Ph-O** ($c = 4.0 \times 10^{-5}$ M); (c) **NI-PTZ-C$_5$-O** ($c = 4.0 \times 10^{-5}$ M) and (d) **F-NI-Br** ($c = 2.0 \times 10^{-5}$ M). The corresponding decay traces are (e) **NI-PTZ-F-O** ($c = 4.0 \times 10^{-5}$ M); (f) **NI-PTZ-Ph-O** ($c = 4.0 \times 10^{-5}$ M); (g) **NI-PTZ-C$_5$-O** ($c = 4.0 \times 10^{-5}$ M) and (h) **F-NI-Br** ($c = 2.0 \times 10^{-5}$ M) in deaerated HEX at 460 nm. λ_{ex} = 355 nm, 20 °C.

9. Theoretical computation

Table S1. Optimized S_1 state geometry of **NI-PTZ-F**; **NI-PTZ-Ph**; **NI-PTZ-CH$_3$**; **NI-PTZ-OCH$_3$**; **NI-PTZ-F-O**; **NI-PTZ-Ph-O** and **NI-PTZ-C$_5$-O**. Calculated at the B3LYP/6-31G(d) level with Gaussian 09W.

Compounds	S_1		
	gas phase	HEX	ACN
**NI-PTZ-F**	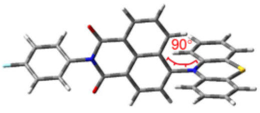	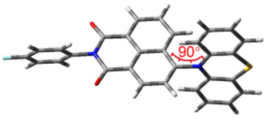	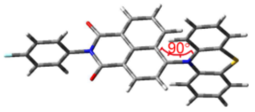
**NI-PTZ-Ph**	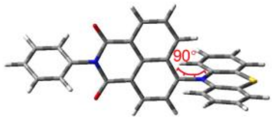	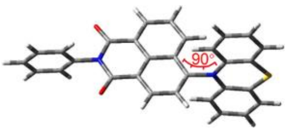	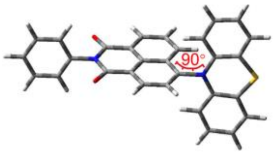
**NI-PTZ-CH$_3$**	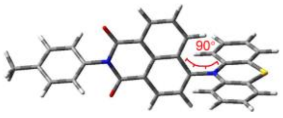	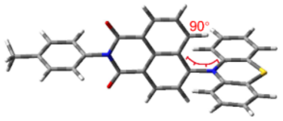	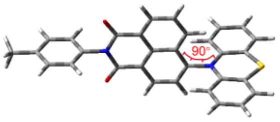
**NI-PTZ-OCH$_3$**	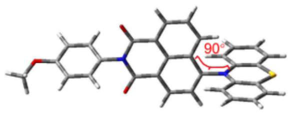	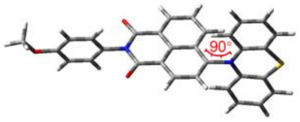	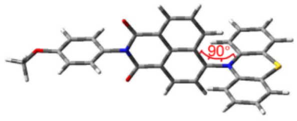
**NI-PTZ-F-O**	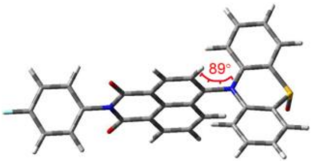	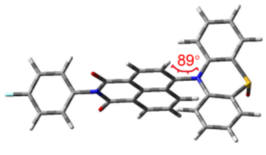	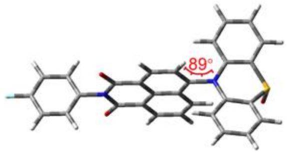
**NI-PTZ-Ph-O**	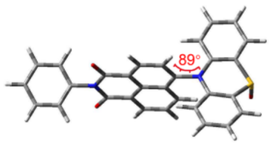	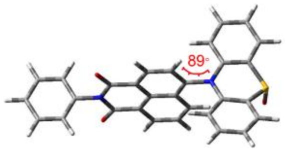	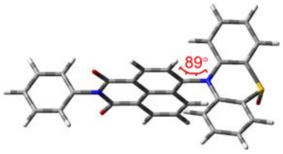
**NI-PTZ-C$_5$-O**	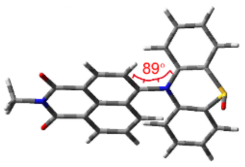	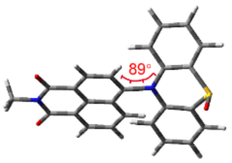	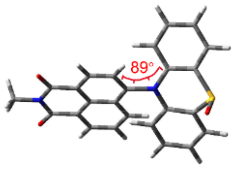

Table S2. Optimized T_1 state geometry of **NI-PTZ-F**; **NI-PTZ-Ph**; **NI-PTZ-CH$_3$**; **NI-PTZ-OCH$_3$**; **NI-PTZ-F-O**; **NI-PTZ-Ph-O** and **NI-PTZ-C$_5$-O**. Calculated at the B3LYP/6-31G(d) level with Gaussian 09W.

Compounds	T_1		
	gas phase	HEX	ACN
**NI-PTZ-F**	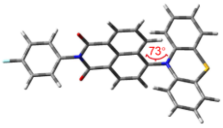	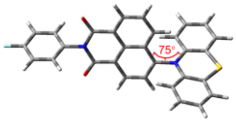	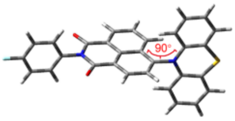
**NI-PTZ-Ph**	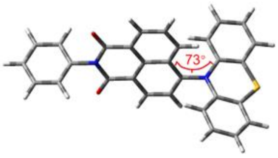	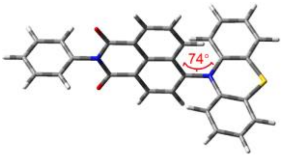	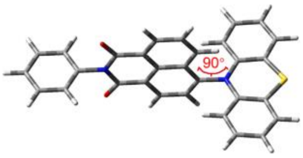
**NI-PTZ-CH$_3$**	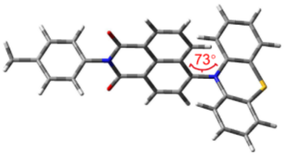	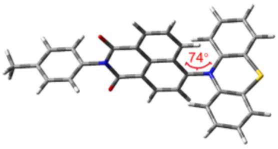	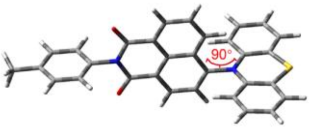
**NI-PTZ-OCH$_3$**	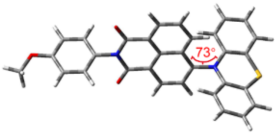	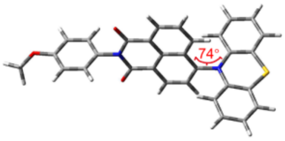	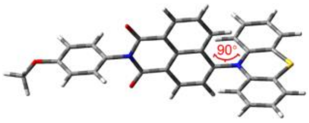
**NI-PTZ-F-O**	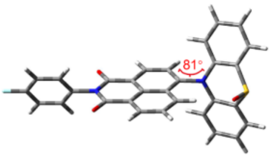	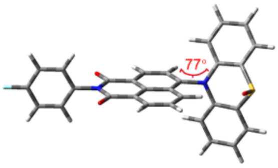	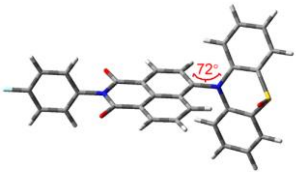
**NI-PTZ-Ph-O**	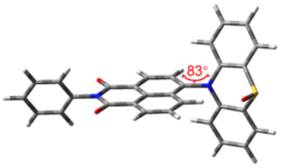	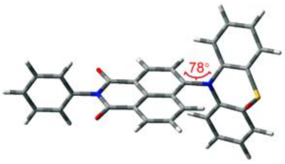	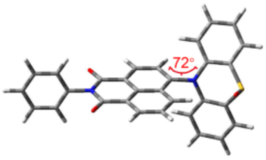
**NI-PTZ-C$_5$-O**	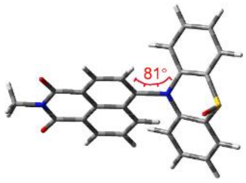	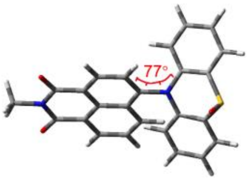	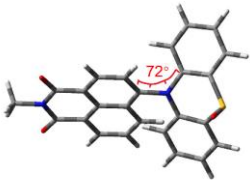

Table S3. Spin density surfaces of the dyads at T_1 state in different solvents of (a) **NI-PTZ-F**; (b) **NI-PTZ-Ph**; (c) **NI-PTZ-CH$_3$**; (d) **NI-PTZ-OCH$_3$**; (e) **NI-PTZ-F-O**; (f) **NI-PTZ-Ph-O**; (g) **NI-PTZ-C$_5$-O**. Calculated at the B3LYP/6-31G(d) level with Gaussian 09W. Isovalues = 0.02.

Compounds	Solvents			
	CHX	TOL	DCM	ACN
**NI-PTZ-F**	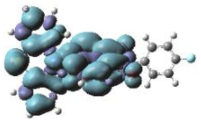	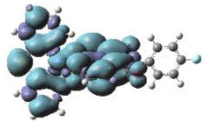	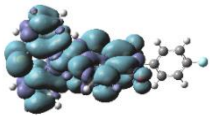	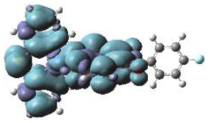
**NI-PTZ-Ph**	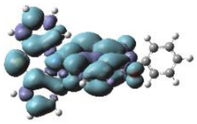	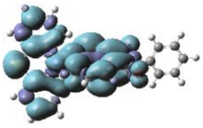	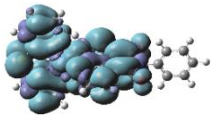	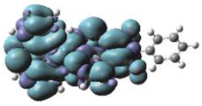
**NI-PTZ-CH$_3$**	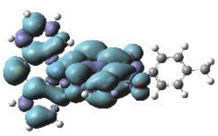	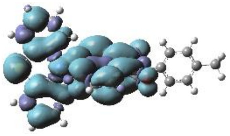	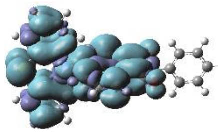	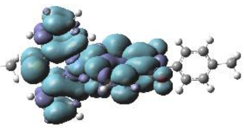
**NI-PTZ-OCH$_3$**	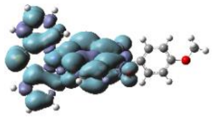	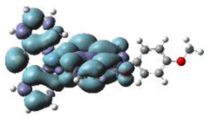	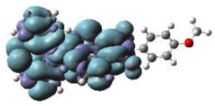	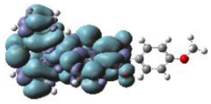
**NI-PTZ-F-O**	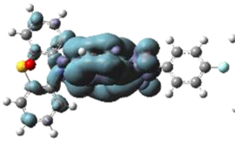	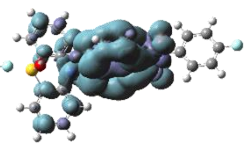	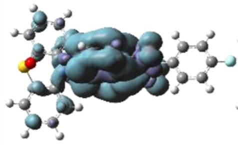	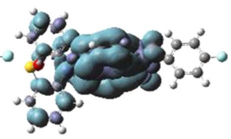
**NI-PTZ-Ph-O**	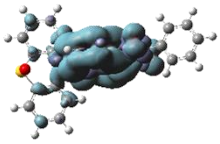	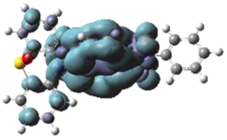	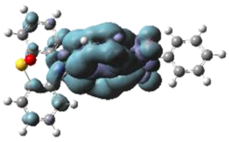	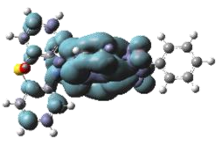
**NI-PTZ-C$_5$-O**	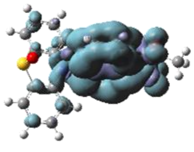	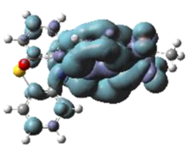	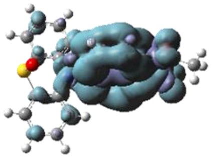	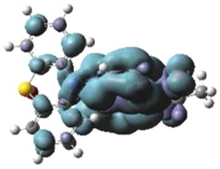

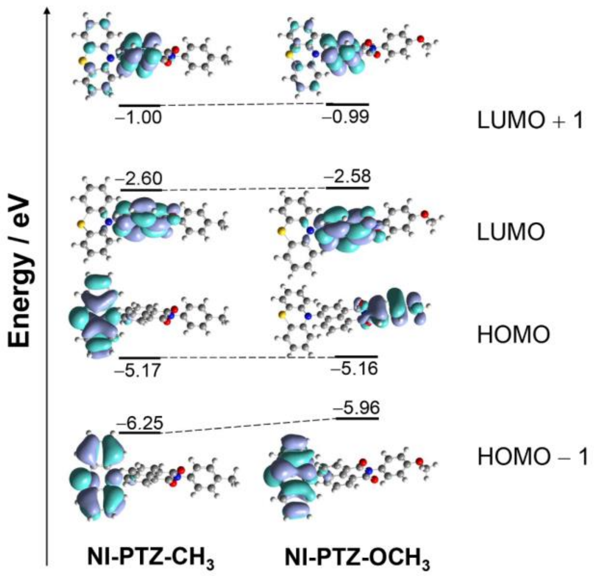

Figure S39. Selected frontier molecular orbitals of **NI-PTZ-CH$_3$** and **NI-PTZ-OCH$_3$** calculated by DFT at the B3LYP/6-31G(d) level with Gaussian 09W, based on the optimized ground state geometries, respectively. Isovalues = 0.02.

10. Coordinates of the optimized geometries of the compounds

Optimized ground state geometries, based on the DFT-B3LYP/6-31(G) level.

NI-PTZ-F

0 1

C	3.25043	-0.44951	-1.15381
C	3.58498	-0.67388	1.30216
C	1.78878	-0.31154	-0.92403
O	3.74425	-0.39252	-2.27263
C	2.12039	-0.53128	1.50107
O	4.36344	-0.81531	2.23561
C	0.94692	-0.13691	-2.00653
C	1.25745	-0.35596	0.38925
C	1.60309	-0.57028	2.78325
C	-0.44215	-0.00589	-1.81212

H	1.3766	-0.10399	-3.00215
C	-0.14988	-0.22189	0.59978
C	0.21506	-0.43918	2.99391
H	2.28722	-0.70412	3.61467
C	-0.99163	-0.04644	-0.5418
H	-1.09837	0.12948	-2.66656
C	-0.64623	-0.2689	1.92929
C	-2.5782	1.23999	2.69677
C	-2.92282	-1.20715	2.53378
C	-2.0283	2.34886	2.0368
C	-3.66839	1.45311	3.5619
C	-4.04599	-1.22188	3.38285
C	-2.68698	-2.32976	1.72654
C	-2.54898	3.628	2.2359
H	-1.18252	2.21643	1.37314
C	-4.21125	2.72864	3.72239
C	-4.92262	-2.30767	3.38538
H	-1.82432	-2.35146	1.07187
C	-3.54401	-3.43021	1.76662
C	-3.6469	3.82516	3.0705
H	-2.09387	4.46913	1.72061
H	-5.06653	2.85792	4.37964
C	-4.67005	-3.42319	2.58688
H	-5.7929	-2.2801	4.03501
H	-3.32822	-4.29013	1.13881
H	-4.06193	4.81788	3.21649
H	-5.34549	-4.27308	2.60951
N	-2.05935	-0.07478	2.5235
S	-4.28821	0.10453	4.54776
H	-2.04928	0.05386	-0.41449
H	-0.17456	-0.47207	3.9899

C	5.00853	-1.06278	-0.37638
C	5.1944	-2.44391	-0.4354
C	6.03576	-0.20327	-0.76684
C	6.40799	-2.96589	-0.88478
H	4.38469	-3.1217	-0.12839
C	7.249	-0.72505	-1.21554
H	5.88893	0.8855	-0.72041
C	7.43515	-2.10649	-1.27416
H	6.55442	-4.05477	-0.93155
H	8.05879	-0.04767	-1.523
N	4.0522	-0.65109	-0.02225
F	8.60921	-2.61113	-1.70939

NI-PTZ-Ph

0 1

C	3.25043	-0.44951	-1.15381
C	3.58498	-0.67388	1.30216
C	1.78878	-0.31154	-0.92403
O	3.74425	-0.39252	-2.27263
C	2.12039	-0.53128	1.50107
O	4.36344	-0.81531	2.23561
C	0.94692	-0.13691	-2.00653
C	1.25745	-0.35596	0.38925
C	1.60309	-0.57028	2.78325
C	-0.44215	-0.00589	-1.81212
H	1.3766	-0.10399	-3.00215
C	-0.14988	-0.22189	0.59978
C	0.21506	-0.43918	2.99391
H	2.28722	-0.70412	3.61467
C	-0.99163	-0.04644	-0.5418
H	-1.09837	0.12948	-2.66656

C	-0.64623	-0.2689	1.92929
C	-2.5782	1.23999	2.69677
C	-2.92282	-1.20715	2.53378
C	-2.0283	2.34886	2.0368
C	-3.66839	1.45311	3.5619
C	-4.04599	-1.22188	3.38285
C	-2.68698	-2.32976	1.72654
C	-2.54898	3.628	2.2359
H	-1.18252	2.21643	1.37314
C	-4.21125	2.72864	3.72239
C	-4.92262	-2.30767	3.38538
H	-1.82432	-2.35146	1.07187
C	-3.54401	-3.43021	1.76662
C	-3.6469	3.82516	3.0705
H	-2.09387	4.46913	1.72061
H	-5.06653	2.85792	4.37964
C	-4.67005	-3.42319	2.58688
H	-5.7929	-2.2801	4.03501
H	-3.32822	-4.29013	1.13881
H	-4.06193	4.81788	3.21649
H	-5.34549	-4.27308	2.60951
N	-2.05935	-0.07478	2.5235
S	-4.28821	0.10453	4.54776
H	-2.04928	0.05386	-0.41449
H	-0.17456	-0.47207	3.9899
C	5.00853	-1.06278	-0.37638
C	5.1944	-2.44391	-0.4354
C	6.03576	-0.20327	-0.76684
C	6.40799	-2.96589	-0.88478
H	4.38469	-3.1217	-0.12839
C	7.249	-0.72505	-1.21554

H	5.88893	0.8855	-0.72041
C	7.43515	-2.10649	-1.27416
H	6.55442	-4.05477	-0.93155
H	8.05879	-0.04767	-1.523
H	8.39149	-2.51755	-1.62868
N	4.0522	-0.65109	-0.02225

NI-PTZ-CH$_3$

0 1

C	3.25043	-0.44951	-1.15381
C	3.58498	-0.67388	1.30216
C	1.78878	-0.31154	-0.92403
O	3.74425	-0.39252	-2.27263
C	2.12039	-0.53128	1.50107
O	4.36344	-0.81531	2.23561
C	0.94692	-0.13691	-2.00653
C	1.25745	-0.35596	0.38925
C	1.60309	-0.57028	2.78325
C	-0.44215	-0.00589	-1.81212
H	1.3766	-0.10399	-3.00215
C	-0.14988	-0.22189	0.59978
C	0.21506	-0.43918	2.99391
H	2.28722	-0.70412	3.61467
C	-0.99163	-0.04644	-0.5418
H	-1.09837	0.12948	-2.66656
C	-0.64623	-0.2689	1.92929
C	-2.5782	1.23999	2.69677
C	-2.92282	-1.20715	2.53378
C	-2.0283	2.34886	2.0368
C	-3.66839	1.45311	3.5619
C	-4.04599	-1.22188	3.38285

C	-2.68698	-2.32976	1.72654
C	-2.54898	3.628	2.2359
H	-1.18252	2.21643	1.37314
C	-4.21125	2.72864	3.72239
C	-4.92262	-2.30767	3.38538
H	-1.82432	-2.35146	1.07187
C	-3.54401	-3.43021	1.76662
C	-3.6469	3.82516	3.0705
H	-2.09387	4.46913	1.72061
H	-5.06653	2.85792	4.37964
C	-4.67005	-3.42319	2.58688
H	-5.7929	-2.2801	4.03501
H	-3.32822	-4.29013	1.13881
H	-4.06193	4.81788	3.21649
H	-5.34549	-4.27308	2.60951
N	-2.05935	-0.07478	2.5235
S	-4.28821	0.10453	4.54776
H	-2.04928	0.05386	-0.41449
H	-0.17456	-0.47207	3.9899
C	5.00853	-1.06278	-0.37638
C	5.1944	-2.44391	-0.4354
C	6.03576	-0.20327	-0.76684
C	6.40799	-2.96589	-0.88478
H	4.38469	-3.1217	-0.12839
C	7.249	-0.72505	-1.21554
H	5.88893	0.8855	-0.72041
H	6.55296	-4.0548	-0.93548
H	8.06025	-0.04764	-1.51907
N	4.0522	-0.65109	-0.02225
C	8.44458	-2.46072	-1.29574
H	8.95908	-2.12533	-0.41955

H	8.4467	-3.53037	-1.32311
H	8.9374	-2.08069	-2.16615
C	7.43515	-2.10649	-1.27416

NI-PTZ-OCH$_3$

0 1

C	3.25043	-0.44951	-1.15381
C	3.58498	-0.67388	1.30216
C	1.78878	-0.31154	-0.92403
O	3.74425	-0.39252	-2.27263
C	2.12039	-0.53128	1.50107
O	4.36344	-0.81531	2.23561
C	0.94692	-0.13691	-2.00653
C	1.25745	-0.35596	0.38925
C	1.60309	-0.57028	2.78325
C	-0.44215	-0.00589	-1.81212
H	1.3766	-0.10399	-3.00215
C	-0.14988	-0.22189	0.59978
C	0.21506	-0.43918	2.99391
H	2.28722	-0.70412	3.61467
C	-0.99163	-0.04644	-0.5418
H	-1.09837	0.12948	-2.66656
C	-0.64623	-0.2689	1.92929
C	-2.5782	1.23999	2.69677
C	-2.92282	-1.20715	2.53378
C	-2.0283	2.34886	2.0368
C	-3.66839	1.45311	3.5619
C	-4.04599	-1.22188	3.38285
C	-2.68698	-2.32976	1.72654
C	-2.54898	3.628	2.2359
H	-1.18252	2.21643	1.37314

C	-4.21125	2.72864	3.72239
C	-4.92262	-2.30767	3.38538
H	-1.82432	-2.35146	1.07187
C	-3.54401	-3.43021	1.76662
C	-3.6469	3.82516	3.0705
H	-2.09387	4.46913	1.72061
H	-5.06653	2.85792	4.37964
C	-4.67005	-3.42319	2.58688
H	-5.7929	-2.2801	4.03501
H	-3.32822	-4.29013	1.13881
H	-4.06193	4.81788	3.21649
H	-5.34549	-4.27308	2.60951
N	-2.05935	-0.07478	2.5235
S	-4.28821	0.10453	4.54776
H	-2.04928	0.05386	-0.41449
H	-0.17456	-0.47207	3.9899
C	5.00853	-1.06278	-0.37638
C	5.1944	-2.44391	-0.4354
C	6.03576	-0.20327	-0.76684
C	6.40799	-2.96589	-0.88478
H	4.38469	-3.1217	-0.12839
C	7.249	-0.72505	-1.21554
H	5.88893	0.8855	-0.72041
H	6.55296	-4.0548	-0.93548
H	8.06025	-0.04764	-1.51907
N	4.0522	-0.65109	-0.02225
C	7.43515	-2.10649	-1.27416
C	9.29695	-2.90693	-1.96161
H	9.92818	-3.11289	-1.12254
H	9.1177	-3.81185	-2.50371
H	9.77589	-2.19626	-2.60229

O	8.36605	-2.50671	-1.61788

NI-PTZ-C_5

0 1

N	4.0522	-0.65109	-0.02225
C	3.25043	-0.44951	-1.15381
C	3.58498	-0.67388	1.30216
C	1.78878	-0.31154	-0.92403
O	3.74358	-0.39689	-2.27304
C	2.12039	-0.53128	1.50107
O	4.36409	-0.81093	2.23583
C	0.94692	-0.13691	-2.00653
C	1.25745	-0.35596	0.38925
C	1.60309	-0.57028	2.78325
C	-0.44215	-0.00589	-1.81212
H	1.3766	-0.10399	-3.00215
C	-0.14988	-0.22189	0.59978
C	0.21506	-0.43918	2.99391
H	2.28722	-0.70412	3.61467
C	-0.99163	-0.04644	-0.5418
H	-1.09837	0.12948	-2.66656
C	-0.64623	-0.2689	1.92929
C	-2.5782	1.23999	2.69677
C	-2.92282	-1.20715	2.53378
C	-2.0283	2.34886	2.0368
C	-3.66839	1.45311	3.5619
C	-4.04599	-1.22188	3.38285
C	-2.68698	-2.32976	1.72654
C	-2.54898	3.628	2.2359
H	-1.18252	2.21643	1.37314
C	-4.21125	2.72864	3.72239

C	-4.92262	-2.30767	3.38538
H	-1.82432	-2.35146	1.07187
C	-3.54401	-3.43021	1.76662
C	-3.6469	3.82516	3.0705
H	-2.09387	4.46913	1.72061
H	-5.06653	2.85792	4.37964
C	-4.67005	-3.42319	2.58688
H	-5.7929	-2.2801	4.03501
H	-3.32822	-4.29013	1.13881
H	-4.06193	4.81788	3.21649
H	-5.34549	-4.27308	2.60951
N	-2.05935	-0.07478	2.5235
S	-4.28821	0.10453	4.54776
C	5.50426	-0.8052	-0.23469
H	5.9145	-1.42067	0.53848
H	5.67894	-1.26341	-1.18571
H	5.97228	0.15672	-0.2109
H	-2.04928	0.05386	-0.41449
H	-0.17456	-0.47207	3.9899

NI-PTZ-F-O

0 1

C	-10.32216	-0.56994	-2.05175
C	-10.28176	0.81095	-1.86886
C	-9.50737	1.35936	-0.84513
C	-8.76467	0.53265	0.01219
C	-8.80444	-0.86737	-0.17719
C	-9.57768	-1.41431	-1.21758
C	-7.98713	1.05893	1.05558
C	-7.26603	0.21497	1.9018
C	-7.30927	-1.16539	1.71531

C	-8.0668	-1.71287	0.67157
C	-8.15077	-3.20843	0.48324
N	-8.42906	-3.57991	-0.90616
C	-9.64335	-2.91346	-1.38302
O	-8.005	-4.00805	1.3769
O	-10.58977	-3.4972	-1.85503
C	-7.67487	-4.41626	-1.64151
C	-6.53504	-4.99787	-1.08652
C	-5.75284	-5.86513	-1.84921
C	-6.11058	-6.1508	-3.16675
C	-7.25044	-5.56928	-3.72171
C	-8.03261	-4.70193	-2.95905
N	-7.93939	2.31182	1.23093
C	-9.14759	2.95002	1.56353
C	-9.20629	4.30892	1.91593
C	-6.46769	4.28082	1.37133
C	-6.68349	2.92472	1.07343
C	-5.18149	4.82682	1.23803
C	-4.10989	4.03412	0.82683
C	-4.31369	2.68662	0.55143
C	-5.58989	2.13542	0.68113
C	-10.32359	2.18392	1.62263
C	-11.54119	2.76072	1.98873
C	-11.59739	4.11092	2.31593
C	-10.43459	4.88072	2.28273
F	-5.37035	-6.97153	-3.88845
H	-10.94081	-0.9986	-2.85395
H	-10.86108	1.46989	-2.53233
H	-9.47889	2.45052	-0.70889
H	-6.66258	0.64018	2.7173
H	-6.74666	-1.82747	2.38994

H	-6.25299	-4.77264	-0.04743
H	-4.85387	-6.32376	-1.4116
H	-7.53257	-5.79463	-4.76075
H	-8.93154	-4.24324	-3.39669
H	-5.01676	5.89147	1.46026
H	-3.10707	4.47358	0.72082
H	-3.47097	2.05594	0.23194
H	-5.74131	1.066	0.47277
H	-10.28451	1.11251	1.37655
H	-12.45434	2.14812	2.01826
H	-12.55556	4.5705	2.60001
H	-10.48072	5.94753	2.54689
S	-7.77189	5.37142	1.91563
O	-6.62728	6.04414	1.21307

NI-PTZ-Ph-O

0 1

C	-10.32216	-0.57252	-2.05402
C	-10.28176	0.80837	-1.87113
C	-9.50737	1.35678	-0.8474
C	-8.76467	0.53007	0.00992
C	-8.80444	-0.86995	-0.17946
C	-9.57768	-1.41689	-1.21985
C	-7.98713	1.05635	1.05331
C	-7.26603	0.21238	1.89953
C	-7.30927	-1.16797	1.71304
C	-8.0668	-1.71545	0.66931
C	-8.15077	-3.21101	0.48097
N	-8.42906	-3.58249	-0.90843
C	-9.64335	-2.91604	-1.38529
O	-8.005	-4.01063	1.37463

O	-10.58977	-3.49978	-1.8573
C	-7.67487	-4.41884	-1.64378
C	-6.53504	-5.00045	-1.08879
C	-5.75284	-5.86771	-1.85148
C	-6.11058	-6.15338	-3.16902
C	-7.25044	-5.57186	-3.72398
C	-8.03261	-4.70451	-2.96132
N	-7.93939	2.30924	1.22866
C	-9.14759	2.94744	1.56126
C	-9.20629	4.30634	1.91366
C	-6.46769	4.27824	1.36906
C	-6.68349	2.92214	1.07116
C	-5.18149	4.82424	1.23576
C	-4.10989	4.03154	0.82456
C	-4.31369	2.68404	0.54916
C	-5.58989	2.13284	0.67886
C	-10.32359	2.18134	1.62036
C	-11.54119	2.75814	1.98646
C	-11.59739	4.10834	2.31366
C	-10.43459	4.87814	2.28046
H	-10.94081	-1.00118	-2.85622
H	-10.86108	1.46731	-2.5346
H	-9.47889	2.44793	-0.71116
H	-6.66258	0.6376	2.71503
H	-6.74666	-1.83005	2.38767
H	-6.25299	-4.77522	-0.0497
H	-4.85387	-6.32634	-1.41387
H	-5.49373	-6.83732	-3.77044
H	-7.53257	-5.79721	-4.76302
H	-8.93154	-4.24582	-3.39896
H	-5.01676	5.88889	1.45799

H	-3.10707	4.471	0.71855
H	-3.47097	2.05336	0.22967
H	-5.74131	1.06342	0.4705
H	-10.28451	1.10993	1.37428
H	-12.45434	2.14554	2.01599
H	-12.55556	4.56792	2.59774
H	-10.48072	5.94495	2.54462
S	-7.77189	5.36884	1.91336
O	-6.62728	6.04156	1.21081

NI-PTZ-C_5-O

0 1

C	-10.32216	-0.17624	-1.82567
C	-10.28176	1.20465	-1.64278
C	-9.50737	1.75305	-0.61904
C	-8.76467	0.92635	0.23827
C	-8.80444	-0.47367	0.04889
C	-9.57768	-1.02061	-0.9915
C	-7.98713	1.45263	1.28166
C	-7.26603	0.60866	2.12788
C	-7.30927	-0.77169	1.94139
C	-8.0668	-1.31917	0.89766
C	-8.15077	-2.81473	0.70932
C	-9.64335	-2.51976	-1.15694
O	-8.00821	-3.61728	1.60302
O	-10.58853	-3.10018	-1.63146
N	-7.93939	2.70552	1.45701
C	-9.14759	3.34372	1.78961
C	-9.20629	4.70262	2.14201
C	-6.46769	4.67452	1.59741
C	-6.68349	3.31842	1.29951

C	-5.18149	5.22052	1.46411
C	-4.10989	4.42782	1.05291
C	-4.31369	3.08032	0.77751
C	-5.58989	2.52912	0.90721
C	-10.32359	2.57762	1.84871
C	-11.54119	3.15442	2.21481
C	-11.59739	4.50462	2.54201
C	-10.43459	5.27442	2.50881
H	-10.94081	-0.60491	-2.62787
H	-10.86108	1.86359	-2.30624
H	-9.47889	2.84421	-0.4828
H	-6.66258	1.03388	2.94338
H	-6.74666	-1.43377	2.61602
H	-5.01676	6.28517	1.68634
H	-3.10707	4.86727	0.9469
H	-3.47097	2.44964	0.45802
H	-5.74131	1.4597	0.69885
H	-10.28451	1.50621	1.60263
H	-12.45434	2.54182	2.24434
H	-12.55556	4.9642	2.82609
H	-10.48072	6.34123	2.77297
S	-7.77189	5.76512	2.14171
O	-6.62728	6.43783	1.43916
C	-8.42906	-3.18621	-0.68008
C	-7.26333	-2.82377	-1.55212
H	-8.61568	-4.28732	-0.70697
H	-7.45554	-3.11895	-2.61217
H	-7.07697	-1.72258	-1.52492
H	-6.33603	-3.34317	-1.20802

References

[1] Frisch, M. J.; Trucks, G. W.; Schlegel, H. B.; Scuseria, G. E.; Robb, M. A.; Cheeseman, J. R.; Scalmani, G.; Barone, V.; Mennucci, B.; Petersson, G. A. Gaussian 09, Revision A.01; Gaussian, Inc.: Wallingford CT, 2009.

[2] Boonnab, S.; Chaiwai, C.; Nalaoh, P.; Manyum, T.; Namuangruk, S.; Chitpakdee, C.; Sudyoadsuk, T.; Promarak, V. *Eur. J. Org. Chem*. **2021**, *2021*, 2402. doi: org/10.1002/ejoc.202100134.

[3] Wang, B.; Zheng, Y.; Wang, T.; Ma, D.; Wang, Q. *Org. Electron.* **2021**, *88*, 106012. doi: org/10.1016/j.orgel.2020.106012
